# Assessment of coding region variants in Kuwaiti population: implications for medical genetics and population genomics

**DOI:** 10.1038/s41598-018-34815-8

**Published:** 2018-11-08

**Authors:** Sumi Elsa John, Dinu Antony, Muthukrishnan Eaaswarkhanth, Prashantha Hebbar, Arshad Mohamed Channanath, Daisy Thomas, Sriraman Devarajan, Jaakko Tuomilehto, Fahd Al-Mulla, Osama Alsmadi, Thangavel Alphonse Thanaraj

**Affiliations:** 1 Dasman Diabetes Institute, P.O. Box 1180, Dasman, 15462 Kuwait; 2 Present Address: Radboud University Medical Center, Nijmegen, The Netherlands; 3 Present Address: Department of Cell Therapy and Applied Genomics, King Hussein Cancer Center, Amman, Jordan

## Abstract

Consanguineous populations of the Arabian Peninsula have been underrepresented in global efforts that catalogue human exome variability. We sequenced 291 whole exomes of unrelated, healthy native Arab individuals from Kuwait to a median coverage of 45X and characterised 170,508 single-nucleotide variants (SNVs), of which 21.7% were ‘personal’. Up to 12% of the SNVs were novel and 36% were population-specific. Half of the SNVs were rare and 54% were missense variants. The study complemented the Greater Middle East Variome by way of reporting many additional Arabian exome variants. The study corroborated Kuwaiti population genetic substructures previously derived using genome-wide genotype data and illustrated the genetic relatedness among Kuwaiti population subgroups, Middle Eastern, European and Ashkenazi Jewish populations. The study mapped 112 rare and frequent functional variants relating to pharmacogenomics and disorders (recessive and common) to the phenotypic characteristics of Arab population. Comparative allele frequency data and carrier distributions of known Arab mutations for 23 disorders seen among Arabs, of putative OMIM-listed causal mutations for 12 disorders observed among Arabs but not yet characterized for genetic basis in Arabs, and of 17 additional putative mutations for disorders characterized for genetic basis in Arab populations are presented for testing in future Arab studies.

## Introduction

Characterising the patterns of genetic variation within and among human populations is crucial to understand human evolutionary history and the genetic basis of disorders^1^. Many global genome-wide genotyping and whole-genome sequencing studies (such as the Human Genome Diversity Project^1,2^, the 1000 Genomes Project (1KGP)^3,4^ and the UK10K project^5^) have been undertaken to catalogue genetic variation. Coding exonic regions, though estimated to encompass only approximately 1–2% of the genome, harbour the most functional variation and contain almost 85% of the known disease-causing pathogenic variants^6,7^; therefore, several global whole-exome sequencing studies have also been undertaken^8–10^. Such large-scale global projects have revealed that human populations harbour a large amount of rare variations which exhibit little homology between diverged populations^3,9–17^, Mendelian and rare genetic disorders are often associated with rare coding variants. Likewise, common markers associated with complex disorders too can vary in frequency across populations^18^. Considering that population-specific differences in allele frequencies are of clinical importance, it is fundamental to catalogue them in diverse ethnic populations^19^.

The Arabian Peninsula holds a strategic place in the early human migration routes out of Africa^20–22^. The Peninsula was instrumental in shaping the genetic map of current global populations because the first Eurasian populations were established here^23^. The ancestry of indigenous Arabs can largely be traced back to ancient lineages of the Arabian Peninsula^23,24^. The Arab population is heterogeneous but well-structured^3,24–26^. For example, the Kuwaiti population comprises three genetic subgroups, namely KWP (largely of West Asian ancestry representing Persians with European admixture), KWS (city-dwelling Saudi Arabian tribe ancestry) and KWB (tent-dwelling nomadic Bedouins characterised by the presence of 17% African ancestry)^24^. Further, the Qatari population also comprises similar subgroups with the third group displaying a much higher African ancestry^25^. The Greater Middle Eastern Variome study^26^ detected several ancient founder populations and continental & sub-regional admixture in the extended region of Greater Middle East (comprising the Gulf region, North Africa and Central Asia); the study further stated that the ancestral Arab population from Arabian Peninsula could be observed in nearly all of the GME regions possibly as a result of the Arab conquests in the seventh century.

Consanguinity in the Arab region has made the population vulnerable to a plague of recessive genetic disorders. An increased burden of runs of homozygosity has been observed in populations from Kuwait^24^ and the extended region of Greater Middle East^26^. An overwhelming proportion (63%) of the disorders documented in the Catalogue for Transmission Genetics in Arabs (CTGA)^27^ follows a recessive mode of inheritance^28^. Studying consanguineous populations lead to identifying causal mutations for Mendelian disorders^29,30^ and rare familial (monogenic) forms of common complex disorders^31^. These studies also paved the way to evaluate the role of consanguinity and environmental factors in complex lifestyle disorders, such as obesity and type 2 diabetes, cases of which are rapidly increasing in the Arabian Peninsula^32,33^. Thus, studying consanguineous populations is important to human medical genetics research^26,34,35^.

Despite consanguinity, diversity and admixture in its populations, the region is poorly represented in global genomic surveys. Even larger databases, such as the Exome Aggregation Consortium (ExAC)^8^ and the Genome Aggregation Database (gnomAD)^8^, are deficient in representing Middle Eastern populations. Although the Greater Middle East (GME) Variome project^26^ provides whole exome data of 1,111 individuals from six GME regions, the region of Arabian Peninsula is represented by only 214 samples and the sub-region of Kuwait by only 45 samples.

In our previous studies, we sequenced and analysed thirteen exomes from the KWS group^36^ and representative whole genomes from each of the three subgroups of the Kuwaiti population^36–38^. In this study, we extended the study by sequencing whole exomes of 291 native Kuwaiti Arab individuals representing the three population subgroups. We further analysed the data to infer the extent of exome variability in the Kuwaiti population and to delineate its impact on population substructures of Kuwait and medical genetics of the region.

## Results

### Exome variants discovered in the Kuwaiti population

The 291 exomes were sequenced to a median coverage of 45X, with an average of 80% of the target base pairs having at least 15X coverage. ‘Missingness’ rate (referring to the percent of samples where information was missing) of 1.8% was obtained leading to genotyping call rate of 98.2%. Totally, 173,849 (including 2,626 non-autosomal) variants were identified (Table 1 and Supplementary Table S1), 12.16% of which were novel. The call set included 170,508 single-nucleotide variants (SNVs) and 3,341 insertions and deletions (indels). 11.85% of the SNVs and 28% of the indels were novel. The observed aggregate transition/transversion (Ti:Tv) ratio of 3.22 was within the acceptable range for whole-exome sequencing variants^39,40^. A heterozygous to homozygous variant genotype ratio of 0.63 was obtained indicating that the population skews towards homozygosity with its inbreeding nature.

**Table 1:** Statistics of variants observed in Kuwaiti exomes.

	All variants		Kuwaiti ‘population-specific’ variants^@^		Kuwaiti ‘Population-specific’ variants seen in ≥2 individuals from the study cohort	
	Number of variants	Average number of variants per individual	Number of variants	Average number of variants per individual	Number of variants	Average number of variants per individual
Total variants (% novel)	173849 (12.16)	14767	57691 (36.63)	365	9870 (34.73)	189
SNVs (% novel)	170508 (11.85)	14557	55644 (36.3)	331	9429 (35.03)	168
Indels (% novel)	3341 (28.07)	210	2047 (45.72)	34	441 (28.34)	21
Ti:Tv	3.22	3.374	2.7	2.44	2.7	2.37
‘Personal’ SNVs^#^	37044	129	37044	129	0	0
Rare SNVs (excluding ‘personal’ variants)	86446	908	17579	109	8408	74
Low-frequency SNVs	21277	1553	883	27	883	27
Common SNVs	25741	11969	138	67	138	67
Missense SNVs^$^	91204	9187	33504	184	5559	87
Synonymous SNVs^$^	70955	10385	19159	126	3377	70
Stop gain SNVs^$^	1425	65	721	3	113	1
Stop loss SNVs^$^	95	9	22	0	4	0
LoF^&^ SNVs^$^	1645	73	843	4.5	131	2

Ti:Tv, transition/transversion ratio.

^@^Variants from our cohort that were not seen in 1KGP were termed as ‘Kuwaiti population-specific’ variants.

^#^Personal SNVs are those that are observed only in a single exome from the study cohort and not seen in the data sets of 1KGP or GME. These are indeed “private mutations” and remain so until the mutations are observed in further exomes/genomes sequenced in future studies.

^&^Loss-of-function (LoF) variants represent the sum total of stop gain, stop loss, frameshift and splicing variants. LoF variants are expected to correlate with complete loss of function of the affected transcripts, including stop codon-introducing (nonsense) or splice site-disrupting single-nucleotide variants (SNVs), insertion/deletion (indel) variants predicted to disrupt a transcript’s reading frame or larger deletions removing either the first exon or >50% of the protein-coding sequence of the affected transcript.

^$^These were calculated using all the identified SNVs including the personal variants.

### Validation of SNP calls

The validity of the SNP calls was confirmed by utilizing an in-house genome-wide genotype data set on 269 (of the sequenced 291) samples derived using the Illumina HumanOmniExpress BeadChip (Illumina Inc, USA). In an average, 13,175 variants could be compared per sample. The concordance rate of the SNP calls between the exome sequencing data and genome-wide genotype data was >99.7% (see Supplementary Table S2). The observed concordance rate in our study is on par with those reported in literature: Kenna *et al*.^40^ reported a genotype concordance rate of 98.9% on comparing the accuracy of genotypes inferred using Illumina high throughput sequencing platforms with genotypes ascertained using Illumina BeadChips. The disagreements in the SNP calls were seen more often with heterozygous SNPs than with homozygous SNPs. As is the practice^41^, we choose not to remove the inconsistent calls.

### Principal component analysis of variants in the merged set of exome variants from Kuwait and global populations

**Figure 1:**
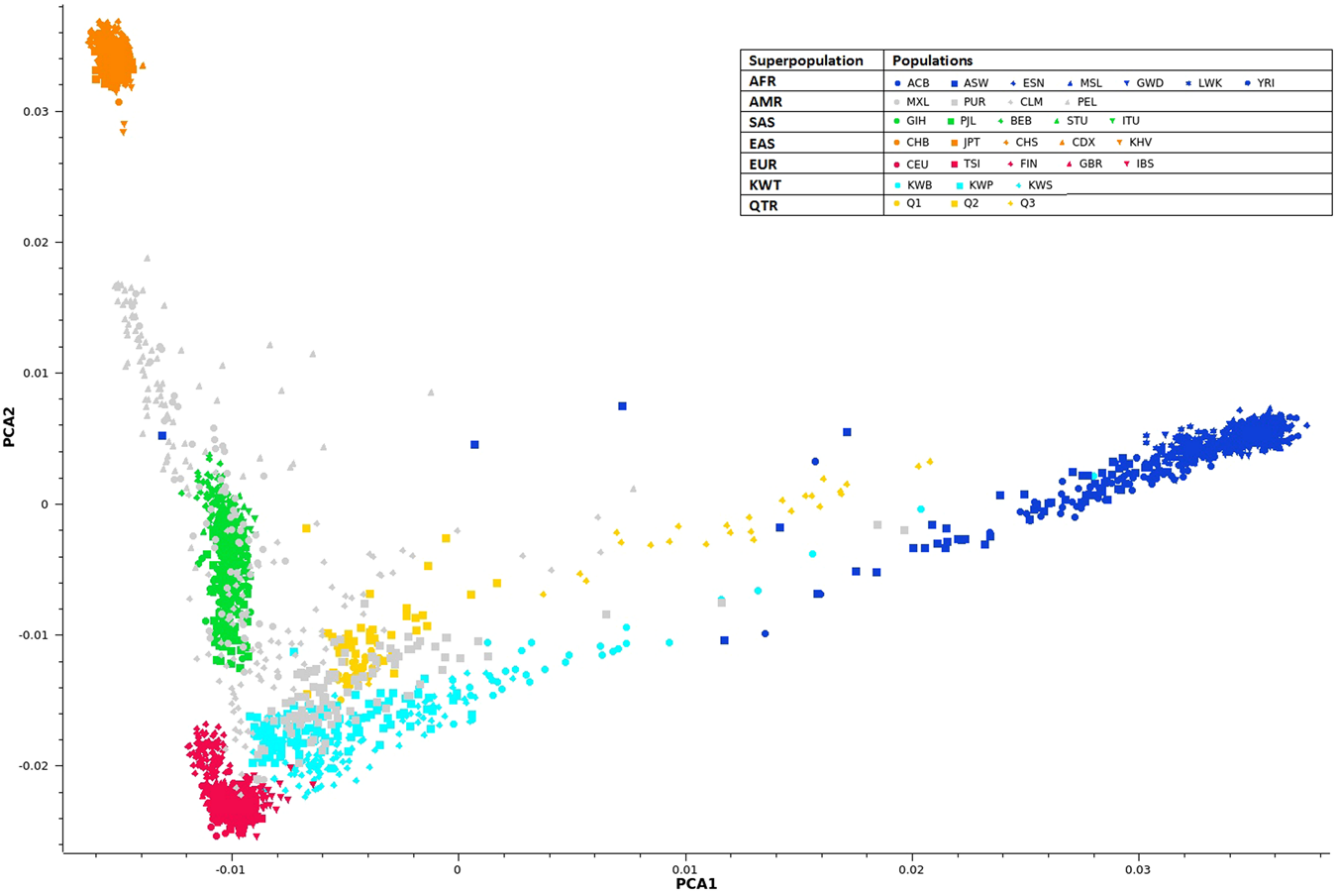
Scatter plot of the first two principal components of the merged data set of exome variants from the three Kuwaiti substructures and from regional (Qatar) and 1KGP global populations.

The scatter plot of the first two principal components of the merged data set of exome variants from Kuwait, 1KGP global populations, and Qatar is presented in Fig. 1. The plot affirmed the heterogeneity of the Kuwaiti Arab population as comprising three substructures^24^ and inferred the regional affinity.

### Classifications of observed SNVs

50.7% of the identified SNVs were ‘rare’, 12.5% were ‘low-frequency’ and 15.1% were ‘common’. Up to 21.7% of the SNVs were ‘personal’ (found in only one Kuwaiti exome and not seen in the data sets of 1KGP Phase 3 and GME). Alternate allele was the major allele in 4.2% of the identified SNVs; 0.22% of the SNVs were fixed for the alternate allele, having a non-reference frequency of 100%. Among the identified SNVs, 53.5% were missense, 41.61% were synonymous and approximately 1% were loss-of-function (LoF). 55,644 of the identified SNVs were ‘population-specific’, 60% of which were missense; 9,429 of these 55,644 population-specific variants were polymorphic (seen in ≥2 exomes from the study cohort and not seen in 1KGP), most of which were ‘rare’ (8408 out of 9429); of the remaining 46,215 variants, 37,044 were ‘personal’ and 9171 were seen in one exome from the study cohort and were also seen in GME data set. On average, 14,557 SNVs and 210 indels were seen in every Kuwaiti individual. The average number of ‘personal variants’ per individual was 129. Population-specific missense variants per individual were more than synonymous changes (184 *versus* 126). The average number of LoF variants per Kuwaiti individual was 73, of which 4.5 were specific to the Kuwaiti population.

### Homozygous LOF variants and “inactivated genes”

We had observed 1645 putative LoF SNVs (Table 1) in Kuwaiti exomes from 291 healthy individuals of Arab ethnicity. 186 of these 1645 LoF SNVs were homozygous and they were harbored in 179 genes (See Supplementary Table S3). Of the 186 homozygous LoF SNVs, 27 were with MAF <1% and another 9 were with MAF (≥1% and <2%). Sulem *et al*.^42^, by way of performing whole-genome sequencing of 2,636 Icelanders and chip-imputing a further 101,584 Icelanders, had identified a set of rare (MAF <2.0%) homozygous LoF variants in 1,171 genes. In a similar manner, the Exome Aggregation Consortium (ExAC) data set of 60,706 sequenced individuals identified 2,068 genes that were inactivated^8^; The GME^26^ consortium, by way of analyzing 354 exomes of healthy individuals, identified 301 genes with rare homozygous LoF variants of which 50 genes overlapped the Icelandic gene list and 94 overlapped the ExAC gene list of inactivated genes. Upon comparing the homozygous LoF variants from the Kuwaiti exomes with the above-mentioned data sets of inactivated genes, we found 23 genes (PNPLA1, ULBP3, OR8K3, RAD52, APOBEC1, PDIA2, WDR87, SIGLEC1, COL9A2, OTOF, SULTIC3, COQ2, MROH2B, FAM81B, UNC93A, DNAH11, PXDNL, OR4D10, SLC22A24, RNASE9, C17orf77, CARD14 and SLC5A4) in common with Icelandic data set, 3 genes (EML1, WWTR1 and PPFIA1) in common with ExAC data set and six genes (COL9A2, SLC5A9, FAM81B, GGT6, EFCAB13 and SLC5A4) in common with GME data set. Upon considering only those LoFs with <2% MAF in Kuwaiti exomes, the number of genes in common with Icelandic data got reduced to 8 - PNPLA1 (MAF_KWT of the LoF: 1.03), ULBP3 (0.52%), OR8K3 (1.5%), RAD52 (1.3%), APOBEC1 (0.34%), PDIA2 (1.3%), WDR87 (0.34%) and SIGLEC1 (0.34%)); upon considering only the rare (MAF <1%) homozygous LoF variants in Kuwaiti exomes, only one gene (EML1 (0.69%)) was seen in common with ExAC data set; and none with GME data set. GME work reported more genes as common with the Icelandic/ExAC data sets as they also considered indels along with SNVs to derive the list of LOFs while we considered only the SNVs.

### Comparison with Greater Middle East (GME) Variome data

Results of comparing the variants observed in our study with those reported in GME populations^26^ are presented in Table 2. Up to 64% of the SNVs identified in our study were seen common with GME – the remaining 36% of variants not seen in GME are expected to enlarge the variome of the GME region. GME provided supporting evidence to designate up to 25% of Kuwaiti population-specific singleton mutations (seen in only one exome from the study cohort) as genuine SNVs. Up to as high as 58% of the population-specific polymorphic variants observed in Kuwaiti exomes were also seen in GME variome.

**Table 2:** Comparing the Kuwaiti Arab whole-exome variants with Greater Middle East (GME) whole-exome Variome.

SNV Category	Total observed in Kuwaiti Arabs	Present in GME variome	Present only in the “Arabian Peninsula” subregion of GME
All	170508	109058 (64%)	4351
All:Personal	37044	0 (0%)	0
Kuwaiti “Population-specific”	55644	14660 (26.3%)	2393
Kuwaiti “Population-specific polymorphic” variants seen in ≥2 individuals from the study cohort	9429	5474 (58%)	793

### Extent of variability in Kuwaiti exomes

**Figure 2:**
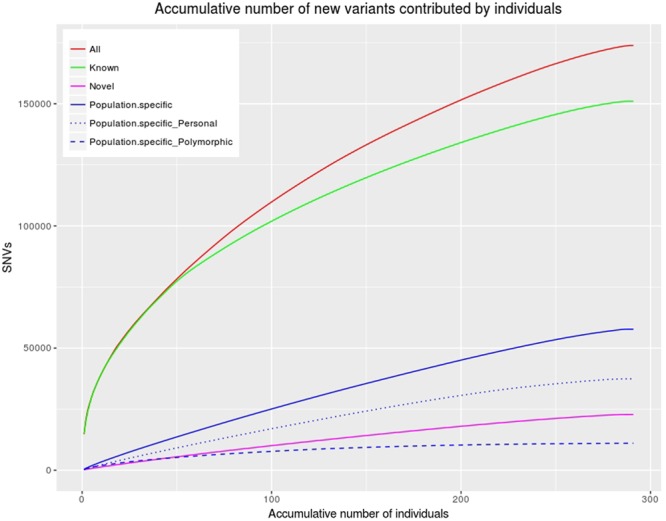
Distribution of total number of single-nucleotide variants (SNVs) upon step-wise addition of exomes. The red line represents the number of all variants found as the number of sequenced exomes increased. The green line represents the number of known variants among all variants found. The orange line represents the number of novel variants among all variants found. The blue line represents the number of population-specific variants among all variants found. Population-specific ‘personal’ variants observed in only one Kuwaiti exome and not seen in either 1KGP or GME are represented by the dotted line; population-specific ‘polymorphic’ variants observed in more than one Kuwaiti exome are represented by the dashed line.

In each of the categories of ‘all’, ‘known’, ‘novel’ and ‘Kuwaiti population-specific’ variants, the observed number of variants increased linearly with increasing number of sequenced exomes and did not reach a plateau (Fig. 2). A similar trend was observed when the three subgroups were examined individually (Supplementary Fig. S1). However, when the population-specific variants were divided into ‘personal’ and ‘population-specific polymorphic’ variants, population-specific variants shared by more than one individual reached a plateau.

### Variants significantly differentiating the three population subgroups of Kuwait

Results of *pFst* likelihood ratio tests for allele frequency differences between the three subgroups based on 142,626 autosomal variants are presented in Supplementary Fig. S2. Three variants significantly distinguished KWP from KWB: rs2289043_A > G (*UNC5C*) (*pFst* = 3.28 × 10^−6^), mostly prevalent in admixed Americans (75%) and Europeans (71%); rs3739310_T > G (*KIAA1456*) (*pFst* = 4.40 × 10^−5^), frequently found in East Asians (78%) and Europeans (77%); and rs764374986_G > A (*AKAP12*) (*pFst* = 5.21 × 10^−5^), a rare variant occurring mostly in Africans (0.01%) from gnomAD data set (the variant is absent in 1kGP data set). Three variants significantly distinguished KWS from KWB:rs1150360_A > G (*FAM76B*) (*pFst* = 3.89 × 10^−5^), frequent in Africans (93%); rs138408584_G > C (*PHRF1*) (*pFst* = 6.97 × 10^−5^), rare in Europeans (~1%); and rs1043730_G > T (*TRAF3IP2*) (*pFst* = 9.57 × 10^−5^), present at 99% frequency in Africans and East Asians. Two variants significantly distinguished KWP from KWS: rs35840170_C > T (*FBN3*) (*pFst* = 7.63 × 10^−5^), present at ~20% frequency in East Asians and admixed Americans; and rs7956133_G > T (*FAM216A*) (*pFst* = 9.51 × 10^−5^) present at a frequency of 15% in Africans.

### SAFD variants with significant allele frequency differences between the Kuwaiti and 1KGP global populations; and analysis of their population-wide occurrence

**Figure 3:**
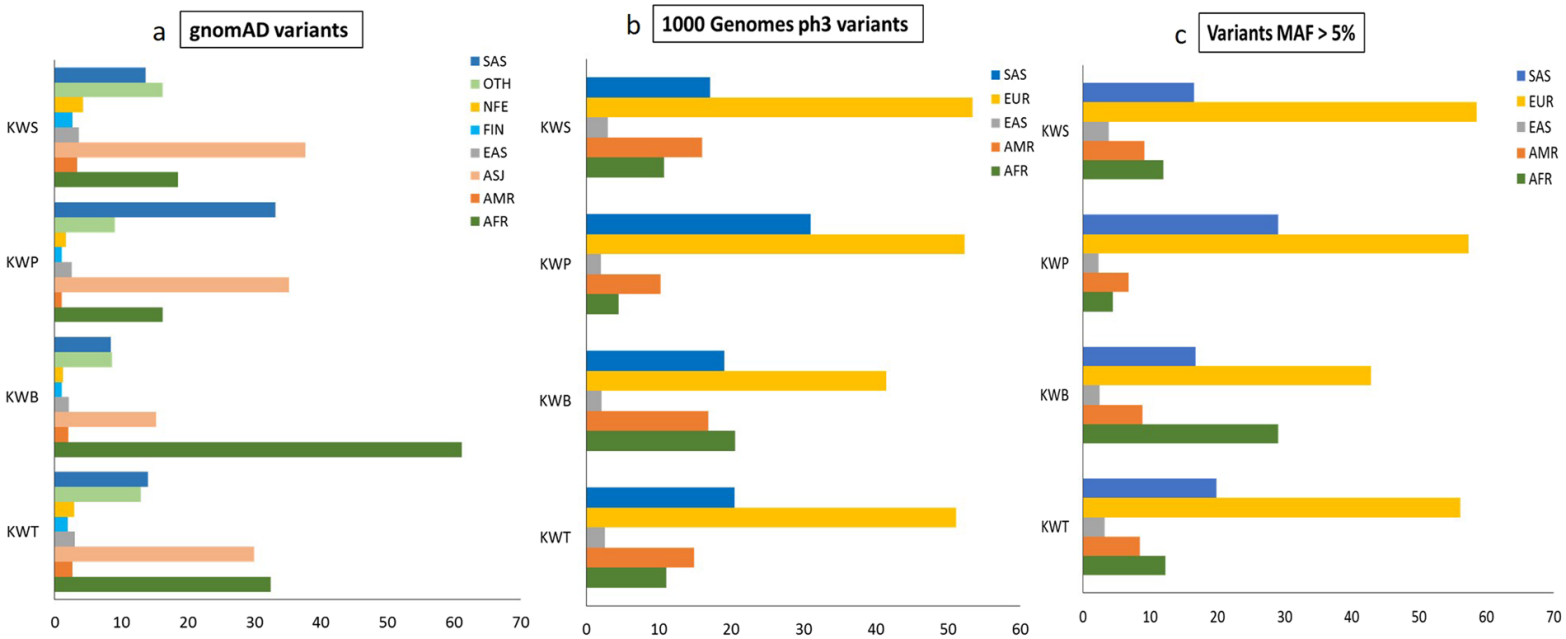
The occurrence pattern of pairing Kuwaiti populations with (**a**) the gnomAD or (**b**) the 1KGP global populations as populations with maximum allele frequency. X-axis: percentage of pairing occurrence of Kuwaiti populations and gnomAD (A) or 1KGP (B) global populations as populations with maximum allele frequency, Y-axis: Kuwaiti populations (KWT-All Kuwaitis; KWB-Bedouins; KWP-Persians; KWS-Saudi Arabian tribe). gnomAD global populations: AFR, Africans/African Americans; AMR, admixed Americans; ASJ, Ashkenazi Jewish; EAS, East Asians; FIN, Finnish; NFE, Non-Finnish Europeans; OTH, Other population not assigned; SAS, South Asians. 1KGP global populations: AFR, African; AMR, Admixed American; EAS, East Asian; EUR, European; SAS South Asian. (**c**) Considers only the variants with minor allele frequency (MAF) of >5% (n = 3887) and pairing with 1KGP global populations.

Examination of the SNVs seen in common between Kuwaiti exomes and 1KGP phase 3 exome data for significant allele frequency differences led to identifying 6,186 SAFD variants. Functional characterization of these variants is presented in Supplementary Table S4. Of these 6,186 SAFD variants, 2,960 were missense, 2,913 were synonymous, 20 were stop-gain and 26 were LoF. Extent of LoF variants among the SAFD SNVs was only 0.4% while it was 1.7% among the ‘all’ SNVs. Population-wide occurrence of the identified SAFD variants was investigated to determine the pairing occurrence of Kuwaiti population subgroups in the context of maximum allele frequency (Supplementary Fig. S3 and Fig. 3). (a) Analysis of the 5,140 SAFD variants, derived using gnomAD populations: The number of variants showing maximum allele frequency in KWS, KWP and KWB subgroups were 2885, 543 and 1712, respectively. In KWS, 38% of the 2885 variants showed maximum allele frequency in Ashkenazi Jews and 19% in Africans. In KWP, 35% had maximum allele frequency in Ashkenazi Jews and 33% in South Asians. In KWB, 61% variants showed maximum allele frequency in Africans. (b) Analysis of 6186 SAFD variants, derived using 1KGP populations: Coupling observed with South Asians and Africans was confirmed. KWB paired with Africans in 21% of 1,056 variants; KWP and KWS paired with South Asians in 31% of 1355 and 17% of 3775 variants, respectively. Furthermore, coupling with Europeans, which was not seen in the analysis using gnomAD populations, was observed in 53% of KWS variants, 52% of KWP variants and 41% of KWB variants.

### Validation of the genetic relatedness implied by analysis for population-wide occurrence of SAFD variants

**Figure 4:**
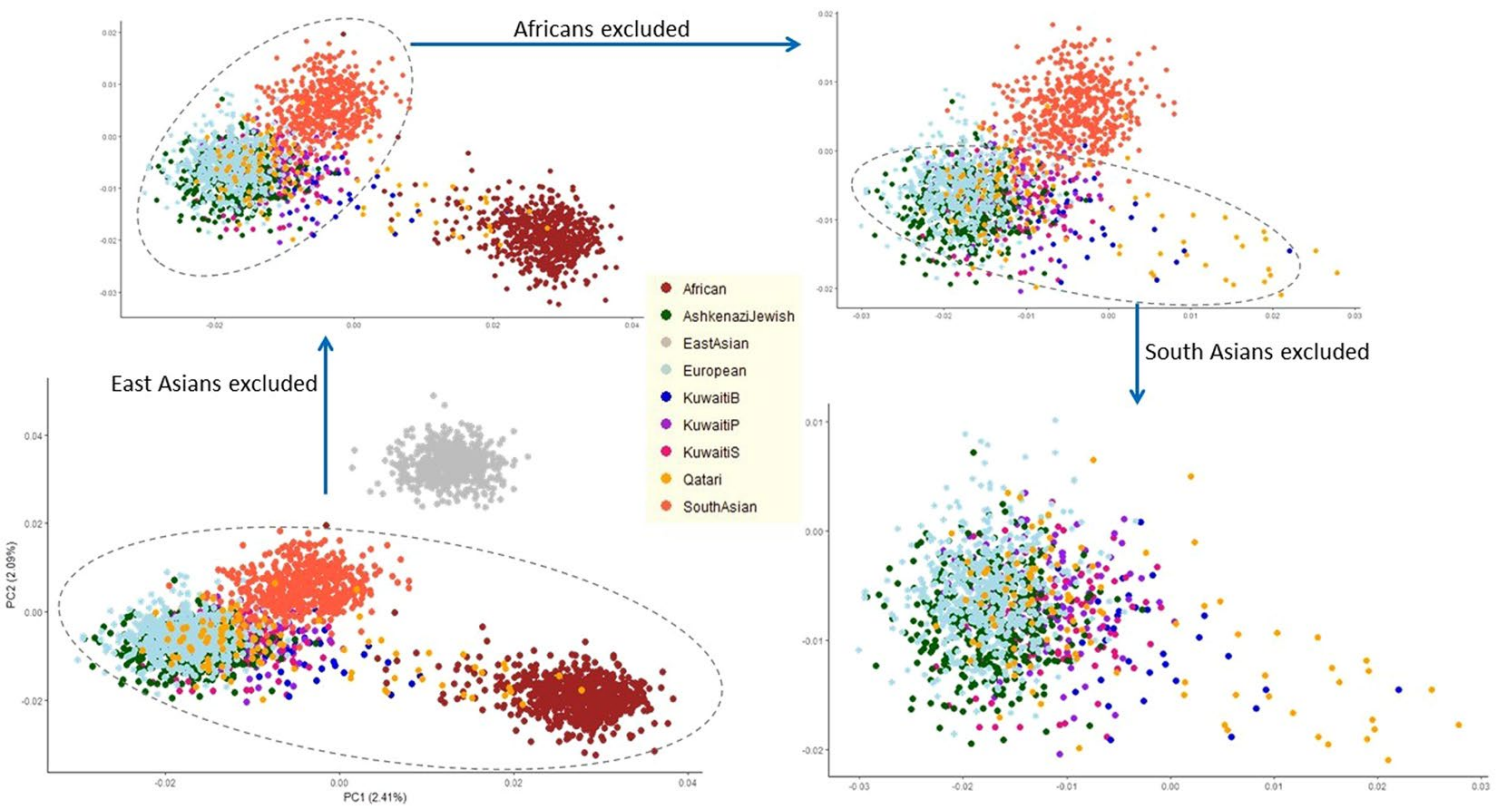
Two-dimensional principal component analysis (PCA) plots showing the dispersal of Kuwaitis over the Qataris, Ashkenazi Jewish and Europeans.

**Figure 5:**
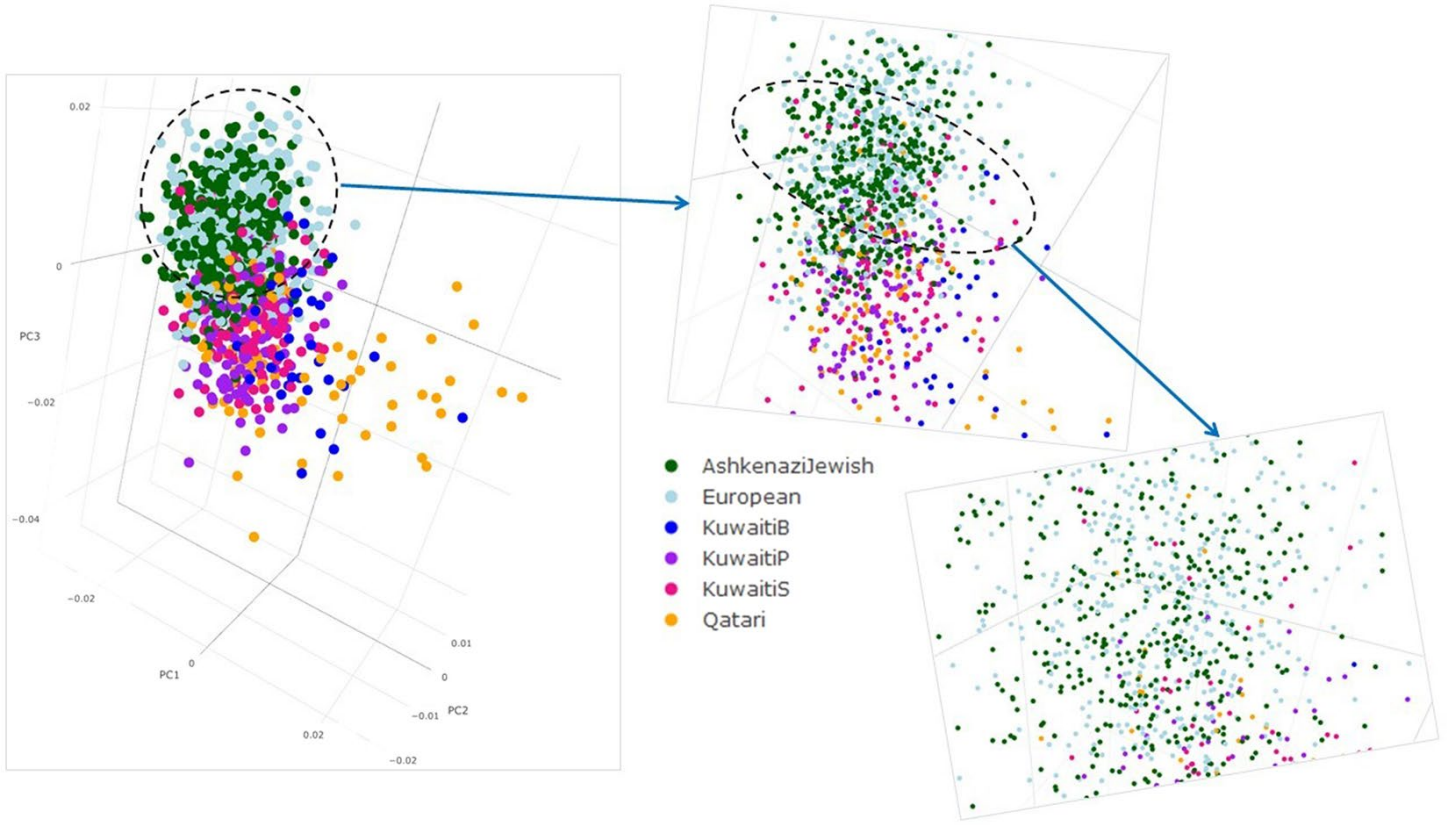
Three-dimensional principal component analysis (PCA) plots showing the dispersal of Kuwaitis over the Qataris, Ashkenazi Jewish and Europeans. The interactive three-dimensional plot is available at http://dgr.dasmaninstitute.org/exome_pca/).

In order to further explore the observed coupling in maximum allele frequency between Kuwaitis and other populations (including the Ashkenazi Jews), Kuwaiti exome data was merged with the data sets from Ashkenazi Jews^43^, Qatar^44^ and 1KGP phase 3. Upon applying quality control steps and LD-pruning the combined data set of coding-region variants, a total of 896 variants from 3,336 individuals was obtained. Genetic differentiation of Kuwaiti subpopulation groups in terms of regional and continental populations was assessed by way of calculating mean pairwise *F*_ST_ (Supplementary Fig. S4, Supplementary Table S5). Lowest degree of differentiation was observed between Kuwaiti subpopulation groups and Qataris (KWB *F*_ST_ = 0.0005, KWP *F*_ST_ = 0.0027, KWS *F*_ST_ = 0.0023) followed with Ashkenazi Jews (KWB *F*_ST_ = 0.0103, KWP *F*_ST_ = 0.0071, KWS *F*_ST_ = 0.0104) and Europeans (KWB *F*_ST_ = 0.0143, KWP *F*_ST_ = 0.0093, KWS *F*_ST_ = 0.0155). Scatter plots resulting from principal component analysis (PCA) of the merged data set are presented in Figs 4 and 5. Consistent with the *F*_ST_ analysis, the Kuwaiti population were seen dispersed over the Qataris, Ashkenazi Jewish and Europeans (Fig. 4). A clear dispersal of these populations was seen in the three-dimensional PCA plot (Fig. 5 and the interactive three-dimensional plot available at http://dgr.dasmaninstitute.org/exome_pca/).

### ‘Rare and deleterious’ variants and their clinical significance

The analysis pipeline that examined the Kuwaiti exomes for deleterious variants which are rare in both 1KGP and ExAC data sets yielded a list of 46 variants (41 unique disorders of which 20 were reported in CAGS database) – comprising 35 pathogenic (for rare disorders), 1 drug response, 10 risk factors (1 corresponding to a rare but multifactorial disorder and the remaining 9 to complex and common disorders) (Table 3). Of these 46 variants, 43 variants remained rare in Kuwaiti exomes; and three variants reached an MAF value characterizing low-frequency variants (rs1800553/*ABCA4*/Risk-Factor:2.41%, rs61742245/*VKORC1*/Drug-response:1.04%, rs11909217/*LIPI*/Risk-Factor:1.72%). Pathogenic variants: The 35 pathogenic variants mapped to 32 genes and to 32 unique single-gene disorders; 28 of the 35 variants follow autosomal recessive (AR) and the remaining follow autosomal dominant (AD) mode of inheritance. In 16 instances of these 35 pathogenic variants, the disorders were observed in Arab population (as annotated in CAGS database^27^). Drug response variant: the VKORC1 variant was associated with warfarin resistance in AD mode. Risk factor variants: The 10 risk factor variants mapped to 9 genes and to susceptibility to 8 unique disorders. The inheritance patterns were seen to be mostly autosomal dominant (in three instances, can be AR along with AD). In instances of 4 of the 10 risk factor variants, the disorders were observed in Arab population as per CAGS database.

**Table 3:** 46 rare and deleterious variants (pathogenic and risk factors)^@^ seen in Kuwaiti Exomes.

dbSNP; Ref/risk; gene	Inheritance mode (AD:autosomal dominant; AR:autosomal recessive; MF:multifactorial)	Disorder (PMIM); No of OMIM-listed genes for the disorder; annotation pertaining to whether the disorder is rare or common^$^	RAFs: KWT/1KGP/GME; Ratios of RAFs: KWT-1KGP/KWT-GME	Carrier status in Kuwaiti exomes (rr/rR/RR)	Disorder seen in CAGS populations (YES/NO)?
**A**. **PATHOGENIC and rare disorders CAUSAL VARIANTS (n** = **34)**					
rs137853054-G/A; *PARK2*	AR	Parkinson disease, juvenile, type 2 (PMIM:600116)^92^; Single gene; Rare disorder.	0.0034/0.0002/0.0025; 17.0000/1.3503	0/2/288	Yes
rs34424986-G/A; *PARK2*	AR. This and the above both participate in compound heterozygosity	Parkinson disease, juvenile, type 2. (PMIM:600116). Single gene; Rare disorder.	0.0034/0.0004/-; 8.5000/-	0/2/289	Yes
rs121912615-A/C; *SI*	AR. In compound heterozygosity state	Sucrase-isomaltase deficiency (PMIM:222900). Single gene; Rare disorder.	0.0052/0.0004/0.0025; 13.0000/2.0651	0/3/283	No
rs200487396-G/A; *COL12A1*	AD	Bethlem myopathy 2 (PMIM:616471); Single gene; Rare disorder.	0.0018/0.0002/-; 9.0000/-	0/1/279	No
rs143137713-G/C; *GYG1*	AR	Polyglucosan body myopathy 2 (PMIM:616199). Single gene; Rare disorder.	0.0017/0.0004/-; 4.2500/-	0/1/287	No
rs148772854-C/T; *RYR1*	AR. The variant participates in compound heterozygosity.	Minicore myopathy with external ophthalmoplegia (PMIM:255320). Single gene; Rare disorder.	0.0017/0.006/0.0015; 0.2833/1.1243	0/1/290	No
rs61757582-G/A; *DHCR7*	AR	Smith-Lemli-Opitz syndrome (PMIM:270400). Single gene; Rare disorder.	0.0018/0.0002/-; 9.0000/-	0/1/284	Yes
rs61754375-G/A; *TYR*	AR	Tyrosinase-negative oculocutaneous albinism, Type IA (PMIM:203100). Single gene; Rare disorder.	0.0017/0.0002/-; 8.5000/-	0/1/286	Yes
rs61753185-G/A; *TYR*	AR	Tyrosinase-negative oculocutaneous albinism, Type IA (PMIM:203100). Single gene; Rare disorder.	0.0017/0.0004/0.0005; 4.2500/3.3730	0/1/290	Yes
rs104894313-C/T; *TYR*	AR	Oculocutaneous albinism type 1B (PMIM:606952). Single gene; Rare disorder.	0.0017/0.002/0.0049; 0.8500/0.3476	0/1/285	No
rs121434513-G/C; *PNKD*	AD	Paroxysmal Nonkinesigenic Dyskinesia 1 (PMIM: 118800). Single gene; Rare disorder.	0.0017/0.0002/0.0005; 8.5000/3.3531	0/1/289	Yes
rs121964924-A/G; *DPYS*	AR	Dihydropyrimidinase deficiency (PMIM:222748). Single gene; Rare disorder.	0.0017/0.0002/0.0020; 8.5000/0.8424	0/1/289	Yes
rs28940872-C/T; *ACADS*	AR. This variant is seen in compound heterozygosity.	Short-chain acyl-CoA dehydrogenase (SCAD) deficiency (PMIM:606885). Single gene; Rare disorder.	0.0017/0.0002/0.0015; 8.5000/1.1251	0/1/289	Yes
rs148211042-C/T; *ABCB6*	AD	Pseudohyperkalemia, familial, 2, due to red cell leak (PMIM:609153). Single gene; Rare disorder.	0.0017/0.0002/0.0010; 8.5000/1.6882	0/1/290	No
rs58331765-C/T; *ABCA4*	AR	Stargardt disease 1, Juvenile (PMIM:248200)^93^. Single gene; Rare disorder.	0.0034/0.0028/0.0005; 1.2143/6.7460	0/2/288	Yes
rs35152987-C/A; *HBD*	AR.	delta Thalassemia – it is usually associated with a single gene of HBD. It does not have clinical manifestation but is usually associated with beta thalassemia. Thalassemia is a rare disorder.	0.0052/0.001/0.0086; 5.2000/0.6075	0/3/287	No
rs114368325-G/A; *CYP24A1*	AR. This variant is seen in compound heterozygosity.	Idiopathic hypercalcemia of infancy or hypercalcemia, infantile, 1; hcinf1 (PMIM:143880). Single gene; Rare disorder.	0.0017/0.0004/0.0005; 4.2500/3.3730	0/1/290	No
rs538881762-C/T; *TENM4*	AD	Tremor, hereditary essential, 5 (ETM5). (PMIM:616736). Single gene. GARD lists ETM as NOT a rare disorder but lists the subtypes as rare.	0.0018/0.0006/-; 3.0000/-	0/1/276	No
rs116100695-G/A; *PKLR*	AR	Pyruvate kinase deficiency of red cells (PMIM:266200). Single gene; Rare disorder.	0.0034/0.0016/0.0096; 2.1250/0.3547	0/2/289	Yes
rs6063-C/T; *FGG*	AR. Found in double heterozygosity	Fibrinogen Milano XII, digenic; Dysfibrinogenemia (PMIM:616004). Three genes. Rare disorder.	0.0052/0.0028/0.0050; 1.8571/1.0328	0/3/286	No
rs121908736-G/A; *ADA*	AR, SM. The variant can also occur in compound heterozygous state	Partial adenosine deaminase deficiency (PMIM:102700). Single gene; Rare disorder.	0.0035/0.0018/0.0005; 1.9444/6.9444	0/2/285	Yes
rs41295338-G/T; *TGM1*	AR	AR congenital ichthyosis 1 (PMIM:242300). Single gene; Rare disorder.	0.0034/0.0022/0.0060; 1.5455/0.5627	0/2/288	Yes
rs28941785-C/T; *CTH*	AR.	Cystathioninuria (PMIM:219500). Single gene; Rare disorder.	0.0035/0.0026/0.0065; 1.3462/0.5347	0/2/281	Yes
rs77010315-C/A; *SLC36A2*	AR, DR	Iminoglycinuria, digenic (PMIM:242600). Single gene; Rare disorder.	0.0076/0.005/0.0025; 1.5200/3.0159	0/4/258	No
rs56208331-G/A; *GATA4*	AD	Tetralogy of fallot; TOF. (PMIM:187500). Single gene; Rare disorder.	0.0034/0.0034/0.0025; 1.0000/1.3503	0/2/289	Yes
rs121908970-C/T; *MYO15A*	AR. The variant was seen in hemizygosity state.	Deafness, with Smith-Magenis syndrome (PMIM: 600316). Single gene; Rare disorder.	0.0018/0.001/0.0010; 1.8000/1.7875	0/1/274	No
rs34324426-C/T; *PEX6*	AR. The variant is in compound heterozygosity state.	Heimler syndrome 2 (PMIM:616617). Single gene; Rare disorder.	0.0017/0.001/0.0015; 1.7000/1.1243	0/1/290	No
rs104893836-T/C; *GNRHR*	AR. The variant is in compound heterozygosity state.	Hypogonadotropic hypogonadism 7 without anosmia. (PMIM:146110). Single gene; Rare disorder.	0.0017/0.0012/0.0055; 1.4167/0.3069	0/1/290	Yes
rs5907-G/A; *SERPIND1*	AD	Thrombophilia due to Heparin cofactor II deficiency (PMIM:612356). Single gene. Inherited disorder	0.0017/0.0012/-; 1.4167/-	0/1/290	No
rs113418909-A/T; *SERPINA6*	AR,AD	Corticosteroid-binding globulin deficiency (PMIM:611489). Single gene; Rare disorder.	0.0017/0.0014/0.0045; 1.2143/0.3751	0/1/290	No
rs121909293-C/T; *CTRC*	AD	Pancreatitis, chronic, susceptibility to (PMIM:167800). 5 genes. Hereditary Pancreatitis, that leads to chronic form, as rare disorder.	0.0017/0.0016/-; 1.0625/-	0/1/290	No
rs137941190-C/T; *DCPS*	AR. Found in compound heterozygous state.	Al-raqad syndrome (PMIM:616459). Single gene; Rare disorder.	0.0017/0.002/0.0005; 0.8500/3.3730	0/1/288	No
rs28940885-C/T; *GALE*	AR/AD heterogeneity	Galactose Epimerase Deficiency (PMIM:230350). Single gene; Rare disorder.	0.0017/0.002/0.0025; 0.8500/0.6751	0/1/290	Yes
rs73015965-A/G; *PLG*	AR. Participates in compound heterozygosity	Plasminogen deficiency, type I (PMIM:217090). Single gene; Rare disorder.	0.0017/0.0022/0.0006; 0.7727/2.8286	0/1/289	No
rs200879436-T/C; *CEP152*	AR. The variant is seen in compound heterozygous state	Seckel syndrome 5 (PMIM:613823). Single gene; Rare disorder.	0.0017/0.0034/0.0015; 0.5000/1.1251	0/1/288	No
**DRUG RESPONSE (n** = **1)**					
rs61742245-C/A; *VKORC1*	AD	Warfarin resistance (PMIM:122700). Single gene; Polymorphisms in other genes, some of which have not been identified, have a smaller effect on warfarin metabolism. Rare disorder. Poor quality of anticoagulation with warfarin has been reported across Kuwait^94^	0.0104/0.0004/0.0137; 26.0000/0.7619	1/4/284	No
**C**. **Risk Factor and rare disorders**					
rs139512218-G/T; *SPRY4*	AD	Hypogonadotropic hypogonadism 17 with or without anosmia (PMIM: 615266). Single gene. CHH is a rare reproductive disorder but multifactorial (involving genes of FGF pathway).	0.0035/0.0024/0.0010; 1.4583/3.4757	0/2/282	No
**D**. **RISK FACTORS (n = 9) – Common or multifactorial complex disorders**					
rs11554495-C/A; *KRT8*	AR	Cirrhosis, cryptogenic (PMIM:215600). **Two genes**. ClinVar annotation is pathogenic but we annotate it as risk factor as this disorder is probably common. GARD does not list this as rare disorder as it affects more than 600,000 people in USA	0.0053/0.001/0.0027; 5.3000/1.9910	0/3/281	No
rs1800553-C/T; *ABCA4*	AD	Macular degeneration, age-related, 2 (AMD2), susceptibility to (PMIM:153800). Single gene; however, AMD is a complex trait. GARD lists this as NOT a rare disorder. ClinVar annotates this as pathogenic’; however, it is a susceptibility variant for a complex trait and hence it is risk factor.	0.0241/0.0032/0.0211; 7.5313/1.1396	1/12/278	No
rs11909217-C/T; *LIPI*	AD	Susceptibility to Hypertriglyceridemia, Familial. (PMIM: 145750). Two genes -*LIPI* and *APOA5*. Arab study reports a different variant 3’ UTR C > G from APOC3^95^. This is an inherited common disorder.	0.0172/0.006/0.0131; 2.8667/1.3138	0/10/280	Yes
rs114817817-C/T; *SRGAP1*	AD	Thyroid cancer, nonmedullary 2, susceptibility to (PMIM:188470). Three genes. Most common form of thyroid cancer.	0.0017/0.0006/0.0070; 2.8333/0.2412	0/1/285	Yes
rs72470545-G/A; *HTRA2*	AD	Parkinson disease 13, Autosomal Dominant, susceptibility to (PMIM: 610297). Single gene. It is a complex neuro-degenerative disorder.	0.0069/0.0036/0.0070; 1.9167/0.9789	0/4/287	No
rs28932472-G/C; *POMC*	AR,AD,MF	Obesity, early-onset, susceptibility to (PMIM: # 601665). Several genes. Common disorder. Arab study reports another variant rs1042713 (Arg16Gly) in ADRB2 gene^96^ for this disorder; which is associated with susceptibility to nocturnal asthma in OMIM and our study.	0.0017/0.0024/0.0005; 0.7083/3.3730	0/1/287	Yes
rs34911341-C/T; *GHRL*	AR,AD,MF	Obesity, susceptibility to (PMIM: 601665). Several genes. Common disorder.	0.0017/0.0026/0.0035; 0.6538/0.4823	0/1/290	Yes
rs138292988-G/A; *AP1S3*	AD	Psoriasis 15, pustular, susceptibility to (PMIM:616106). Single gene. Psoriasis is NOT a rare disorder; pustular psoriasis is a rare form Psoriasis. Susceptibility is the keyword.	0.0017/0.0024/0.0015; 0.7083/1.1251	0/1/289	No
rs116107386-A/C; *AP1S3*	AD	Psoriasis 15, pustular, susceptibility to (PMIM:616106). Single gene. Psoriasis is NOT a rare disorder; pustular psoriasis is a rare form Psoriasis.	0.0018/0.003/0.0050; 0.6000/0.3575	0/1/279	No

All the 46 variants were seen annotated in OMIM and ClinVar for clinical significance.

^@^Clinical significance of a variant was checked by way of using the evidences presented in OMIM and ClinVar (see Methods).

^**$**^Various resources that were examined to ascertain whether the disorder is rare or common: Catalogue of Transmission Genetics in Arabs (available at http://cags.org.ae/ctga/), Genetic and Rare Disease (GARD) Information Centre (available at https://rarediseases.info.nih.gov/diseases/), Genetics Home Reference (available at https://ghr.nlm.nih.gov/), Medscape (available at https://geneaware.clinical.bcm.edu/GeneAware/AboutGeneAware/DiseaseSearch.aspx) and literature.

#### Pathogenic variants and high MAF in Kuwaiti exomes

Five of the identified “rare & deleterious” variants that were annotated “pathogenic” for clinical significance in ClinVar were seen to possess risk allele frequencies of ≥1% in Kuwaiti exomes as opposed to <1% in 1KGP populations. ClinVar defines “pathogenic” variants as those that are interpreted for Mendelian disorders; or as those that have low penetrance. It is also possible that a variant in ClinVar can have an erroneous or conflicting classification. Cassa *et al*.^45^ examined 81,432 “pathogenic” variants from HGMD^7^ in a data set of whole-genome sequences of 1.092 individuals from 1KGP project and found that 4.62% of the tested variants to possess an MAF of ≥1% and 3.5% of the tested variants to possess an MAF of ≥5%; they concluded that many of these variants are probably erroneous findings or have lower penetrance than previously expected. It is also possible that such high frequency pathogenic variants are indeed of the type “increased susceptibility” and not of the type “causal”; it is also possible that the disorders with such high MAF “pathogenic” variants are not really “rare” but are either “common” or “more prevalent in the study population”; that it is also possible that the frequent variants have evidence to cause a disease when inherited in compound heterozygous state and have insufficient evidence to lead to a disease in homozygotes. The five “pathogenic” variants that were seen in Kuwaiti exomes with an MAF of ≥1% are as follows:

*(a) Four variants retained as pathogenic for rare disorders:* (i) rs79204362 (MAF_KWT:1.03% and MAF_1KGP: 0.42%) associated with Early onset of Glaucoma: ClinVar annotated this variant as Pathogenic based on evidence from literature studies and as of uncertain significance based on clinical testing. The disorder was supposed to be rare (1 in 10,000) in European-based populations and of higher frequency in Middle East 51–100 per 100,000 (*i*.*e*. 5 in 10,000); CTGA reported a high incidence rate of 1 in 2,500 in Saudi Arabian population. Thus, the MAF that is seen marginally higher at 1.03% was acceptable. (ii) rs61732874_C > A (MAF_KWT:1.55% and MAF_1KGP: 0.18%) associated with Familial Mediterranean fever (FMF): ClinVar annotated this as Pathogenic/likely-pathogenic based both on literature evidence and clinical testing. FMF is a rare disorder in European population; however, it is no longer a rare disorder in certain populations such as Japan (see Table 7). CAGS also listed the incidence as 51–100 per 100,000 in Arab population; CAGS further mentioned that estimates of the incidence of FMF in specific eastern Mediterranean populations ranged from 1 in 2000 to 1 in 100, depending on the population studied. Thus, the MAF that is seen at 1.55% was acceptable. (iii) rs61757294 (MAF_KWT:15.19% and MAF_1KGP:5.3%) associated with Corticosterone Methyloxidase Type II Deficiency, a rare genetic disorder: ClinVar annotated this variant as pathogenic based on evidence from literature publication and benign based on clinical testing records. The variant was found in patients of Iranian Jewish ancestry. This had DR mode of inheritance – both this variant and another one rs289316 need to be homozygous; thus, the observed high MAF was acceptable; in fact, the variant was a common variant in 1KGP as well. (iv) rs12021720 (MAF_KWT:13.47% and MAF_1KGP:10.9%) associated with Maple syrup urine disease, intermediate, type II (a rare genetic disorder): ClinVar annotated this variant as pathogenic based on literature evidence and as benign with clinical testing as source of annotation. Though the incidence rate world-wide is 1 in 185,000, CAGS reported an incidence rate of 2 in 10,000 in Bahrain - still the higher MAF is not justified. This mutation was seen in one of the three patients from the study^46^ and the patient was a compound heterozygote for a C to G transversion at nucleotide 309 in exon 4 [rs121965001] and a G to A transition at nucleotide 1165 in exon 9 [rs12021720], causing an Ile-to-Met substitution at amino acid 37 and a Gly-to-Ser substitution at amino acid 323, respectively. Thus, the high frequency of MAF at one of the two variants of the compound heterozygotes was acceptable for pathogenic variant; in fact, the variant was a common variant in 1KGP as well. *(b) One variant retained as pathogenic for rare disorder but with a suggestion that they can be “likely benign”:* rs61751507 (MAF_KWT:7.47% and MAF_1KGP:2.7%) associated with Carboxypeptidase N deficiency, which is possibly a complex disorder. ClinVar annotated this variant as pathogenic with evidence from literature publication and benign based on information clinical testing. The study^47^ found this pathogenic variant in just one patient and hence it may be considered as of insufficient evidence. Hence this variant can be considered as “Likely Benign”.

### Missense variants rare within global populations but common within Kuwaiti population

170 SNVs were identified as rare in global populations but common in Kuwaiti exomes; 85 of these were missense variants (Supplementary Table S6). The 85 variants were of two categories: (a) A set of 20 variants harboured in genes annotated for disorders in OMIM: However, these 20 variants were not of any pathogenic value as ClinVar annotated these variants as either ‘benign’ or ‘conflicting interpretation’. Not surprisingly, the REVEL scores in these instances (except in 2 instances – GLDC variant at around 0.8; and the DPYD variant at 0.4) were seen low at ≤0.3. (b) A set of 65 variants harboured in genes NOT annotated for any disorder in OMIM: Association with phenotypes was seen with only one of these 85 variants; the *TTC38* variant rs117135869 (REVEL = 0.621; MAF_KWT: 5.0%; MAF_1KGP: 0.58%) has been recently identified as a novel metabolic quantitative trait loci (mQTLs) in a cohort from Middle Eastern population^48^; this variant was seen in 29 of the Kuwaiti exomes in the heterozygous form.

### Missense variants mapping to drug-binding domains and were of pharmacogenomic relevance

We identified 21 missense SNVs that mapped to a set of 130 drug-binding domains reported in literature^49^ and were annotated in PharmGKB^50^ (Table 4). These 21 variants had impact on the efficacy of drugs used largely for treating common disorders (such as heart failure, hypertension, Chemotherapy, neoplasms, diabetes, nephrosclerosis, rheumatoid arthritis, asthma, pulmonary diseases, schizophrenia, tobacco use disorder, heroin dependence, sickle-cell anemia, and HIV). Furthermore, literature survey revealed that 7 of these 21 pharmacogenomic variants were associated with complex disorders in Arab studies (Table 5).

**Table 4:** 21 missense variants mapping to drug-binding domains and of pharmacogenomic relevance (efficacy, dosage toxicity).

Chr:Position	dnSNP ID; variant change: amino acid change	Gene	KWT_RAF/1KGP_ RAF/GME_RAF; Ratio KWT-1KGP/KWT-GME RAFs	Ref_risk	Affecting Allele(s) for the drug response	Drug response to which is impacted	Disease/Condition
4:3006043	rs1024323; c.425C > T: Ala142Val	*GRK4*	0.3718/0.3732/0.3887; 0.9962/0.9565	C_T	T	atenolol	Hypertension
					CC	metoprolol	Hypertensive Nephrosclerosis
					CC	metoprolol	Hypertensive Nephrosclerosis
17:7579472	rs1042522; c.215C > G: Pro72Arg	*TP53*	0.4425/0.5429/.; 0.8150/.	G_C	CG + GG	cisplatin; paclitaxel	Stomach neoplasms
5:148206440	rs1042713; c.46A > G: Arg16Gly	*ADRB2*	0.5513/0.5244/0.5645; 1.0513/0.98	G_A	AA	terbutaline	Asthma
					AA	salmeterol	Asthma
					A	Beta Blocking Agents	Heart Failure
					G	hydrochlorothiazide	Hypertension
					A	indacaterol	Chronic obstructive pulmonary disease
5:148206473	rs1042714; c.79C > G: Gln27Glu	*ADRB2*	0.2246/ 0.2053/0.2412; 1.094/0.9312	G_C	GG	terbutaline	Asthma
					G	Beta Blocking Agents	Heart Failure
					C	indacaterol	Chronic obstructive pulmonary disease
15:75012985	rs1048943; c.1384A > G: Ile462Val	*CYP1A1*	0.0653/0.1334/0.0619; 0.4895/1.0542	T_C	C	warfarin	warfarin
10:96798749	rs10509681; c.1196A > G: Lys399Arg	*CYP2C8*	0.1198/0.0457/0.1047; 2.6197/1.1438	T_C	C	rosiglitazone	Diabetes
					C	repaglinide	Diabetes
					C	paclitaxel	Cancer(chemotherapy)
7:75615006	rs1057868; c.1508C > T: Ala503Val	*POR*	0.2609/0.2861/0.2808; 0.9120/0.9292	C_T	CT + TT	tacrolimus	Kidney transplantation
17:37879588	rs1136201; c.1963A > G: Ile655Val	*ERBB2*	0.0975/0.1214/0.1255; 0.8032/0.7770	A_G	G	trastuzumab	Breast cancer
10:96827030	rs11572080; c.416G > T: Arg139Lys	*CYP2C8*	0.1237/0.0457/0.1013; 2.7054/1.2211	C_T	T	paclitaxel	Cancer(chemotherapy)
					T	rosiglitazone	Diabetes
					T	repaglinide	Diabetes
					T	paclitaxel	Neoplasms
2:21263900	rs1367117; c.293C > T: Thr98Ile	*APOB*	0.1448/0.1693/0.1727; 0.8553/0.8386	G_A	AG	Irbesartan	Hypertension
					GG	Irbesartan	Hypertension
11:113270828	rs1800497; c.2137G > A: Glu713Lys	*ANKK1*	0.1713/0.3257/0.1908; 0.5259/0.8975	G_A	A	risperidone	Schizophrenia
					A	lithium	Bipolar disorder
					A	antipsychotics	Schizophrenia
					A	Drugs used in nicotine dependence	Tobacco use disorder
					AA + AG	aripiprazole	Schizophrenia
					AA	methadone	Heroin dependence
5:148206885	rs1800888; c.491C > T: Thr164Ile	*ADRB2*	0.0121/0.0040/0.0156; 3.0325/0.7759	C_T	CT	terbutaline	Asthma
					T	Beta Blocking Agents	Heart Failure
3:124456742	rs1801019; c.638G > C: Gly213Ala	*UMPS*	0.1632/0.1859/0.1767; 0.8780/0.9236	G_C	C	fluorouracil	Colonic neoplasms
4:3039150	rs1801058; c.1457T > C: Val486Ala	*GRK4*	0.2293/0.3067/0.292; 0.7476/0.7079	T_C	T	atenolol	Hypertension
					CT	metoprolol	Hypertensive Nephrosclerosis
					CT	metoprolol	Hypertensive Nephrosclerosis
1:237048500	rs1805087; c.2756A > G: Asp919Gly	*MTR*	0.2603/0.2183/0.2021; 1.1929/1.2881	A_G	A	folic acid; hydroxychloroquine; methotrexate; sulfasalazine	Rheumatoid Arthritis
7:55229255	rs2227983; c.1562G > A: Arg521Lys	*EGFR*	0.2759/0.2921/0.2784; 0.9443/0.9907	G_A	A	cetuximab	Colorectal neoplasms
22:42525772	rs28371706; c.320C > T: Thr107Ile	*CYP2D6*	0.0446/0.0591/.; 0.7553/.	G_A	A	codeine	Sickle Cell Anemia
					AA + AG	nevirapine	HIV
4:2990499	rs2960306; c.194G > T: Arg65Leu	*GRK4*	0.3711/0.3125/0.3620; 1.1875/1.0250	G_T	T	atenolol	Hypertension
					GT + TT	metoprolol	Hypertensive Nephrosclerosis
					TT	metoprolol	Hypertensive Nephrosclerosis
19:41522715	rs3211371; c.1459C > T: Arg487Cys	*CYP2B6*	0.0423/0.0535/0.0624; 0.7896/0.6767	C_T	TT	nevirapine	HIV
					TT	methadone	Heroin dependence
19:41512841	rs3745274; c.516G > T: Gln172His	*CYP2B6*	0.2759/0.3157/0.3026; 0.8738/0.9116	G_T	TT	efavirenz	HIV
					T	nevirapine	HIV
					TT	methadone	Heroin dependence
					GT + TT	nevirapine	HIV
					T	nevirapine	HIV
					GT	nevirapine	HIV
					TT	nevirapine	HIV
					TT	nevirapine	HIV
					GT	nevirapine	HIV
					TT	nevirapine	HIV
1:230845794	rs699; c.803T > C: Met268Thr	*AGT*	0.583/0.7051/0.5534; 0.8268/1.0535	A_G	G	Antihypertensives	Hypertension

**Table 5:** Subset of 7 of the identified pharmacogenomic variants (from Table 4) that were also reported in Arab studies as relating to complex disorders.

Pharmaco-genomic variant	*Gene*	KWT_RAF/1kGP_RAF/GME_RAF	Ratio KWT_1KGP/Ratio KWT_GME	Ref_risk allele	Drug to which response is reported	Disorder treated with the drug	Disorder with which Arab studies associate the variant
rs1805087	*MTR*	0.260/0.218/0.202	1.1929/1.2881	A_G	Folic acid; hydroxy-chloroquine; methotrexate; sulfasalazine.	Rheumatoid arthritis	Autism – North Iran^97^. GG seems to be the risk factor. GG seems to be the risk factor. **Complex trait**.
rs1042522	*TP53*	0.443/0.543/-	0.8150/-	G_C	Cisplatin; paclitaxel	Stomach neoplasms	Susceptibility to Breast Cancer in Tunisia^98^. **Complex trait**.
rs1800497	*ANKK1*	0.1713/0.326/0.191	0.5259/0.8975	G_A	Risperidone	Schizophrenia	Risk of Schizophrenia in Egyptians^99^. CC and CT genotypes are the risk factors. Complex trait.
rs1801058	*GRK4*	0.229/0.307/0.292	0.7476/0.7079	T_C	Atenolol	Hypertension	Risk of myocardial infarction among hypertensive subjects in Jordan^100^. **Complex trait**.
rs699	*AGT*	0.583/0.705/0.553	0.8268/1.0535	A_G	Anti-hypertensive	Hypertension	Reduced life-span through genetic susceptibilities to both Essential Hypertension and Myocardial Infarcation in UAE^101^. **Complex trait**.
rs1042713	*ADRB2*	0.551/0.5244/0.565	1.0513/0.98	A_G	Terbutaline	Asthma	Susceptibility to early onset obesity, insulin resistance *etc*. in Saudi Arabia^102^. the subjects who carry Gly16 homozygote genotype are in higher risk. **Complex trait**.
rs1042714	*ADRB2*	0.225/0.205/0.241	1.0941/0.9312	G_C	Terbutaline	Asthma	**Associated with** coronary artery disease/myocardial infarction in Saudi Arabia^103,104^. both the C/G and G/G genotypes are significantly associated with CAD. **Complex trait**.

### SAFD variants and their clinical relevance

For 230 of the 6,186 SAFD variants, ClinVar database provided annotation relating to clinical significance (Supplementary Fig. S5 and Supplementary Table S7). These 230 variants were from 186 unique genes, for 162 of which Inheritance patterns were known; 91 were AR and 63 were AD. 206 of these 230 variants were benign or likely benign. The disorders related to the genes harbouring the benign variants were often single-gene disorders and familial, hereditary and congenital. The 24 non-benign variants (Table 6) were from 21 unique genes associated with 20 unique disorders.

**Table 6:** List of 24 SAFD variants annotated for clinical significance in ClinVar and OMIM.

Chr: Position; dbSNP_ID_Ref_Alt; Gene	KUWAITI		1KGP		gnomAD		Inheritance mode	Disease name; PMIM; number of OMIM listed genes; Is the disorder seen in Arab population?^@^
	KWT_MAF	Max_Pop (MAF)	1KGP_MAF	Max_Pop (MAF)	gnomAD_MAF	Max_Pop (MAF)		
**A**. **2 “Pathogenic” variants as per ClinVar annotation**								
8:143994266; rs61757294_ A_G c.1157T > C; p.Val386Ala *CYP11B2*	0.1519 This has DR mode of inheritance – both this and another variant rs289316 need to be homozygous; hence this higher MAF is alright though the variant is “pathogenic”.	KWS (0.1698)	0.0531	EUR (0.1093)	0.0846	ASJ (0.1163)	DR (both rs61757294 and rs28931609 need to be homozygous)	Corticosterone methyloxidase type 2 deficiency. PMIM: 610600; Single gene. Rare Genetic disorder. Yes, disorder seen in Arab country.
10:101829514 rs61751507_ C_T c.533G>A; p.Gly178Asp *CPN1*	0.0747 (high MAF for “pathogenic” variant). ClinVar annotates this variant as pathogenic based on one literature publication which reports this variant in just one patient. Thus, it is possible that the significance is of insufficient evidence and that the variant may become “likely benign”.	KWS (0.0981)	0.0266	AMR (0.0634)	0.0423	AMR (0.0668)	AR	Carboxypeptidase N deficiency PMIM: 212070 Single gene. Familial Carboxypeptidase N deficiency is a rare disorder.
**B**. **4 Drug response variants as per ClinVar annotation**								
16:31105945 rs61742245_ C_A c.106G>T; p.Asp36Tyr *VKORC1*	0.0104	KWB (0.0294)	0.0004	EUR (0.0010)	0.0024	ASJ (0.0384)	AD	Warfarin resistance PMIM: 122700 Multiple genes and rare disorder. Appears already in Table 3 as drug response.
8:18257854 rs1801280_ T_C c.208G>A; p.Asp70Asn *NAT2*	0.3927	KWP (0.3879)	0.2927	EUR (0.4493)	0.3821	FIN (0.4668)	AR Forms part of NAT2*5B haplotype	Slow acetylator due to N-acetyltransferase enzyme variant. Toxicity to the drugs of cisplatin or cyclophosphamide. PMIM: 243400 Yes, disorder seen in Arab country.
10:96702047 rs1799853_ C_T c.430C>T; p.Arg144Cys *CYP2C9*	0.1181	KWS (0.1262)	0.0479	EUR (0.1243)	0.0926	ASJ (0.1357)	AD	Warfarin sensitivity PMIM: 122700 **Multiple genes**.
19:15990431 rs2108622_ C_T c.1297G>A; p.Val433Met *CYP4F2*	0.4102	KWS (0.4541)	0.2368	SAS (0.4131)	0.2735	SAS (0.3978)	Na	Acenocoumarin response – Dosage. Warfarin sensitivity. PMIM: 122700 **Multiple genes**.
**C**. **14 Risk factor variants**								
**C**. **1**. **7 Risk factor variants as per ClinVar annotation for complex disorders**								
2:138759649 rs11558538_ C_T c.314C>T; p.Thr105Ile *HNMT*	0.1259	KWS (0.1495)	0.0595	SAS (0.1053)	0.1008	FIN (0.1601)	AD	Asthma, susceptibility to; PMIM: 600807 Multiple genes. Yes, disorder seen in Arab country.
4:100268190 rs283413_ C_A^# $^ c.232G>T; p.Gly78Arg *ADH1C*	0.0653	KWP (0.1071)	0.0072	SAS (0.0174)	0.0157	ASJ (0.0633)	IC,Mu	Parkinson’s disease, susceptibility to; PMIM: 168600 **Multiple genes**.
5:95751785 rs6232_ T_C c.661A>G; p.Asn221Asp *PCSK1*	0.0594	KWB (0.0909)	0.0210	SAS (0.0501)	0.0390	SAS (0.0659)	?	Obesity, susceptibility to, Body mass index quantitative trait locus 12 PMIM: 612362. OMIM lists single gene but in reality, BMI is associated with multiple genes.
10:64415184 rs7076156_ G_A^# &^ c.1130-972G>A; p.Ala62Pro *ZNF365*	0.3351	KWS (0.4450)	0.1288	EUR (0.2734)	0.2044	ASJ (0.2795)	Na	Uric acid nephrolithiasis, susceptibility to; PMIM: 605990. OMIM lists single gene but this is a multifactorial disorder.
14:104165753 rs861539_ G_A c.1849-1239G>A; p.Thr241Met *XRCC3*	0.3864	KWS (0.4450)	0.2169	EUR (0.3936)	0.2904	ASJ (0.4015)	AD	Cutaneous malignant melanoma 6, susceptibility to PMIM: 613972. OMIM lists single gene but this is a multifactorial disorder.
17:5485367 rs12150220_ A_T c.464T>A; p.Leu155His *NLRP1*	0.4377	KWP (0.4643)	0.1921	EUR (0.4443)	0.3674	ASJ (0.4744)	AR	Vitiligo-associated multiple autoimmune disease susceptibility 1; PMIM: 606579. OMIM lists single gene but this is a multifactorial disorder.
17:48437456 rs6504649_ C_G c.2402C>G; p.Thr801Arg *XYLT2*	0.4414	KWB (0.4706)	0.2510	EUR (0.4006)	0.3312	ASJ (0.4536)	AR	Pseudoxanthoma elasticum, modifier of severity; PMIM: 264800. OMIM lists multiple genes: *XYLT1*, *XYLT2*, *ABCC6*. Yes, disorder seen in Arab country.
**C**. **2**. **7 Risk factor variants as per our inference but annotated as “pathogenic” in ClinVar annotation**								
9:116153891 rs1800435_ C_G c.177G>C; p.Lys59Asn *ALAD*	0.1241	KWB (0.1912)	0.0635	SAS (0.1585)	0.0830	ASJ (0.2207)	AR	Aminolevulinate dehydratase, ALAD*1/ALAD*2 allele at this position associated with susceptibility to lead poisoning. PMIM: 612740. Lead poisoning is becoming a common disease. Single gene.
10:54531242 rs5030737_ G_A c.154C>T; p.Arg52Cys *MBL2*	0.0790	KWP (0.0902)	0.0272	EUR (0.0596)	0.0558	ASJ (0.1032)	AD	Mannose-binding lectin deficiency. PMIM: 614372 Single gene. Complex trait
15:100230557 rs121918530_ A_G c.782A>G; p.Asn261Ser *MEF2A*	0.0103	KWB (0.0294)	0.0004	EUR (0.0020)	0.0008	NFE (0.0015)	AD	Coronary artery disease/myocardial infection PMIM: 608320 Single gene. Complex trait
17:12899902 rs5030739_ C_T c.1621G>A; p.Ala541Thr *ELAC2*	0.0842	KWS (0.1055)	0.0232	SAS (0.0501)	0.0349	ASJ (0.0510)	AR or AD? Has to be seen in compound heterozygous state with another variant rs4792311.	Susceptibility to Prostate cancer, hereditary, 2′ PMIM: 614731 Single gene. Complex trait
17:12915009 rs4792311_ G_A c.650C>T; p.Ser217Leu *ELAC2*	0.3552	KWP (0.3611)	0.2145	EUR (0.3151)	0.2742	ASJ (0.3699)	AR or AD? Has to be seen in compound heterozygous state with the previous variant of rs5030739.	Susceptibility to Prostate cancer, hereditary, 2′; PMIM: 614731 Single gene. Complex trait
17:79767715 rs1801483_ G_A c.118G>A; p.Gly40Ser *GCGR*	0.0378	KWS (0.0505)	0.0042	EUR (0.0149)	0.0075	ASJ (0.0120)	AD	Diabetes mellitus type 2, non-insulin dependent; PMIM: 125853; Multiple genes. Multifactorial complex disorder. Yes, disorder seen in Arab country.
18:55373793 rs34719006_ C_T c.208G>A; p.Asp70Asn *ATP8B1*	0.0258	KWS (0.0413)	0.0018	AFR (0.0045)	0.0031	ASJ (0.0096)	AD	Cholestasis of pregnancy; PMIM: 147480 Single gene. However, it is the most common liver disease unique to pregnancy. Complex trait.
**D**. **2 variants associated with complex traits by GWAS studies followed by clinical testing as per ClinVar annotation**								
2:27730940 rs1260326_ C_T^#^ c.1337T>C; p.Leu446Pro *GCKR*	0.3945	KWS (0.4352)	0.2933	EAS (0.4812)	0.3667	ASJ (0.5344)	Not available	Fasting plasma glucose level quantitative trait locus 5 PMIM: 613463 Single gene (but, FPG levels are associated with multiple genes).
11:68846399 rs35264875_ A_T c.1450A>T; p.Met484Leu *TPCN2*	0.1832	KWP (0.2260)	0.0996	SAS (0.2055)	0.1593	FIN (0.2980)	Not available	Skin/hair/eye pigmentation, variation in, SHEP10 PMIM: 612267; OMIM lists single gene (but, the variations in skin/hair/eye pigmentation variations are associated with multiple genes).
**E**. **2 Protective variants as per ClinVar annotation**								
4:100260789rs698_ T_C c.1048A > G; p.Ile350Val *ADH1C* This variant is in LD with the variant R271Q (corresponding to the variant listed in the next row rs1693482) that is responsible for the differences in enzymatic differences	0.3137	KWS (0.3515)	0.2143	EUR (0.4046)	0.3470	FIN (0.5169)	Ic, Mu	Alcohol dependence, protection against; PMIM: 103780. Multiple genes.
4:100263965 rs1693482_ C_T c.815G>A; p.Arg272Gln *ADH1C*	0.3296	KWS (0.3830)	0.2143	EUR (0.4046)	0.3462	FIN (0.5167)	Ic, Mu	Alcohol dependence, protection against; PMIM: 103780. Multiple genes.

^@^Disorders relating to the following variants rs61757294 (*CYP11B2*, Pathogenic, DR, Corticosterone methyloxidase type 2 deficiency). rs1801483 (*GCGR*, reannotated as risk factor, AD form T2D), rs11558538 (*HNMT*, Risk factor, AD, susceptibility to asthma), rs1801280 (*NAT2*, drug response, AR, Slow acetylator due to N-acetyltransferase enzyme variant) and rs6504649 (*XYLT2*, Risk Factor, AR, Pseudoxanthoma elasticum, modifier of severity) are seen annotated in CAGS as observed in many Arab countries.

Seven of these 24 non-benign SAFD variants were seen annotated in ClinVar as “Pathogenic”; however, either the associated disorder was common/complex or more prevalent in the study population or the patient carrying the variant was annotated in OMIM as susceptible to the disorder (which is usually a common disorder). Going by the practice that “pathogenic” variants are related to Mendelian disorders, we considered the variants associated with common disorders as risk factors. (i) rs1800435_G > C (MAF_KWT:12.41%; MAF_1KGP:6.4%) associated with “Aminolevulinate dehydratase, alad*1/alad*2 polymorphism susceptibility to lead poisoning ALAD porphyria”. ClinVar annotated this as pathogenic based on literature evidence and likely benign based on clinical testing. It increases the risk for lead poisoning. ALAD porphyria is a very rare genetic metabolic disease; however, quoting from the CDC report on lead poisoning – “There are approximately half a million U.S. children ages 1–5 with blood lead levels above 5 micrograms per deciliter (µg/dL), the reference level at which CDC recommends public health actions be initiated”, lead poisoning is no longer a rare disorder. Susceptibility is the keyword, and we reannotated this variant as risk factor. (ii) rs5030737 (MAF_KWT:7.90%; MAF_1KGP:2.8%) associated with Mannose-binding lectin deficiency, which is a complex trait. ClinVar annotated the variant as pathogenic based on literature reference; since we associate “pathogenic” to Mendelian disorders, we reannotated this variant as risk factor. (iii) rs121918530 (MAF_KWT:1.03%; MAF_1KGP:0.04%)associated with coronary artery disease/myocardial infarction, which is a *complex* multifactorial *disorder*. ClinVar annotated this variant as pathogenic based on literature evidence and likely benign based on clinical testing. since we associate “pathogenic” to Mendelian disorders, we reannotated this variant as risk factor. (iv) rs5030739 (MAF_KWT:8.42%; MAF_1KGP:2.32%) associated with “Prostate cancer hereditary 2, susceptibility to” (Complex trait). ClinVar annotated this variant as pathogenic based on literature evidence and benign based on clinical testing. The cited literature suggested increased risk of prostate cancer; ‘susceptibility to’ was the keyword. Thus, this variant was considered as risk factor. It was also the case that this variant has to appear in compound heterozygosity with the next listed variant of rs4792311. We reannotated this variant as risk factor. (v) rs4792311 (MAF_KWT:35.52%; MAF_1KGP:21.5%) associated with “Prostate cancer hereditary 2, susceptibility to” (Complex trait). ClinVar annotated this variant as pathogenic based on literature evidence and benign based on clinical testing. The cited literature suggested increased risk of prostate cancer; ‘susceptibility to’ is the keyword. Thus, this variant was considered as risk factor. It was also the case that this variant had to appear in compound heterozygosity with the previous listed variant of rs5030739. We reannotated this variant as risk factor. (vi) rs1801483 (MAF_KWT:3.78%; MAF_1KGP:0.42%) associated with Diabetes mellitus type 2, non-insulin dependent (a multifactorial complex disorder). ClinVar annotated the variant as pathogenic (based on literature evidence). Considering that we associate “pathogenic” only with Mendelian disorder, we reannotated the variant as risk factor. (vii) rs34719006 (MAF_KWT:2.58%; MAF_1KGP:0.18%) associated with Cholestasis of pregnancy, which is a most common liver disease unique to pregnancy. (Complex trait). ClinVar annotated this variant as pathogenic based on evidence from literature study and annotated as with conflicting evidence between likely benign (clinical testing), uncertain significance (Clinical testing). Considering that we associate “pathogenic” only with Mendelian, we reannotated this variant as risk factor.

This set of 24 SAFD variants with clinical significance was distributed onto (a) A set of 2 pathogenic variants (rs61757294 and rs61751507) with AR mode inheritance; the MAF of these two variants in Kuwaiti exomes were uncharacteristic of pathogenic variants (see above for more details); (b) A set of 4 drug response variants one of which was AR); (c) A set of 14 risk variants and 2 protective variants for complex traits (3 were AR); and (d) A set of two variants associated with phenotype traits through GWAS studies. Five of the disorders associated with the SAFD variants were seen annotated in CAGS as observed in Arab countries (see Table 6).

### Assessing the Loss-of-Function SAFD Variants for clinical significance

We had identified 26 LoF SAFD variants (Supplementary Table S4); as many as 15 of these were stop-gain, seven were start-loss and the remaining four were splice site mutations. None of these 26 SAFD LoF variants was seen annotated for disorder in OMIM; however, the GWAS Catalog^51^ listed one of these variants namely rs2228015-C from *CCR7* gene as associated with the complex phenotype trait of lymphocyte counts (at genome-wide significant p-value of 6E-09).

### CAGS disorders for which the OMIM-listed causal variants were seen in Kuwaiti exomes

We further examined the CAGS database for disorders observed in Kuwait at any incidence rate and for disorders seen in any of the Arab countries at incidence rates of ≥11 per 100,000. CAGS database provided the Phenotype MIM number using which we retrieved the OMIM-reported causal variants and checked for their occurrences in Kuwaiti exomes. For 25 disorders, the OMIM-reported variants were seen in Kuwaiti exomes (Table 7); eight of these 25 disorders had already been seen in the analysis for functional variants. Except in one instance (rs1800858), all the variants were missense. 13 of these variants were “pathogenic” and the remaining 12 were “risk factor” variants. 18 of these disorders were observed in Kuwait and the remaining in other Arab countries.

**Table 7:** Arab disorders (annotated in CAGS) for which the OMIM-listed risk variants were seen in Kuwaiti exomes.

SNPs from Kuwaiti exomes seen annotated in OMIM against the CAGS disorder; Ref > risk alleles	Disorder	ClinVar annotation for the disorder (CAGS OMIM Id)	Incidence Rates	Inheritance	Carrier Frequencies (rr/rR/RR)	RAF(KWT/1KGP/GME)	Ratios (KWT-1KGP /KWT-GME)
**A. 7 Pathogenic variants for rare disorders**							
rs12021720 C > T p.Gly384Ser *DBT*	Maple syrup urine disease, intermediate, type ii (PMIM:248600). **It is a rare genetic disorder**. OMIM lists single gene for the Type II MSUD	Pathogenic for the subtype.		AR	5/67/218	0.1347/0.1082/0.12 The literature publication, cited in OMIM, reporting this variant as pathogenic, found it in compound heterozygosity with another variant [rs121965001] in the patient. Thus, the high MAF at one of the two variants of the compound heterozygotes is perhaps acceptable for pathogenic variant.	0.97/0.98
rs79204362 C > T p.Arg368His *CYP1B1*	Glaucoma, early-onset, digenic (PMIM: 231300). OMIM lists single gene. This is a **rare disorder** but reaches high prevalence in Saudi Arabian populations	Pathogenic	11–50	AR	0/6/284	0.0103/0.0042/0.0137 Incidence rate in Middle East is 51–100 per 100,000 (i.e. 5 in 10,000); CTGA reports as high as 1 in 2,500 in Saudi Arabian population. Accordingly, MAF is seen marginally higher at 1.03%	2.47/0.76
rs61732874 C > A p.Ala744Ser *MEFV*	Familial Mediterranean fever (PMIM: 249100). OMIM lists single gene. However, this can be a common disorder in certain populations.	Pathogenic/Likely Pathogenic	51–100	AR	0/9/278	0.0155/0.0018/0.0126 FMF is a rare disorder in Europe; however, it is no longer a rare disorder in populations such as Japan^105^. CAGS lists an incidence of 51–100 per 100,000 in Arab population; estimates of the incidence in specific eastern Mediterranean populations range from 1 in 2000 to 1 in 100, depending on the population studied. Accordingly, MAF is seen marginally higher at 1.55%.	8.60/1.23
rs121908530 G > A p.Gly156Arg *AGXT*	Hyperoxaluria, primary, type i (PMIM: 259900). Rare disorder OMIM lists single gene.	Pathogenic			0/1/290	0.0017/./.	./.
rs118204113 G > A p.Ala252Thr *HMBS*	Porphyria, acute intermittent (PMIM:176000). Rare form of porphyria. OMIM lists single gene.	Pathogenic		AD	0/1/289	0.0017/./0.0005	0./3.41
rs587776954 A > G p.Met1Val; LoF *C12ORF57*	Temtamy syndrome (PMIM:218340). GARD lists this as a rare disorder. OMIM lists single gene.	Pathogenic		AR	0/2/288	0.0034/./0.0030	0./1.14
rs4149584 C > T p.Arg121Gln *TNFRSF1A*	Periodic fever, familial, autosomal dominant (PMIM:142680). Rare disorder OMIM lists single gene.	Pathogenic		AR	0/4/287	0.0069/0.0069/0.0121	1.00/0.57
**B.10 Risk Factor/association variants**							
**B**. **1**. **1 Risk factors for supposedly rare disorders**							
rs61747728 C > T p.Arg229Gln *NPHS2*	Nephrotic syndrome, type 2, susceptibility to (PMIM: 600995). OMIM lists single gene. Rare disorder.	Risk factor Rare disorder; however, Zaki *et al*.^106^ concluded that the incidence of this disease seemed to be higher among Arab children (as seen in Kuwaiti hospitals over 5 years period) than in Western countries.	11–50	AR	0/8/281	0.0138/0.0146/0.0121	0.94/1.14
**B**. **2**. **9 Risk factors/associations for common disorders**							
rs3135506 G > C p.Ser19Trp *APOA5*	Hypertriglyceridemia Familial, susceptibility to (PMIM: 145750). OMIM lists two genes. This is an inherited common disorder.	Risk factor for Common disorder		AD	1/22/267	0.0412/0.0557/0.0706	0.74/0.58
rs2476601 G > A p.Arg620Trp *PTPN22*	Diabetes mellitus Type 1, insulin-dependent, susceptibility to (PMIM:222100). OMIM lists multiple genes. Complex disorder And associated with susceptibility to Systemic lupus erythematosus as well (PMIM:152700).	Risk factor. Also risk factor for many other disorders such as SLE		AR	0/5/280	0.0292/ 0.0274/0.0131	1.00/0.98
rs1799945 C > G p.His63Asp *HFE*	Hemochromatosis, type 1 - microvascular complications of diabetes, susceptibility to, 7, included (PMIM:235200). OMIM lists two genes. Common disorder.	Risk factor	>100	AR	4/63/224	0.122/0.0731/0.1198	1.67/1.02
rs17158558 C > T p.Arg982Cys *RET*	Hirschsprung disease, susceptibility to, 1 (PMIM 142623). OMIM lists single gene. Complex disorder.	Risk factor		AD	0/15/273	0.0258/0.0220/0.0459	1.17/0.56
rs121918219 G > A p.Arg274Gln *VANGL1*	Neural tube defects, susceptibility to (PMIM: 182940). OMIM lists two genes. The average incidence of NTDs is 1/1000 births, with a marked geographic variation^107^.	Risk factor.	>100	AD	0/1/289	0.0017/./0.0010	./1.71
rs1800858 G > A p.Ala45Ala Synonymous *RET*	Hirschsprung disease, susceptibility to, 1; HSCR1 (PMIM: 142623). CAGS reports an incidence rate of 1 per 5000 live births. OMIM lists single gene.	Risk factor		AD	25/128/138	0.3058 /0.2464/0.2593	1.24/1.1793
rs1801131 T > G p.Glu470Ala *MTHFR*	Schizophrenia; sczd (PMIM: 181500) OMIM lists multiple genes. Complex trait.	Risk factor		AD	31/128/132	0.3265/0.3264/0.3353	1.00/0.97
rs2282440 G > A p.Thr329Ile *SDC3*	Obesity (PMIM: 601665). OMIM lists multiple genes. Complex trait.	Association		AD	0/11/280	0.0189/0.0189/0.0282	1.00/0.67
rs77775126 C > T p.Thr373Ile *RP1*	Retinitis pigmentosa 1; RP1 (PMIM: 180100). OMIM lists single gene. It is a common disorder. The disorder is seen in CAGS with prevalence as approximately 1/3,000 to 1/5,000.	Risk Factor by way of inference – since we associate “pathogenic” only with Mendelian disorder.		AR	0/11/280	0.0189/0.0189/0.0181	1.00/1.04
**C**. **6 Pathogenic variants that already appeared in the list of RARE & DELETERIOUS variants (see** Table 3)							
rs104893836 T > C p.Gln106Arg *GNRHR*	Hypogonadotropic hypogonadism 7 without anosmia (PMIM:146110). OMIM lists single gene. MEDSCAPE lists Hypogonadotropic hypogonadism as rare genetic disorder.	Pathogenic		AR	0/1/290	0.0017/0.0012/0.0055	1.43/0.31
rs58331765 C > T p.Val931Met *ABCA4*	Stargardt disease 1 (PMIM:248200). OMIM lists two genes. Rare disorder.	Likely pathogenic/Pathogenic.		AR	0/2/288	0.0034/0.0028/0.0005	1.23/6.82
rs61753185 G > A p.Arg77Gln *TYR*	Tyrosinase-negative oculocutaneous albinism, Type IA (PMIM:203100). OMIM lists single gene. Rare disorder.	Pathogenic for Tyrosinase-negative oculocutaneous albinism		AR	0/1/290	0.0017/0.0004/0.0005	4.30/3.41
rs61754375 G > A p.Arg299His *TYR*	Tyrosinase-negative oculocutaneous albinism, Type IA (PMIM: 203100). OMIM lists single gene. Rare disorder.	Pathogenic for Tyrosinase-negative oculocutaneous albinism		AR	0/1/286	0.0017/0.0002/.	8.60/.
rs34424986 G > A p.Arg275Trp *PARK2*	Parkinson disease 2, autosomal recessive juvenile; *PARK2* (PMIM:600116). OMIM lists single gene. Rare disorders.	Pathogenic			0/2/289	0.0034/0.0034/.	1.00/.
rs56208331 G > A p.Asp426Asn *GATA4*	Tetralogy of fallot; (PMIM:187500). OMIM lists single gene. Rare disorder.	Pathogenic		AD	0/2/289	0.0034/0.0034/0.0025	1.00/1.36
**D. 2 risk factor variants that already appeared in Tables** 3 **and** 6							
rs121918530 A > G p.Asn261Ser *MEF2A*	Coronary artery disease/myocardial infarction (PMIM: 608320). OMIM lists single gene. However, it is a complex disorder.	Risk factor by way of inference		AD	0/6/284	0.0103/0.0004/0.0020	25.82/5.12
rs11558538 C > T p.Thr105Ile *HNMT*	Asthma, susceptibility to (PMIM: 600807). Common disorder OMIM lists multiple genes.	Risk factor		AD	7/55/212	0.1186/0.0595/0.0968	1.99/1.23

### Scrutinization of the identified variants against Arab mutations reported in Arab studies

Analyses performed so far in the study indicated that disorders relating to 20 instances of rare & deleterious variants, 16 of which were pathogenic variants for rare disorders and 4 were risk factor variants for complex disorders (see Table 3), 7 instances of pharmacogenomic variants that were associated with complex disorders in Arab studies (see Table 5), 5 instances of SAFD variants (see Table 6), and 17 additional instances from the analysis of CAGS disorders were seen in Arab population (see Table 7). During the analysis, we also found in Kuwaiti exomes two recessive mutations (namely rs1801133 & rs1801131 from MTHFR – see Table 8) associated with recessive early onset of susceptibility to Type 2 diabetes in Arab population. We set upon to identify which of these variants were also reported in Arab studies for the corresponding disorder. Upon performing literature survey and manual examination of the bibliography data presented in CAGS database, these variants could be classified onto the following categories (Table 8): (a) 16 Instances where the OMIM-listed variants identified in Kuwaiti exomes were also reported as Arab mutations in Arab studies. 9 of these were pathogenic variants for rare disorder; 1 was drug response; and 6 were risk factors for complex disorders. (b) 7 Instances where the identified pharmacogenomic variants in Kuwaiti exomes were also observed as associated with disorders in Arab studies. These were drug response variants to complex disorders. (c) 12 instances of disorders where the genetic basis at SNV level had not been reported in Arab studies. 7 of these were pathogenic variants for rare disorders and 5 were risk factors for complex disorders. (d) 10 Instances where the Arab studies reported variants different from the OMIM-listed variants observed in Kuwaiti exomes; however, the Arab reported variants were from the same gene. The Arab variants were generally seen in OMIM but not in our exomes. Eight of these variants were pathogenic for rare disorders and two were risk factors for complex disorders. (e) 7 Instances of variants (from 4 disorders) where the Arab studies reported variants from genes different from those of the OMIM-listed variants observed in Kuwaiti exomes. In general, the different gene and the different mutations were listed in OMIM for the disorder but were not seen in Kuwaiti exomes. All the 7 variants were risk factors for complex disorders.

**Table 8:** Evaluation of the identified variants for observation as Arab mutations in Arab studies.

dbSNP ID; Ref_risk alleles; gene	DISORDER; (PMIM); Number of OMIM-listed genes for the disorder; Reference for the Arab study.	Clin. Signific.; CAGS inciden. (per 100,000)	Inheritance mode of the disorder	CARRIER FREQ (rr/rR/RR)	RAF (KWT_RAF/1KGP_RAF/GME_RAF	RATIO (KWT-1KGP/KWT-GME)
A. **16 Instances wherein the identified functional variants match with the variants reported in arab studies**.						
**4 Rare & deleterious variants – Pathogenic and rare disorder**.						
rs137853054 (G_A) *PARK2*	Parkinson disease, juvenile, type 2; (PMIM:600116). GARD lists this as rare disorder. OMIM lists single gene^92^;	Pathogenic and rare disorder	AR	0/2/288	0.0034/0.0002/0.0025	17/1.3503
rs61754375 (G_A) *TYR*	Tyrosinase-negative oculocutaneous albinism, Type IA; (PMIM:203100) – OMIM lists single gene^108^;	Pathogenic and rare disorder	AR	0/1/286	0.0017/0.0002/.	8.5/.
rs121964924 (A_G) *DPYS*	Dihydropyrimidinase deficiency; (PMIM:222748) – OMIM lists single gene^109^;	Pathogenic and rare disorder	AR	0/1/289	0.0017/0.0002/0.0020	8.5/0.8424
rs58331765 (C_T) *ABCA4*	Stargardt disease 1; (PMIM:248200) – OMIM lists two genes^93^;	Pathogenic and rare disorder	AR	0/2/288	0.0034/0.0028/0.0005	1.2143/6.7460
**2 Safd variants**						
rs61757294 (A_G) *CYP11B2*	Corticosterone methyloxidase type 2 deficiency. (PMIM: 610600)^110^. this cited study is on Iranian-Jewish origin patients.	Pathogenic and rare disorder	DR; this and rs28931609 to be homozygous.	??	0.1519/0.0531/? For variants of DR mode of inheritance higher MAF is alright though it is a pathogenic variant.	2.8606/?
rs1801280 (T_C) *NAT2*	Slow acetylator due to N-acetyltransferase enzyme variant; (PMIM:243400) – OMIM lists single gene^111^;	Drug Response	AR	41/134/100	0.3927/0.2927/0.4340	1.3416/0.9047
**8 Cags arab disorders**						
rs79204362 (C_T) *CYP1B1*	Early onset of Glaucoma, digenic; (PMIM: 231300) – OMIM lists single gene^112^; This is a **rare disorder** but reaches high prevalence in Saudi Arabian populations.	Pathogenic and rare disorder; 11–50	AR	0/6/284	0.010/0.004/0.013 CTGA reports 1 in 2,500 among Saudi Arabians. MAF is only marginally higher at 1.03%.	2.4587/0.7553
rs61732874 (C_A) *MEFV*	Familial Mediterranean Fever (FMF) Recessive; (PMIM: 249100) – OMIM lists single gene^113^;	Pathogenic; rare disorder 51–100	AR	0/9/278	0.015/0.001/0.012 FMF is a rare disorder in Europe; it is no longer rare in certain populations^105^. CAGS’ incidence is 51–100; varies 1/2000 to 1/100 in eastern Mediterranean populations. Thus, MAF of 1.55% is acceptable for pathogenic variant.	8.6026/1.2281
rs121908530 (G_A) *AGXT*	Type 1 primary Hyperoxaluria (PMIM: 259900) – OMIM lists single gene^114^;	Pathogenic; rare disorder	?	0/1/290	0.001718/./.	./.
rs587776954 (A_G) *C12orf57*	Temtamy syndrome (PMIM:218340) – OMIM lists single gene^115^;	Pathogenic; rare disorder	AR	0/2/288	0.003436/./0.003021	./1.1374
rs61747728 (C_T) *NPHS2*	Susceptibility to Nephrotic syndrome, type 2 (PMIM: 600995) – OMIM lists single gene^116^;	Risk factor for rare disorder. 11–50	AR	0/8/281	0.01375/0.0145767/0.012085	0.9433/1.1377
rs1799945 (C_G) *HFE*	Type 1 Hemochromatosis - microvascular complications of diabetes, susceptibility to, 7, included (PMIM:235200). OMIM lists two genes^117–119^;	Risk factor for common disorder; >100	AR	4/63/224	0.122/0.073/0.119	1.6693/1.0180
rs1801131 (T_G) *MTHFR*	Schizophrenia; sczd (PMIM:181500) – OMIM lists several genes^120^;	Risk Factor; complex disorder; >100	AD	31/128/132	0.3265/0.3264/0.3353	1.0001/0.9736
rs77775126 (C_T) *RP1*	Retinitis pigmentosa 1 (PMIM: 180100) – OMIM lists single gene^121,122^.	Risk factor by inference for common disorder; 11–50	AR	0/11/280	0.0189/0.0189/0.0181	0.9999/1.0426
**2 Variants that came up during the examination for arab study variants**						
rs1801133 (G_A) *MTHFR*	Susceptibility to T2DM in Lebanese^123,124^.	Risk Factor; complex disorder	AR	12/81/197	0.1810/0.2454/0.2557	0.7376/0.7077
rs1801131 (T_G) *MTHFR*	Protective effect: T2DM in Israel Jews and South Indians^123,125^.	Risk Factor; complex disorder	AD	31/128/132	0.3265/0.2494/0.3353	1.3091/0.9737
**B**. **7 Instances wherein the identified pharmacogenomic variants are implicated in arab disorders through arab studies**. **in all the instances**, **multiple genes are involved in the disorder**.						
rs1042713 (A_G) ADRB2	PharmGKB: drug response to asthma. OMIM: susceptibility to asthma, nocturnal PMIM:600807). **Arab study:** susceptibility to early onset obesity and insulin resistance^96,102^.	Drug response; complex disorder	AD	82/126/55	0.5513/0.5244/0.5645 A->G (Arg16Gly) is causative; hence the frequencies of the risk allele G rather than the minor allele are listed.	1.0513/0.98
rs1042714 ADRB2	PharmGKB: drug response to asthma. OMIM: susceptibility to obesity and to childhood asthma. PMIM: 601665. **Arab study:** for Coronary artery disease/myocardial infarction^103,104^.	Drug response; complex disorder	AR	19/90/176	0.2246/0.2053/0.2412	1.094/0.9312
rs1805087 (A_G) *MTR*	PharmGKB: drug response to Rheumatoid arthritis. OMIM: does not list. **Arab study:** for Autism – North Iran^97^.	Drug response; complex disorder	GG is the risk factor.	23/105/162	0.2603/0.2183/0.2021	0.2603/0.2021
rs1042522 (G_C) *TP53*	PharmGKB: drug response to stomach neoplasm. OMIM: smoking related accelerated decline in lung function PMIM:608852; **Arab study:** in susceptibility to Breast Cancer in Tunisia^98^.	Drug response; complex disorder	??	57/140/90	0.4425/0.5429/.	0.815/.
rs1800497 (G_A) *ANKK1*	PharmGKB: drug response to Schizophrenia. OMIM: Taq1A polymorphism associated with neuropsychiatric disorders. **Arab study:** risk factor for Schizophrenia (PMIM:181500) in Egyptians^99^.	Drug response; complex disorder	AD?	7/85/197	0.1713/0.3257/0.1908	0.5259/0.8975
rs1801058 (T_C) *GRK4*	PharmGKB: drug response to Hypertension. OMIM: does not list. **Arab study:** risk factor for myocardial infarction among hypertensive subjects in Jordan^100^.	Drug response; complex disorder	AD?	16/101/173	0.2293/0.3067/0.292	0.7476/0.7079
rs699 (A_G) *AGT*	PharmGKB: drug response to Hypertension. OMIM: susceptibility to Essential hypertension (PMIM:145500). Arab study: Reduced life-span through genetic susceptibilities to Hypertension and Myocardial Infarction in UAE^101^.	Drug response; complex disorder	Mu?	91/127/47	0.583/0.7051/0.5534	0.8268/1.0535
**C**. **12 Instances wherein the genetic basis for the disorder has not been reported in arab studies**.						
**4 Rare & deleterious variants**						
rs28940872 (C_T) ACADS	Scad deficiency (PMIM:606885). Single gene listed in OMIM.	Pathogenic; rare disorder;	AR	0/1/289	0.0017/0.0002/0.0015	8.5/1.1251
rs28941785 (C_T) CTH	Cystathioninuria (PMIM:219500). Single gene listed in OMIM.	Pathogenic; rare disorder;	AR.	0/2/281	0.0035/0.0026/0.0065	1.3461/0.5347
rs28940885 (C_T) GALE	Galactose Epimerase Deficiency (PMIM:230350). Single gene listed in OMIM.	Pathogenic; rare disorder;	AR	0/1/290	0.0017/0.002/0.0025	0.85/0.6751
rs114817817 (C_T) SRGAP1	Susceptibility to Thyroid cancer, nonmedullary 2, (PMIM:188470). OMIM lists three genes for this disorder.	Risk Factor; common disorder	AD; (AR,AD,MF)	0/1/285	0.0017/0.0006/0.0070	2.8333/0.2415
**1 Safd variants**						
rs6504649 (C_G) THR801ARG *XYLT2*	Pseudoxanthoma elasticum, modifier of severity. (PMIM:264800). OMIM lists three genes.	Risk Factor; common disorder	AR	60/136/94	0.4414/0.251/0.3962	1.7586/1.1139
**8 7 Cags arab variants**						
rs118204113 (G_A) HMBS	Acute intermittent porphyria (PMIM:176000). Single gene listed in OMIM.	Pathogenic; rare disorder	AD	0/1/289	0.001718/./0.000504	./3.4087
rs4149584 (C_T) TNFRSF1A	Familial periodic fever, autosomal dominant. (PMIM:142680). Single gene listed in OMIM.	Pathogenic; rare disorder; 51–100	AR	0/4/287	0.0069/0.0069/0.0121	1.0000/0.5687
rs56208331 (G_A) GATA4	Tetralogy of fallot (PMIM:187500). Single gene listed in OMIM.	Pathogenic; rare disorder; 51–100	AD	0/2/289	0.0034/0.0034/0.0025	0.9998/1.3645
rs104893836 (T_C) GNRHR	Hypogonadotropic hypogonadism 7 without anosmia. (PMIM:146110). Single gene listed in OMIM.	Pathogenic; rare disorder	AR	0/1/290	0.0017/0.0012/0.0055	1.4167/0.3069
rs2476601 (A_G) PTPN22	Susceptibility to T1DM (PMIM:222100). Also associated with susceptibility to Systemic lupus erythematosus (PMIM:152700). OMIM lists multiple genes.	Risk Factor; common disorder	AR	0/5/280	0.0292/ 0.0274/0.0131	1.00/0.98
rs17158558 (C_T) RET	Susceptibility to hirschsprung disease 1 (PMIM 142623). Single gene listed in OMIM.	Risk Factor; common disorder	AD	0/15/273	0.02577/0.0219649/0.045867	1.1732/0.5618
rs121918219 (G_A) VANGL1	Susceptibility to neural tube defects. (PMIM: 182940); OMIM lists 5 genes.	Risk Factor; common disorder; >100	AD	0/1/289	0.001718/./0.001007	./1.7060
**D**. **10 Instances wherein different mutations from the same gene are seen in arab studies (generally seen in omim but not in our exomes)**.						
**7 Rare & deleterious variants**						
rs121908736 (G_A) *ADA*	Partial adenosine deaminase deficiency (PMIM:102700). **Arab studies:** CAGS reports another mutation from the same gene p.Arg282 > Gln^126^.	Pathogenic; rare disorder	AR, SM. Compound heterozygosity	0/2/285	0.0035/0.0018/0.0005	1.9444/6.9444
rs61757582 (G_A) *DHCR7*	Smith-Lemli-Opitz syndrome (PMIM:270400). **Arab studies:** N287K (861 C > A); R352Q (1055 G > A) (rs121909768; seen in OMIM); and R352L (1055 G > T)^127^.	Pathogenic; rare disorder	AR	0/1/284	0.0018/0.0002/.	9/.
rs61753185^$^ (G_A) *TYR*	Tyrosinase-negative oculocutaneous albinism, Type IA (PMIM:203100). **Arab studies:** c.817 G > C/p.W272C (rs62645902; not seen in OMIM)^108,128^.	Pathogenic; rare disorder	AR	0/1/290	0.0017/0.0004/0.0005	4.25/3.3730
rs116100695 (G_A) PKLR	Pyruvate kinase deficiency of red cells (PMIM:266200). **Arab studies:** 1058 C > T; Thr353Met (rs74315362 seen in OMIM)^129^.	Pathogenic; rare disorder	AR	0/2/289	0.0034/0.0016/0.0096	2.125/0.3547
rs41295338 (G_T) *TGM1*	AR congenital ichthyosis 1 (PMIM:242300). **Arab studies:** Compound heterozygosity for missense mutations (R141H- rs121918718, R142H-rs121918719), (p.Tyr136Ter-rs1057517836), (p.Ser326Cysfs*8), and Leu362Arg^130,131^	Pathogenic; rare disorder	AR	0/2/288	0.0034/0.0022/0.0060	1.5454/0.5627
rs121434513 (G_C) *PNKD alias MR1*	Paroxysmal Nonkinesigenic Dyskinesia 1 (PMIM: 118800). **Arab studies:** c.20 C > T; A7V rs121434512 – seen in OMIM^132^.	Pathogenic; rare disorder	AD	0/1/289	0.0017/0.0002/0.0005	8.5/3.3530
rs34424986 (G_A) *PARK2*	Parkinson disease, juvenile, type 2. (PMIM:600116). Arab studies: exon 4 deletion and a 2-base AG deletion in exon 2 (101–102) from the same gene associated with the disorder^133^. Not seen in OMIM. This table reports another variant rs137853054 seen in Arab study and in Kuwaiti exomes^133–135^.	Pathogenic; rare disorder 11–50	AR	0/2/289	0.0034/0.0004/.	8.5/.
**3 Cags arab disorders**						
rs12021720 (T_C) *DBT*	Maple syrup urine disease, intermediate, type II (PMIM:248600). **Arab studies:** c.1281 + 1 G > T in one patient from UAE. Not seen in OMIM^136^.	Pathogenic; rare disorder	AR	5/67/218	0.1347/0.1082/0.12 The variant is in compound heterozygosity with [rs121965001]^46^. Hence high MAF is acceptable.	0.97/0.98
rs1800858 (G_A) *RET*	Susceptibility to Hirschsprung disease 1; HSCR1 (PMIM: 142623). **Arab studies:** c.1852T > C Cys618Arg mutation rs76262710. Seen in OMIM (for neoplasia)^137,138^.	Risk Factor; common disorder; 11–50	AD	25/128/138	0.3058/0.2464/0.2593	1.24/1.1793
rs121918530 (A_G) *MEF2A*	Coronary artery disease/myocardial infarction (PMIM:600660). **Arab studies:** (rs1059759 G > C) in Saudi Arabian patients^139^.	Risk factor by inference for complex disorder.	AD	0/6/284	0.01031/0.000399361/0.002014	25.8162/5.119
**E**. **7 instances wherein the Arab studies report different mutation from different gene (but associated with the disorder in OMIM)**. **These disorders involve multiple genes**. **In general**, **the different gene and the different mutations are seen in OMIM associated with the disorder**. **The Arab mutations are not seen in Kuwaiti exomes**.						
rs11909217 (C_T) *LIPI* **RARE & DELETERIOUS**	Susceptibility to Hypertriglyceridemia, Familial (PMIM: 145750). OMIM lists LIPI and APOA5 for the disorder. **Arab study:** 3′ UTR C > G variant from APOC3 (not seen in OMIM or our exomes)^95^. This is an inherited common disorder.	Risk factor by inference; common disorder.	AD	0/10/280	0.0172/0.006/0.01309	2.8667/1.3138
rs3135506 (G_C) *APOA5* **CAGS VARIANT**		Risk factor; Common disorder	AD	1/22/267	0.04124/0.0557109/0.070565	0.7402/0.5844
**rs28932472 (G_C)** ***POMC*** **RARE & DELETERIOUS**	Obesity, early-onset, susceptibility to (PMIM: 601665). OMIM lists several genes. **Arab study:** rs1042713: Arginine 16 Glycine (Arg16Gly) polymorphism in ADRB2 gene; which is associated with susceptibility to nocturnal asthma in OMIM and our study. rs1042713 A_G: (AA:55, AG:126, GG:82) & (MAF_KWT, 1KGP, GME: 0.5513/0.5244/0.5645)^102^.	Risk Factor; common disorder	AR,AD,MF	0/1/287	0.0017/0.0024/0.0005	0.7083/3.3730
rs34911341 (C_T) *GHRL* **RARE & DELETERIOUS**		Risk Factor; common disorder	AR,AD,MF	0/1/290	0.0017/0.0026/0.0035	0.6538/0.4823
rs2282440 (G_A) *SDC3* **CAGS variant**		Association variant; common disorder; >100	AD	0/11/280	0.0189/0.0189/0.0282	0.9999/0.6703
rs11558538 (C_T) *HNMT* **SAFD variant**	Susceptibility to Asthma (PMIM: 600807). OMIM lists several genes. Arab studies: 786 T > C [rs2070744] from the 5’ flanking region of NOS3 (OMIM lists this variant but the variant is not seen in Kuwaiti exomes)^140^.	Risk Factor for common disorder	AD	7/55/212	0.1259/0.0595/0.0968	2.1159/1.3009
rs1801483 (G_A) *GCGR* **SAFD Variant**	T2DM (PMIM: 125853). **Arab studies:** Ala222Val (rs1801133 C > T AR/AD) mutation from MTHFR gene in Lebanese patients with T2DM and nephropathy^123,124^. The rs1801133 is seen in Kuwaiti exomes (MAF:KWT/1KGP/GME = 0.1810/0.2454/0.2557; AA/AG/GG: 12/81/197). Another *MTHFR* variant Glu429Ala (rs1801131 T_G AR) in homozygous form has protective effect in Israeli Jews^123^ and South Indians^125^. The rs1801131 variant is seen in Kuwaiti exomes (MAF: KWT/1KGP/GME = 0.3265/0.2494/0.3353; GG/GT/TT: 31/128/132).	Risk factor by way of inference	AD	0/22/269	0.0378/0.0042/0.0311	9/1.2148

### Identified variants and the associated complex disorders

Examining our in-house genome-wide association study (GWAS) data (on 1351 native Kuwaiti Arab individuals genotyped on Illumina HumanOmniExpress BeadChip and 1900 native Kuwaiti Arab individuals genotyped on Illumina HumanCardio-Metabo BeadChip) for the presence of the variants identified through exome analysis revealed that 27 of the identified OMIM-listed causal variants present in Kuwaiti exomes were also seen in the GWAS data. Allele frequencies and carrier distributions as seen in exomes data set and GWAS data set are presented (Table 9). 13 of these 27 variants were associated with disorders observed in Arab studies. The 27 variants were pharmacogenomic (11), SAFD (9) and CAGS (5 + 2) variants for complex disorders. The allele frequencies among the data sets of Kuwait exome, Kuwait GWAS and GME were comparable with each other; and the carrier distributions were similar between the Kuwaiti exomes and GWAS data sets.

**Table 9:** Comparison of allele frequencies & carrier distribution of the reported variants from Kuwaiti exome data set with a larger data set of our in-house genome-wide genotype data.

dbSNP ID; Ref_risk; gene	Type of variant & whether the variant has been observed in Arab studies	Associated disorder	Risk allele frequency in Kuwaiti exomes/GME & Carrier distribution in Kuwaiti exomes	Risk allele frequency in Kuwaiti GWAS data set & Carrier distribution in GWAS data
rs699 (A_G)	Pharmacogenomic variant for hypertension;	PharmGKB listed this variant as drug response for Hypertension). (OMIM lists this variant as susceptibility to Essential hypertension (PMIM:145500). **Arab study** implicates this in Reduced life-span through genetic susceptibilities to both Essential Hypertension and Myocardial Infarction in UAE^101^.	0.583/0.5534	0.5658
*AGT*	Arab mutation		91/127/47	439/653/260
rs1805087 (A_G)	Pharmacogenomic variant for Rheumatoid arthritis.	Arab study implicates this in Autism – North Iran^97^.	0.2603/0.2021	0.2262
*MTR*	Arab variant.		23/105/162	77/456/819
rs1367117 (G_A)	Pharmacogenomic variant for hypertension	Hypertension	0.1448/0.1727	0.1647
*APOB*			5/74/211	33/379/939
rs2960306 (G_T)	Pharmacogenomic variant for hypertension.	Hypertension	0.371/0.362	0.3607
*GRK4*			42/129/166	182/612/559
rs1024323 (C_T)	Pharmacogenomic variant for hypertension	Hypertension	0.3718/0.3887	0.3544
*GRK4*			33/137/103	172/608/562
rs1801058 (T_C)	Pharmacogenomic variant for hypertension	Arab study implicates this as risk factor for myocardial infarction among hypertensive subjects in Jordan^100^. PharmGKB listed this variant as drug response for Hypertension). (OMIM does not list this variant).	0.229/0.292	0.2627 91/529/733
*GRK4*	Arab mutation		16/101/173	.
rs1042713 (A_G)	Pharmacogenomics variant for asthma.	Susceptibility to early onset obesity, insulin resistance *etc*.) in Saudi Arabia^102^.	0.5513/0.5645	0.5814
*ADRB2*	Arab mutation	OMIM listed this variant for susceptibility to asthma, nocturnal PMIM:600807).	82/126/55	376/533/196
rs1800888 (C_T)	Pharmacogenomics variant	Drug response for asthma and heart failure	0.01211/0.0156	0.014
*ADRB2*			0/7/282	2/33/1316
rs10509681 (T_C)	Pharmacogenomics variant for diabetes and cancer.	Drug response for Rosiglitazone and Repaglinide	0.1197/1047	0.1223
*CYP2C8*			3/63/222	32/266/1053
rs1048943 (T_C)	Pharmacogenomics variant.	Warfarin sensitivity	0.065/0.0619	0.042
*CYP1A1*			3/32/256	4/101/1216
rs1042522 (G_C)	Pharmacogenomics variant.	Drug response for stomach neoplasm.	0.443/.	0.4039
*TP53*	Arab variant	Susceptibility to Breast Cancer in Tunisia^98^	57/140/90	236/620/496
rs861539 (G_A)	SAFD variant	Cutaneous malignant melanoma 6, susceptibility to	0.3864/0.3836	0.3843
*XRCC3*	AD	PMIM: 613972	35/151/100	166/517/422
rs35264875 (A_T)	SAFD variant	Skin/hair/eye pigmentation, variation in, SHEP10	0.1832/0.1677	0.1906
*TPCN2*	Association by GWAS	PMIM: 612267;	12/72/178	59/397/895
rs1260326 (C_T)	SAFD variant	FPG quantitative trait locus 5	0.3945/0.5388	0.469
*GCKR*		PMIM: 613463	47/134/108	312/645/396
rs7076156 (G_A)	SAFD variant	Uric acid nephrolithiasis, susceptibility to;	0.3351/0.28	0.3045
*ZNF365*		PMIM: 605990	33/129/129	130/562/659
rs5030737 (G_A)	SAFD variant	Mannose-binding protein deficiency. Complex trait	0.0790/0.0535	0.0673
*MBL2*	AD	PMIM: 614372	2/39/231	6/167/1177
rs6232 (T_C)	SAFD variant.	Obesity, susceptibility to, Body mass index quantitative trait locus 12	0.0594/0.0524	0.046
*PCSK1*		PMIM: 612362	1/32/253	2/119/1227
rs1801280 (T_C)	SAFD variant	Slow acetylator due to N-acetyltransferase enzyme variant. Toxicity to the drugs of cisplatin or cyclophosphamide.	0.3927/0.434	0.433
*NAT2*		PMIM: 243400	41/134/100	182/483/313
	AR	Arab mutation.		
rs4792311 (G_A)	SAFD variant	Prostate cancer, hereditary, 2′; Complex trait	0.3552/0.3092	0.3396
*ELAC2*		PMIM: 614731	30/146/114	167/583/602
	AR or AD?			
	Has to be in compound heterogeneity with rs5030739			
rs2108622 (C_T)	SAFD variant – drug response.	Acenocoumarin response – Dosage. Warfarin sensitivity PMIM: 122700	0.4102/0.3983	0.4146
*CYP4F2*			53/127/104	239/643/469
rs1801131 (T_G)	CAGS; Arab mutation	Schizophrenia; sczd. (PMIM:181500). OMIM lists several genes.	0.3265/0.3353	0.3527
*MTHFR*			31/128/132	146/659/544
rs4149584 (C_T)	CAGS variant	Periodic fever, familial, autosomal dominant. (PMIM:142680).	0.0069/0.0121	0.0113
*TNFRSF1A*	Genetics basis for the disorder has not been reported in Arab studies. AR		0/4/287	0/13/606
rs2282440 (G_A) *SDC3*	CAGS variant AD	Obesity, early-onset, susceptibility to (PMIM: 601665).	0.0189/0.0282 0/11/280	0.0147 0/11/361
rs17158558 (C_T)	CAGS variant	Hirschsprung disease, susceptibility to, 1 (PMIM 142623). Complex disorder. Genetics basis is not reported in Arab studies.	0.0260/0.0459	0.0325
*RET*	AD		0/15/273	2/84/1266
rs1799945 (C_G)	CAGS variant	Hemochromatosis, type 1 -microvascular complications of diabetes, susceptibility to, 7, included. (PMIM:235200). It is a common disorder.	0.1219/0.1198	0.1156
*HFE*	AR		4/63/224	23/264/1061
rs1801133 (G_A) *MTHFR*	AR Arab mutation	Susceptibility to T2DM in Lebanese^123,124^	0.1810/0.2557 12/81/197	0.255 511/89/751
rs1801131 (T_G) *MTHFR*	AD Arab mutation	Protective effect T2DM in Israel Jews^123^ and in South Indians.	0.3265/0.3353 31/128/132	0.3527 147/660/546

## Discussion

In this study, exomes from 291 healthy, unrelated native Kuwaiti Arabs were analysed to identify 170,508 SNVs and 3,341 indels. 12% of SNVs and 28% of indels were novel. One-third of the identified SNVs were population-specific, and 21.7% were ‘personal’ (observed in only one Kuwaiti exome and not seen in GME or 1KGP), consistent with the results of other studies on ethnic populations, including those from Qatar^44^, Spain^17^ and Denmark^12^. 53% of the identified SNVs were missense, and an average of 1.3% of the 14,557 SNVs that each person carried were predicted to affect protein function. Allele frequencies in 6,186 SAFD variants were significantly different from those observed in 1KGP populations.

Recent population genetic analyses have demonstrated that humans harbour an abundance of rare & deleterious variations, with >80% of all coding variants having a frequency of ≤1%^10,14,52^. In this study, a majority (51%) of the identified SNVs in Kuwaiti exomes were rare. Of the identified 55,644 population-specific SNVs, only 138 were ‘common’, and the rest were ‘rare’ or ‘low-frequency’. Up to 60% of the population-specific variants were missense changes, and 51% of LoF variants were population-specific (some of which were polymorphic). These observations support the notion that coding variants with allele frequency of <1% show increased population-specificity and are enriched for functional variants^13^. Human populations have experienced recent explosive growth, expanding by at least three orders of magnitude over the past 400 generations; such a rapid recent growth along with weak purifying selection has increased the load of rare variants, many of which are deleterious and relevant for understanding disease risks^14,16^.

On average, nearly 10.4% of Kuwaiti population-specific variants found in every Kuwaiti individual were homozygous; this extent of homozygosity, which is higher than that observed in other ethnic populations (such as the value of 7.05% in Spanish^17^), reflects the higher rate of consanguinity practised among the Kuwaiti Arab population. The GME study^26^ demonstrated an increased burden of runs of homozygosity in Greater Middle East populations; our previous works had shown that Kuwaiti population is heterogeneous (placed between populations that have large amount of ROH and the ones with low ROH) with the KWS subgroup as highly endogamous^24^. An average of 73 LoF variants (of which 4.5 were Kuwaiti-specific) were seen per individual. Observed disease-causing mutations failing to cause disease in at least a proportion of the individuals who carry them has been extensively discussed^53^. On an average, only 4.67% of Kuwaiti-specific LoF variants per individual were seen homozygous (as opposed to the expected 10.4%) and such a reduced homozygosity among LoF variants may explain the reduced penetrance.

Rare homozygous loss of function variants are supposed to exhibit strong signs of selective pressure. Of the genes harboring the identified 36 rare (MAF <2.0%) homozygous putative LoF variants observed in Kuwaiti exomes, only 8 were seen common with published list of inactivated genes from Icelanders^42^ and only 1 was common with the list from ExAC^8^. These findings suggest that the set of non-clinically relevant loss-of-function variants is far from being complete^26^ and consideration of ethnic populations with consanguinity as in the GME study and our study can augment the list of human knock-out events.

We previously catalogued^36^ exome variants from 15 native Kuwaiti individuals of KWS subgroup (city-dwelling Saudi Arabian tribe ancestry^24^) and postulated that further samples were needed to capture the full spectrum of exome variability. The present study indicated that our previous work captured only a portion (22%) of variability. The repertoire of ‘all’ SNVs and ‘population-specific’ variants increased with the number of samples sequenced and did not reach a plateau (Fig. 2). However, once population-specific variants were divided into personal and genuine polymorphic variants, the later reached a plateau. These data suggested that most of the Kuwaiti-specific polymorphisms within coding regions were restricted to approximately 10,000 positions (Fig. 2, dashed blue line).

Utility of whole-exome data in population structure analysis produces results congruent to those obtained using genome-wide genotype data^54^. In this study, principal component analysis of the merged data set of exome variants from Kuwait, 1KGP global populations, and Qatar confirmed the existence of three subgroups (Fig. 1) previously derived from genome-wide genotype data^24^ in Kuwaiti population. The KWB subgroup showed greater genetic affinity towards African populations, and the other two clearly demarcated subgroups (namely KWP and KWS) were between South Asian and European populations. Furthermore, the three substructures of the Qatari population^25^ lied akin to Kuwaitis. These results were supported by evidence from *pF*_*ST*_ likelihood ratio tests, which identified variants that differentiated the subgroups. The population-wide occurrence of Kuwaiti SAFD variants in the context of maximum allele frequency populations indicated pairing of Kuwaiti individuals mostly with Europeans and Ashkenazi Jewish populations from 1KGP phase 3 and gnomAD data sets. Such genetic relatedness among Middle Eastern, European and Ashkenazi Jewish populations was further confirmed through performing population genetic analyses (*F*_*ST*_ and PCA) by way of including genotype data from Ashkenazi Jews as well (Figs 4 and 5). Notably, in line with previous studies^43^, population genetic analysis presented in this study demonstrated the genetic relatedness among Middle Eastern, European and Ashkenazi Jewish populations.

The Kuwaiti exomes, presented in this study, included 46 clinically significant deleterious variants that are rare in global populations and in Kuwaiti exomes (except for three) (pathogenic: 35; drug response:1; risk factor: 10). 28 of the 36 pathogenic variants followed AR mode of inheritance and the 7 of the 10 risk factor variants followed AD mode of inheritance. Disorders associated with 20 of the 46 variants were seen in Arab populations. Three of the 46 variants reached an MAF value characterizing low-frequency variants in Kuwaiti exomes; two of these three were risk factor and one was drug response variant; and the allele frequencies were comparable with GME data set (see Table 3)-(rs61742245-*VKORC1*:1.04%,1.37%; rs1800553-*ABCA4*:2.41%,2.1%; rs11909217-*LIPI*:1.72%,1.31%). The three variants indicating high risk ratios in Kuwaiti exomes for disease pathogenesis and response to medication were: the *VKORC1* variant was associated with warfarin resistance (AD) (heterozygous in four individuals and homozygous recessive in one individual), the *ABCA4* variant was associated with susceptibility to age-related macular degeneration (AD) (heterozygous in 12 individuals and homozygous recessive in one individual), and the *LIPI* variant was associated with susceptibility to hypertriglyceridemia (AD) (heterozygous in 10 individuals). Mendelian and rare genetic disorders as well as monogenic forms of common complex diseases are often associated with rare coding variants. The rare coding variants can have remarkably different allelic frequencies in different ethnic populations compared with the 1KGP populations^10,55^. The data presented above reported rare variants associated with not only rare Mendelian disorders but also with complex disorders. This observation is in agreement with literature reports on many examples of rare and low-frequency variants associated with complex phenotype traits and common disorders (a review of some of the relevant studies are as listed in Table 1 in Schork *et al*.^56^). An interesting example of rare & deleterious “risk factor” variants associated with increasing risk for complex disorders was the *GHRL* variant (rs34911341-C/T; Arg51gln) which OMIM associated with susceptibility to the complex disorder of obesity (along with genes such as *POMC*, *SDC3* and *ADRB2*); the variant was originally seen in 6.13% of 96 unrelated Swedish female subjects of morbid obesity (BMI 42.3 ± 3.4 Kg/m^2^)^57^. The variant had been seen in GME data set and in one individual from our study cohort; incidentally, the individual was morbidly obese female with a BMI of 44.3 kg/m^2^; Though our study cohort consisted 48 morbidly obese female individuals, only one of them carried this GHRL allele.

The study identified in Kuwaiti exomes a set of 21 missense SNVs (that were predominantly ‘common’ in both Kuwaiti exomes and 1KGP populations as well as in GME) mapping to drug-binding domains and were of pharmacogenomic relevance (relating to complex disorders, such as sickle-cell anaemia, hypertension, diabetes, asthma, cancer and chemical dependence). 7 of these 21 variants were also observed as Arab mutations associated with complex disorders in Arab populations (Table 5). Of the 21 pharmacogenomic SNVs, the CYP2C8*3 variants encoding two linked amino acid substitutions^46^ were particularly evident (Table 4) in Kuwaiti exomes; risk allele frequencies at these two variants were 12% in Kuwaiti exomes, 10.4% in GME and 4.6% in 1KGP; the risk alleles co-segregated in 33 individuals in our study cohort. *CYP2C8* has emerged as a significant pharmacogene^58,59^ and is responsible for biotransformation of 5% of currently used drugs that undergo phase 1 hepatic metabolism^60^. The CYP2C8*3 variants regulate the dosage of the diabetes drugs rosiglitazone and repaglinide^61,62^. The minor alleles of the CYP2C8*3 variants were also associated with decreased metabolism of paclitaxel^59^. The *ADRB2* variant (occurring with an MAF of 1.2% in Kuwaiti exomes, 1.6% in GME and 0.4% in 1KGP) regulates the efficacy of the asthma drug terbutaline and beta-blocking agents used to treat heart failure^63^. *ADRB2* variant had also been correlated with the risk of type 2 diabetes, obesity and hypertension^63^; six individuals from our study cohort carried risk allele at this variant.

The identified 24 SAFD variants (all of which were missense variants – see Table 6) with clinical significance included (a) two pathogenic variants (with AR mode of inheritance) associated with the rare disorders of Corticosterone methyloxidase type 2 deficiency and Carboxypeptidase N deficiency); (b) four drug response’ variants associated with toxicity to the drugs of cisplatin or cyclophosphamide and with response to anti-coagulation drugs; (c) sixteen risk/protective’ variants associated with complex traits (*ex*. asthma, Parkinson’s disease, obesity, nephrolithiasis, melanoma 6, and alcohol dependency); and (d) two ‘Associated’ variants relating to traits of FPG levels and skin/hair/eye pigmentation. The gnomAD populations that showed highest MAFs at these 24 variants were Ashkenazi Jews (15 instances) and Europeans including Finnish (6 instances). The 1KGP populations that showed the highest MAFs were Europeans (14) and South Asians (7). As expected, a major number of these variants were ‘common’ (20 in Kuwaiti exomes and 14 in 1KGP data). The associated disorders are common in the region - Cholestasis of pregnancy (associated with the *ATP8B1* variant), the most common AD disorder in pregnant women, has an incidence rate of 0.8%–1.46% in South Asian populations^64^; Hereditary prostate cancer (associating with the two *ELAC2* variants) is one of the most prevalent cancers in Kuwait^65^; The corticosterone methyloxidase type 2 deficiency (associating with the *CYP11B2* variant) is more common in people of Iranian Jewish ancestry^66^; and Coronary artery disease (associating with the *MEF2A* variant) has an incidence rate of approximately 6% in the Saudi Arabian population^67^.

In addition, two other SNVs from our analysis for functional variants were seen associated with quantitative traits in GWA studies – an SAFD LoF variant rs2228015/*CCR7* associated with the complex hematological trait of lymphocyte count in European-ancestry people^68^ and a missense variant (rare in 1KGP but common in Kuwaiti exomes) rs117135869/*TTC38* associated with a novel complex metabolic quantitative trait loci (mQTLs) in a cohort from Middle Eastern population^48^. Further search for presence in Kuwaiti exomes of OMIM-listed causal variants relating to CAGS disorders led to a list of additional 17 variants (see Table 7); 7 of these variants were “pathogenic” and the remaining 10 were “risk factor” variants. The analysis identified a total of 25 CAGS disorders for which the OMIM-listed causal variants were seen in Kuwaiti exomes; such a poor turnout of only 25 was probably due to the small size of the cohort.

Of the 112 variants of clinical significance discussed so far, as many as 44 were ‘common’ variants in Kuwaiti exomes. Very often these common variants were relating to complex disorders. In this study, we did not ourselves delineate the variants associated with complex disorders; we rather just examined whether and which of the functional variants identified in our study were annotated in OMIM, ClinVar, PharmGKB, and literature as associated with complex (or rare) disorders. A question arose as to whether the study cohort of 291 exomes had enough power. Of the 112 variants, 27 variants (comprising 1 rare, 3 low-frequency and 23 common variants) were also seen in our in-house GWAS data set of larger sample size; the set of 27 variants comprised 11 pharmacogenomic, 9 SAFD, 5 CAGS and the two MTHFR variants associated with susceptibility to T2DM (Table 9). The MAF at these variants were comparable among the Kuwaiti exomes, GWAS data, and the GME data set; the carrier distributions were also comparable with one another among the Kuwaiti exomes and GWAS data set.

Finally, disorders relating to 52 of the identified variants were observed in Arab population. Inheritance modes associated with these 52 variants were: 28 autosomal recessive, 15 autosomal dominant, and 9 ambiguous. 25 of these variants were relating to ‘rare’ and 27 were relating to complex disorders. This study (based on 291 exomes) provided data on 23 known Arab mutations for 23 disorders seen in Arab populations, data on 12 putative mutations for 12 disorders observed but not yet characterized for genetic basis in Arab population, and data on 17 additional putative mutations for disorders characterized for genetic basis in Arab populations. This data is useful for testing in future case-control studies.

Capturing the extent of genetic variation in Middle East region is poorly represented in global studies. However, the Greater Middle Eastern (GME) Variome Consortium^26^ has recently made a notable effort to address this concern by way of capturing genetic variations from exomes of 1,111 unrelated and supposedly healthy individuals from Northwest and Northeast Africa, Turkish peninsula, Syrian desert, Arabian Peninsula and Persia & Pakistan. The GME data set included 214 exomes from Arabian Peninsula (AP), of which 45 are from Kuwaiti population. Our study consisting of 291 Kuwaiti samples, sourced from the 3 Kuwaiti population subgroups, complements and augments the GME genetic variation data by way of presenting a higher number of exomes representing a single state of AP namely Kuwait. It is further the case that the GME study discovered and presented the variegated genetic architecture in GME populations; this is complemented by the population genetics results from our study from a relatively larger sample set of native Arabs living in a single state from the Peninsula. The GME study demonstrated the utility of the GME exome data set in discovering the genetic basis of Mendelian disorders in Greater Middle Eastern populations; our study provides data on Arab mutations for 23 disorders and points to 31 OMIM-listed variants relating to disorders seen in Arab populations for testing in future case-control studies.

A potential limitation of this study arises from the number of exomes sequenced. Though the number of population-specific variants seemed to saturate with 291 exomes, the total number of “all” identified variants did not saturate (Fig. 2); this indicates that we need to sequence furthermore samples to sufficiently represent the Arab population from Kuwait. It is further the case that variants associated with only a small set of disorders observed in the region were seen in the reported Kuwaiti exome data.

In conclusion, the presented assessment of 291 exomes of unrelated healthy individuals unveiled the prevalence of rare as well as common variants related to various Mendelian disorders and common complex diseases that are predominantly inherited as recessive. The inclusion of different genome data sets in our analyses highlighted similarities in allele frequencies among Arabs and Jews, and among nomadic Bedouins and Africans. Furthermore, our data corroborates the Kuwaiti population substructures previously determined by genome-wide genotype data; the results on population structures from Kuwait is generally in agreement with the variegated genetic architecture seen in Greater Middle Eastern populations^26^. The striking occurrence of pharmacogenomic variants relating to common complex disorders, underlines the importance and need for cataloguing genetic variants in similar Arab populations of the Middle East region. This study is a significant addition to regional data resources (such as GME^26^) and global resources (such as 1kGP^3,4^) on human exome variability; however, a wide range of similar studies in the region are warranted to support genomic discoveries in medical and population genetics at the regional and global levels^26^.

## Methods

### Ethics Statement

The protocols used in the study were approved by the International Scientific Advisory Board and the Ethical Review Committee at Dasman Diabetes Institute, Kuwait. Written informed consent was obtained from participants before collecting blood samples. Identities of the participants were protected from public exposure, and samples/data were processed anonymously. All methods were performed in accordance with the relevant guidelines and regulations.

### Selection of subjects for whole-exome sequencing

To capture the extent of exome variation in the entire Kuwaiti population, 291 healthy, unrelated native Kuwaiti individuals from the study cohorts used in our earlier studies were selected^24,36,69,70^. At the time of recruitment, all participants in this study were healthy and deemed free of Mendelian or rare genetic disorders, cognition or physical disability, mental retardation or chronic disorders, such as cancer. Distribution of the selected participants in three subgroups of Kuwaiti population^24^ was as follows: 109 in KWS (Saudi Arabian tribe ancestry), 126 in KWP (Persian ancestry) and 34 in KWB (nomadic Bedouin ancestry).

### Whole-exome sequencing

High-quality DNA samples were enriched for exomes using TruSeq Exome Enrichment kit and the Nextera Rapid Capture Exome kit (Illumina Inc. USA). The captured libraries were then clustered using TruSeq Paired Cluster Kit V3 (Illumina Inc. USA) and sequenced in HiSeq 2000 using Illumina’s Sequence by Synthesis technology as 100 paired-end reads.

### Exome data analysis

The HugeSeq^71^ computational pipeline was used to automate the variant discovery process. Sequence reads were aligned to the reference human genome build hg19 using BWA^72^. Prior to variant calling, alignment files were processed using the Genome Analysis Toolkit (GATK)^73^. Post-alignment procedures included PCR duplicate removal, local realignment around known indels and base quality recalibration. Best practices for the GATK workflow were followed, and standard hard filtering parameters^74,75^ were used for variant discovery from the processed alignment files. Variant calling on each sample’s BAM file was performed using HaplotypeCaller followed by joint genotyping analysis of the resultant gVCFs to create raw SNV and indel VCFs. Variants called in the sequenced exomes were restricted to intervals covered by both TruSeq (163 samples) and Nextera (128 samples) Exome Enrichment kits. To improve the quality of the data set, the resulting variant call sets were filtered by setting sample variant thresholds at ≥10X depth, <180X depth and genotype quality of >20. Variants with allele balance of <30% were removed to filter out sites where the fraction of non-reference reads was too low. Hardy–Weinberg Equilibrium was assessed using an exact test, as defined by Wigginton *et al*.^76^, and excluded sites with p-values of <10^−5^. Lastly, all variants with a call rate of <90% were excluded. Thus, after the variant quality filtering steps, only the consensus of variants determined using both kits appeared in the final VCFs.

### Classifying the variants

The Ensembl genome database build 75 was used as reference for gene annotation. SNP Variation Suite (SVS) v8.7.1 from Golden Helix Inc^77^ was used to derive functional classifications of the identified variants. The identified SNVs and indels were categorised as ‘known’ and ‘novel’ based on the content of the single-nucleotide polymorphism database of dbSNP146^78^. Variants already reported in dbSNP146 were annotated as ‘known’, and the others were annotated as ‘novel’. Variants observed in only a single exome from the study cohort and not seen in 1KGP or GME data sets were annotated as ‘personal’. Variants (excluding the ‘personal’) that were not observed in 1KGP phase 3 data were annotated as ‘population-specific’, and population-specific variants observed in more than one exome from the study cohort were annotated as ‘population-specific polymorphic’ variants. Variants leading to stop gain, stop loss, frameshift and damage in splice sites were annotated to cause LoF (loss of function). Variants were classified as ‘rare’ if MAF was <1% (personal variants were not considered as rare), as ‘low-frequency’ if MAF was 1–5% and as ‘common’ if MAF was ≥5%.

### Principal component analysis of the merged set of exome variants from Kuwaiti and global populations

The 1KGP phase 3 exomes of 2,504 individuals from 26 populations, covering the four continents of Asia, Africa, America and Europe and 100 exomes of Qatari individuals^44^ were considered along with Kuwaiti exomes. An LD-pruned (LD threshold of 0.5) data set of 20,215 variants (having MAF of ≥5%) observed in all three Kuwaiti subgroups and the regional and global populations was created. Golden Helix SVS software v8.7.1 was used to perform principal component analysis with the merged data set.

### *pF*_*ST*_ likelihood ratio tests: Comparison of allele frequency distribution among the Kuwaiti population subgroups

Reference alleles and alternate alleles were binned to set the standard for ‘Kuwaiti exome’. In order to detect alleles driving differentiation among the three Kuwaiti subpopulation groups of KWP, KWS and KWB, *pF*_*ST*_ likelihood ratio tests^79^ for allele frequency differences in autosomal variants (filtered for missingness rate and deviation from Hardy–Weinberg equilibrium) were performed.

### Identification of SNVs with significant differences (SAFD variants) in allele frequencies between Kuwaiti and global populations

Autosomal SNVs observed in both Kuwaiti exomes and 1KGP phase 3 exomes^4^ were identified, and SNVs for which minor alleles were not observed in Kuwaiti exomes were excluded. SAFD variants that exhibited significant allele frequency differences were identified by performing one-sided binomial exact tests (allele frequencies in 1KGP global populations were considered as ‘expected’), followed by Bonferroni correction. A p-value threshold of 0.05 was used to assess the significance of allele frequency differences. ClinVar^80^ data resource was used to assess the clinical significance of the identified SAFD variants. In the context of population structure analyses, populations from gnomAD data set^8^ were also used to compare allele frequency distributions. The comprehensive scrutinization of population-wide occurrence was performed by considering the paired incidence of populations with maximum allele frequency.

### Principal Component Analysis of the merged set of Kuwaiti exomes, Ashkenazi Jews, 1KGP phase 3 and Qatar and *F*_*ST*_ analysis

We combined Kuwaiti exomes with the data sets from Ashkenazi Jews^43^, 1KGP phase 3^4^ and Qatar^44^. The combined data set of coding-region variants was cleaned and LD-pruned to obtain a total of about 896 variants and 3,336 individuals representing world populations. Principal component analysis (PCA) was performed using smartpca in the EIGENSOFT software package (v 6.1.4)^81,82^. Two-dimensional and three-dimensional scattered PCA plots were created using RStudio^83^ (v 1.1.423). Mean pairwise *F*_*ST*_ values and the matrix between populations were generated using PLINK^84^ (v 1.9). The *F*_*ST*_ heatmap was created using RStudio (v 1.1.423).

### Examining OMIM and ClinVar annotations for inferring clinical significance of SNVs

OMIM and ClinVar should mention the Kuwaiti exome SNV, with literature evidence and citation reference, as an associated variant for a disorder; the dbSNP identifier of the SNV and the observed risk allele should be mentioned as such in the OMIM and ClinVar annotation^80,85^. The clinical significance for the variant should be mentioned consistently with the same term (such as ‘pathogenic’ or ‘risk factor’ or ‘protective’ or ‘drug response’) in all the records for the disorder; it should not be the case that few records list the significance as ‘pathogenic’ and few other records list as ‘benign’ or ‘conflicting interpretation’ for the disorder; ClinVar records listing “not specified” for the data item of ‘conditions’ were not considered. As is the practice^86^, in cases of ClinVar variants with conflicting annotation for clinical significance, evidence from a peer-reviewed publication and manually curation (OMIM) takes precedence over evidence from clinical testing submissions. ClinVar defines “Pathogenic” variants as those that are interpreted for Mendelian disorders; or as those that have low penetrance; “Drug response” variants as those that affect drug response, and not a disease; “Risk factor” variants as those that are interpreted not to cause a disorder but to increase the risk; “Association” variants as those that were identified in a GWAS study and further interpreted for their clinical significance; “Protective” variants that decrease the risk of a disorder, including infections; and “Susceptibility to” variants that increase the risk of a disorder. In those instances, wherein ClinVar annotated a variant as “pathogenic” but the associated disorder was “complex or common or more prevalent in the study population” or the patient carrying the variant was annotated in OMIM as susceptible to the disorder (which is often a common disorder), we reannotated the variant as “Risk factor by inference”.

### Classifying disorders as ‘rare’ or ‘complex’

Various resources that were examined to ascertain whether the disorder is rare or common: Catalogue of Transmission Genetics in Arabs (available at http://cags.org.ae/ctga/), Genetic and Rare Disease (GARD) Information Centre (available at https://rarediseases.info.nih.gov/diseases/), Genetics Home Reference (available at https://ghr.nlm.nih.gov/), Medscape (available at https://geneaware.clinical.bcm.edu/GeneAware/AboutGeneAware/DiseaseSearch.aspx) and literature.

### Examining the Kuwaiti exomes for rare, deleterious and pathogenic variants

‘Known’ missense and LoF SNVs having MAF of <1% in the 1KGP phase 3 data^4^ and ExAC database^8^ were catalogued. Of these, only the variants annotated as damaging by both SIFT^87^ and PolyPhen-2^88^ tools were retained. The Kuwaiti exomes were examined for such variants. As an additional step, the resulting variants were filtered based on their Combined Annotation-Dependent Depletion score^89^ to prioritise functional, deleterious and pathogenic variants across many functional categories, effect sizes and genetic architectures. A scaled score of ≥20 was applied to retrieve only those variants that were predicted to be among the top 1% of deleterious variants in the human genome. The above set of variants were screened for clinical significance using the OMIM^85^ and ClinVar^80^ databases.

### Variants found to be ‘rare’ within global populations but ‘common’ within Kuwaiti population

A data set of SNVs that are rare in 1KGP phase 3 populations but common within Kuwaiti exomes was created. For such missense variants, scores predicting their pathogenicity were calculating using the REVEL^90^ software.

### Examining the pharmacogenomic relevance of Kuwaiti exome variants

Variants of pharmacogenomic relevance were delineated using the resources built upon the concept of druggable genome originally formulated by Hopkins and Groom^49^ and PharmGKB^50^. From the data set of variants derived for Kuwaiti exomes, missense SNVs (with MAF of >1%) that are not deleterious (i.e. SIFT and PolyPhen-2 scores were outside the deleteriousness range) were mapped to protein domains (using InterPro^91^) and checked for inclusion in the list of 130 domains reported by Hopkins and Groom. From the resulting set of variants mapping to drug-binding domains, only those for which pharmacogenomic annotation was available in PharmGKB database were retained.

## Electronic supplementary material

Supplementary Information

## Data Availability

The 291 individual VCF files were combined to include a genotype (ref/ref, ref/alt, /alt/alt) for each exome. The resulting VCF file containing genotypes for the final variant set of 173,849 SNVs and indels is available online at ftp://dgr.dasmaninstitute.org.
