# Supplementary material for: Assessment of coding region variants in Kuwaiti population: implications for medical genetics and population genomics
Source: Sci Rep. 2018 Nov 8;8:16583. doi: 10.1038/s41598-018-34815-8 (PMC6224454; doi:10.1038/s41598-018-34815-8)
Supplement: Supplementary file 1 — Supplementary Information [file 41598_2018_34815_MOESM1_ESM.docx]

**Assessment of coding region variants in Kuwaiti population: implications for medical genetics and population genomics**

Sumi Elsa John^&^, Dinu Antony^&,1^, Muthukrishnan Eaaswarkhanth, Prashantha Hebbar, Arshad Mohamed Channanath, Daisy Thomas, Sriraman Devarajan, Jaakko Tuomilehto, Fahd Al-Mulla, Osama Alsmadi^*,1^, Thangavel Alphonse Thanaraj^*^

Dasman Diabetes Institute, P.O. Box 1180, Dasman 15462, Kuwait

^&,^ These authors contributed equally to this work and are hence considered as joint first authors.

^*,^ These authors jointly supervised this work. Correspondence may be addressed to either: [**oalsmadi@gmail.com**](mailto:oalsmadi@gmail.com) **(O.A);** [**alphonse.thangavel@dasmaninstitute.org**](mailto:alphonse.thangavel@dasmaninstitute.org) **(T.A.T)**

**^1^**These two authors are currently employed at (DA) Radboud University Medical Center, Nijmegen, The Netherlands, and (OA) King Hussein Cancer Center, Amman, Jordan. Their work components were performed at Dasman Diabetes Institute, Kuwait.

**Supplementary Information**

**List of Supplementary Tables**

**Supplementary Table S1. Distribution of the observed 170,508 SNVs in terms of translated and untranslated region of the coding gene.**

**Supplementary Table S2. Concordance rates in SNP calls between exome sequencing and genome-wide genotyping.**

**Supplementary Table S3. List of homozygous Loss of Function (LoF) variants observed in Kuwaiti exomes.**

**Supplementary Table S4. Functional characterization of the observed 6186 SAFD variants.**

**Supplementary Table S5. The pairwise F_ST_ values calculated (from exonic variants) among Kuwaiti subpopulation groups and population groups from 1000 Genomes project phase3, Qatar and Ashkenazi Jews.**

**Supplementary Table S6. The 85 missense variants that were rare in global populations but common in Kuwaiti Exomes.**

**Supplementary Table S7 – The 230 SAFD variants for which annotation was available in ClinVar.**

**List of Supplementary Figures**

**Supplementary Figure S1. Kuwaiti population subgroup-wide distribution of total number of SNVs upon step-wise addition of exomes.** The solid line represents the number of all variants found as the number of sequenced exomes increase; the dashed line corresponds to population-specific variants. The green corresponds to the KWS subgroup of Saudi Arabian tribe ancestry; orange corresponds to KWP subgroup of Persian ancestry; and the red corresponds to the nomadic Bedouin subgroup of KWB.

**Supplementary Figure S2. The Manhattan plot showing highly significant differentiating variants (labelled red circles) among Kuwaiti subpopulations.** x-axis: chromosome numbers; y-axis: negative log10*pF_ST_* values. Blue line indicates the suggestive line of high significant threshold.

**Supplementary Figure S3. A & B. Scatter plots showing the pairing of Kuwaiti populations with the gnomAD (A) or 1kGP (B) global populations as maximum allele frequency populations.** y-axis: gnomAD (A) or 1kGP (B) gnomAD global population minor allele frequency. (a) x-axis: Overall Kuwaiti population minor allele frequency, (b) x-axis: KWB minor allele frequency; (c) x-axis: KWP minor allele frequency; (d) x-axis: KWS minor allele frequency. gnomAD global populations: AFR – Africans/African Americans, AMR – Admixed Americans, ASJ – Ashkenazi Jewish, EAS – East Asians, FIN – Finnish, NFE – Non-Finnish Europeans, OTH – Other population not assigned, SAS – South Asians. 1kGP global populations: AFR – African, AMR – Admixed American, EAS – East Asian, EUR – European, SAS – South Asian.

**Supplementary Figure S4. Heatmap of mean pairwise *F_ST_* comparison of Kuwaiti subpopulation groups with population groups from 1000 Genomes project phase3, Qatar and Ashkenazi Jews.**

**Supplementary Figure S5. Flowchart illustrating the identification of clinically relevant SAFD variants – considering 1kGP global populations.**

**Supplementary Table S1. Distribution of the observed 170,508 SNVs in terms of translated and untranslated region of the coding gene.**

| **Class** | **Number** | **%** |
| --- | --- | --- |
| 3 prime UTR | 702 | 0.42 |
| 5 prime UTR premature start codon gain | 563 | 0.33 |
| 5 prime UTR | 214 | 0.13 |
| Non-coding exon | 2254 | 1.33 |
| Initiator codon | 330 | 0. 20 |
| Intron | 1097 | 0.65 |
| Splice Acceptor | 70 | 0.04 |
| Splice donor | 50 | 0.03 |
| Splice region | 46 | 0.03 |
| Stop gained | 1425 | 0.84 |
| Stop lost | 95 | 0.06 |
| Stop retained | 50 | 0.03 |
| Missense | 91204 | 53.95 |
| Synonymous | 70955 | 41.97 |
| **Total** | 169055 |  |

**Supplementary Table S2. Concordance rates in SNP calls between exome sequencing and genome-wide genotyping.**

| **Subject** | **#Comparisons** | **#DISCORDANT** | **Concordance Rate** | **HET_DISCORDANT SNPs** | **HOM_DISCORDANT SNPs** |
| --- | --- | --- | --- | --- | --- |
| 395 | 11848 | 29 | 0.99755233 | 29 | 0 |
| 297 | 13074 | 31 | 0.997628882 | 31 | 0 |
| 550 | 12947 | 15 | 0.99884143 | 15 | 0 |
| 371 | 12660 | 13 | 0.998973144 | 13 | 0 |
| 537 | 13039 | 12 | 0.999079684 | 12 | 0 |
| 539 | 12452 | 10 | 0.999196916 | 10 | 0 |
| 531 | 12707 | 10 | 0.999213032 | 10 | 0 |
| 518 | 13272 | 10 | 0.999246534 | 10 | 0 |
| 522 | 13330 | 10 | 0.999249812 | 10 | 0 |
| 487 | 13084 | 9 | 0.999312137 | 9 | 0 |
| 493 | 12521 | 8 | 0.999361073 | 8 | 0 |
| 532 | 12770 | 8 | 0.999373532 | 8 | 0 |
| 524 | 12859 | 8 | 0.999377868 | 8 | 0 |
| 544 | 13102 | 8 | 0.999389406 | 8 | 0 |
| 521 | 13113 | 8 | 0.999389918 | 8 | 0 |
| 489 | 12286 | 7 | 0.999430246 | 7 | 0 |
| 491 | 12297 | 7 | 0.999430755 | 7 | 0 |
| 488 | 12851 | 7 | 0.999455295 | 7 | 0 |
| 495 | 12949 | 7 | 0.999459418 | 7 | 0 |
| 503 | 13061 | 7 | 0.999464053 | 7 | 0 |
| 541 | 13117 | 7 | 0.999466341 | 7 | 0 |
| 551 | 13234 | 7 | 0.999471059 | 7 | 0 |
| 535 | 13303 | 7 | 0.999473803 | 7 | 0 |
| 543 | 13320 | 7 | 0.999474474 | 7 | 0 |
| 438 | 13331 | 7 | 0.999474908 | 7 | 0 |
| 490 | 12604 | 6 | 0.999523961 | 6 | 0 |
| 497 | 12626 | 6 | 0.99952479 | 6 | 0 |
| 405 | 12781 | 6 | 0.999530553 | 6 | 0 |
| 500 | 12793 | 6 | 0.999530994 | 6 | 0 |
| 533 | 12880 | 6 | 0.999534161 | 6 | 0 |
| 4512 | 12901 | 6 | 0.99953492 | 6 | 0 |
| 381 | 13019 | 6 | 0.999539135 | 6 | 0 |
| 545 | 13106 | 6 | 0.999542194 | 6 | 0 |
| 548 | 13203 | 6 | 0.999545558 | 6 | 0 |
| 318 | 13297 | 6 | 0.99954877 | 5 | 1 |
| 547 | 13318 | 6 | 0.999549482 | 6 | 0 |
| 515 | 13319 | 6 | 0.999549516 | 6 | 0 |
| 298 | 13358 | 6 | 0.999550831 | 6 | 0 |
| 160 | 13364 | 6 | 0.999551033 | 6 | 0 |
| 389 | 11973 | 5 | 0.999582394 | 5 | 0 |
| 494 | 12401 | 5 | 0.999596807 | 5 | 0 |
| 397 | 12522 | 5 | 0.999600703 | 5 | 0 |
| 4362 | 12550 | 5 | 0.999601594 | 5 | 0 |
| 377 | 12554 | 5 | 0.999601721 | 5 | 0 |
| 499 | 12760 | 5 | 0.99960815 | 5 | 0 |
| 424 | 13172 | 5 | 0.999620407 | 5 | 0 |
| 486 | 13243 | 5 | 0.999622442 | 5 | 0 |
| 530 | 13247 | 5 | 0.999622556 | 5 | 0 |
| 393 | 13268 | 5 | 0.999623153 | 5 | 0 |
| 422 | 13275 | 5 | 0.999623352 | 5 | 0 |
| 506 | 13292 | 5 | 0.999623834 | 5 | 0 |
| 507 | 13300 | 5 | 0.99962406 | 5 | 0 |
| 407 | 13309 | 5 | 0.999624314 | 5 | 0 |
| 115 | 13310 | 5 | 0.999624343 | 5 | 0 |
| 376 | 13331 | 5 | 0.999624934 | 5 | 0 |
| 154 | 13371 | 5 | 0.999626056 | 5 | 0 |
| 4375 | 12818 | 4 | 0.999687939 | 3 | 1 |
| 498 | 12831 | 4 | 0.999688255 | 4 | 0 |
| 98 | 12911 | 4 | 0.999690187 | 4 | 0 |
| 316 | 12959 | 4 | 0.999691334 | 4 | 0 |
| 527 | 13138 | 4 | 0.99969554 | 4 | 0 |
| 375 | 13144 | 4 | 0.999695679 | 4 | 0 |
| 3272 | 13193 | 4 | 0.999696809 | 4 | 0 |
| 103 | 13233 | 4 | 0.999697725 | 4 | 0 |
| 147 | 13285 | 4 | 0.999698909 | 4 | 0 |
| 427 | 13287 | 4 | 0.999698954 | 4 | 0 |
| 221 | 13294 | 4 | 0.999699112 | 4 | 0 |
| 546 | 13297 | 4 | 0.99969918 | 4 | 0 |
| 284 | 13299 | 4 | 0.999699226 | 4 | 0 |
| 380 | 13304 | 4 | 0.999699339 | 4 | 0 |
| 430 | 13323 | 4 | 0.999699767 | 4 | 0 |
| 505 | 13328 | 4 | 0.99969988 | 4 | 0 |
| 512 | 13349 | 4 | 0.999700352 | 4 | 0 |
| 114 | 13363 | 4 | 0.999700666 | 4 | 0 |
| 485 | 13363 | 4 | 0.999700666 | 4 | 0 |
| 529 | 13366 | 4 | 0.999700733 | 4 | 0 |
| 516 | 13392 | 4 | 0.999701314 | 4 | 0 |
| 188 | 13395 | 4 | 0.999701381 | 4 | 0 |
| 212 | 11697 | 3 | 0.999743524 | 3 | 0 |
| 304 | 11976 | 3 | 0.999749499 | 3 | 0 |
| 492 | 12760 | 3 | 0.99976489 | 3 | 0 |
| 97 | 12796 | 3 | 0.999765552 | 3 | 0 |
| 109 | 12822 | 3 | 0.999766027 | 3 | 0 |
| 138 | 12979 | 3 | 0.999768857 | 3 | 0 |
| 128 | 13044 | 3 | 0.999770009 | 3 | 0 |
| 394 | 13049 | 3 | 0.999770097 | 3 | 0 |
| 100 | 13100 | 3 | 0.999770992 | 3 | 0 |
| 242 | 13108 | 3 | 0.999771132 | 3 | 0 |
| 365 | 13115 | 3 | 0.999771254 | 2 | 1 |
| 549 | 13125 | 3 | 0.999771429 | 3 | 0 |
| 404 | 13175 | 3 | 0.999772296 | 3 | 0 |
| 177 | 13180 | 3 | 0.999772382 | 3 | 0 |
| 185 | 13184 | 3 | 0.999772451 | 3 | 0 |
| 125 | 13197 | 3 | 0.999772676 | 3 | 0 |
| 4154 | 13200 | 3 | 0.999772727 | 3 | 0 |
| 141 | 13213 | 3 | 0.999772951 | 3 | 0 |
| 434 | 13232 | 3 | 0.999773277 | 3 | 0 |
| 501 | 13235 | 3 | 0.999773328 | 3 | 0 |
| 106 | 13242 | 3 | 0.999773448 | 3 | 0 |
| 433 | 13250 | 3 | 0.999773585 | 3 | 0 |
| 525 | 13253 | 3 | 0.999773636 | 3 | 0 |
| 384 | 13287 | 3 | 0.999774215 | 3 | 0 |
| 294 | 13305 | 3 | 0.999774521 | 3 | 0 |
| 542 | 13306 | 3 | 0.999774538 | 3 | 0 |
| 306 | 13309 | 3 | 0.999774589 | 3 | 0 |
| 416 | 13310 | 3 | 0.999774606 | 3 | 0 |
| 250 | 13312 | 3 | 0.999774639 | 3 | 0 |
| 439 | 13312 | 3 | 0.999774639 | 3 | 0 |
| 156 | 13323 | 3 | 0.999774825 | 3 | 0 |
| 167 | 13323 | 3 | 0.999774825 | 2 | 1 |
| 211 | 13335 | 3 | 0.999775028 | 3 | 0 |
| 386 | 13342 | 3 | 0.999775146 | 3 | 0 |
| 300 | 13345 | 3 | 0.999775197 | 3 | 0 |
| 415 | 13348 | 3 | 0.999775247 | 2 | 1 |
| 112 | 13351 | 3 | 0.999775298 | 3 | 0 |
| 161 | 13358 | 3 | 0.999775415 | 3 | 0 |
| 166 | 13363 | 3 | 0.9997755 | 3 | 0 |
| 163 | 13383 | 3 | 0.999775835 | 3 | 0 |
| 534 | 13383 | 3 | 0.999775835 | 3 | 0 |
| 227 | 13410 | 3 | 0.999776286 | 3 | 0 |
| 126 | 12864 | 2 | 0.999844527 | 2 | 0 |
| 164 | 12952 | 2 | 0.999845584 | 2 | 0 |
| 153 | 12978 | 2 | 0.999845893 | 2 | 0 |
| 406 | 13098 | 2 | 0.999847305 | 2 | 0 |
| 426 | 13108 | 2 | 0.999847421 | 2 | 0 |
| 144 | 13142 | 2 | 0.999847816 | 2 | 0 |
| 176 | 13148 | 2 | 0.999847886 | 2 | 0 |
| 183 | 13169 | 2 | 0.999848128 | 2 | 0 |
| 538 | 13195 | 2 | 0.999848427 | 2 | 0 |
| 142 | 13210 | 2 | 0.9998486 | 2 | 0 |
| 132 | 13217 | 2 | 0.99984868 | 2 | 0 |
| 379 | 13233 | 2 | 0.999848863 | 2 | 0 |
| 182 | 13254 | 2 | 0.999849102 | 2 | 0 |
| 366 | 13261 | 2 | 0.999849182 | 2 | 0 |
| 418 | 13268 | 2 | 0.999849261 | 2 | 0 |
| 222 | 13274 | 2 | 0.99984933 | 2 | 0 |
| 178 | 13275 | 2 | 0.999849341 | 2 | 0 |
| 408 | 13281 | 2 | 0.999849409 | 2 | 0 |
| 175 | 13284 | 2 | 0.999849443 | 2 | 0 |
| 437 | 13286 | 2 | 0.999849466 | 2 | 0 |
| 392 | 13287 | 2 | 0.999849477 | 2 | 0 |
| 111 | 13292 | 2 | 0.999849534 | 2 | 0 |
| 536 | 13302 | 2 | 0.999849647 | 2 | 0 |
| 382 | 13303 | 2 | 0.999849658 | 2 | 0 |
| 440 | 13305 | 2 | 0.999849681 | 2 | 0 |
| 421 | 13308 | 2 | 0.999849714 | 2 | 0 |
| 131 | 13310 | 2 | 0.999849737 | 2 | 0 |
| 517 | 13312 | 2 | 0.99984976 | 2 | 0 |
| 513 | 13314 | 2 | 0.999849782 | 2 | 0 |
| 127 | 13317 | 2 | 0.999849816 | 2 | 0 |
| 225 | 13321 | 2 | 0.999849861 | 2 | 0 |
| 417 | 13322 | 2 | 0.999849872 | 2 | 0 |
| 428 | 13322 | 2 | 0.999849872 | 2 | 0 |
| 431 | 13327 | 2 | 0.999849929 | 2 | 0 |
| 412 | 13335 | 2 | 0.999850019 | 2 | 0 |
| 369 | 13343 | 2 | 0.999850109 | 2 | 0 |
| 504 | 13347 | 2 | 0.999850154 | 2 | 0 |
| 173 | 13352 | 2 | 0.99985021 | 2 | 0 |
| 145 | 13355 | 2 | 0.999850243 | 2 | 0 |
| 289 | 13357 | 2 | 0.999850266 | 2 | 0 |
| 511 | 13357 | 2 | 0.999850266 | 2 | 0 |
| 150 | 13359 | 2 | 0.999850288 | 2 | 0 |
| 301 | 13359 | 2 | 0.999850288 | 2 | 0 |
| 296 | 13360 | 2 | 0.999850299 | 2 | 0 |
| 368 | 13361 | 2 | 0.999850311 | 2 | 0 |
| 540 | 13363 | 2 | 0.999850333 | 2 | 0 |
| 231 | 13365 | 2 | 0.999850355 | 2 | 0 |
| 110 | 13366 | 2 | 0.999850367 | 2 | 0 |
| 273 | 13366 | 2 | 0.999850367 | 2 | 0 |
| 520 | 13370 | 2 | 0.999850411 | 2 | 0 |
| 265 | 13374 | 2 | 0.999850456 | 2 | 0 |
| 148 | 13382 | 2 | 0.999850546 | 2 | 0 |
| 190 | 13383 | 2 | 0.999850557 | 2 | 0 |
| 215 | 13386 | 2 | 0.99985059 | 2 | 0 |
| 168 | 13391 | 2 | 0.999850646 | 2 | 0 |
| 230 | 13394 | 2 | 0.999850679 | 2 | 0 |
| 189 | 13399 | 2 | 0.999850735 | 2 | 0 |
| 236 | 13401 | 2 | 0.999850757 | 2 | 0 |
| 223 | 13427 | 2 | 0.999851046 | 2 | 0 |
| 207 | 11375 | 1 | 0.999912088 | 1 | 0 |
| 171 | 12794 | 1 | 0.999921838 | 1 | 0 |
| 206 | 12941 | 1 | 0.999922726 | 1 | 0 |
| 203 | 12970 | 1 | 0.999922899 | 1 | 0 |
| 3636 | 13025 | 1 | 0.999923225 | 1 | 0 |
| 383 | 13030 | 1 | 0.999923254 | 1 | 0 |
| 4363 | 13077 | 1 | 0.99992353 | 1 | 0 |
| 209 | 13150 | 1 | 0.999923954 | 1 | 0 |
| 134 | 13161 | 1 | 0.999924018 | 1 | 0 |
| 312 | 13166 | 1 | 0.999924047 | 1 | 0 |
| 101 | 13192 | 1 | 0.999924196 | 1 | 0 |
| 496 | 13194 | 1 | 0.999924208 | 1 | 0 |
| 213 | 13201 | 1 | 0.999924248 | 1 | 0 |
| 123 | 13205 | 1 | 0.999924271 | 1 | 0 |
| 3012 | 13225 | 1 | 0.999924386 | 1 | 0 |
| 130 | 13228 | 1 | 0.999924403 | 1 | 0 |
| 390 | 13234 | 1 | 0.999924437 | 1 | 0 |
| 429 | 13251 | 1 | 0.999924534 | 1 | 0 |
| 425 | 13272 | 1 | 0.999924653 | 1 | 0 |
| 370 | 13274 | 1 | 0.999924665 | 1 | 0 |
| 409 | 13277 | 1 | 0.999924682 | 1 | 0 |
| 174 | 13288 | 1 | 0.999924744 | 1 | 0 |
| 526 | 13293 | 1 | 0.999924772 | 1 | 0 |
| 311 | 13296 | 1 | 0.999924789 | 1 | 0 |
| 172 | 13299 | 1 | 0.999924806 | 1 | 0 |
| 116 | 13301 | 1 | 0.999924818 | 1 | 0 |
| 180 | 13304 | 1 | 0.999924835 | 1 | 0 |
| 413 | 13305 | 1 | 0.99992484 | 1 | 0 |
| 107 | 13307 | 1 | 0.999924852 | 1 | 0 |
| 165 | 13307 | 1 | 0.999924852 | 1 | 0 |
| 372 | 13310 | 1 | 0.999924869 | 1 | 0 |
| 152 | 13311 | 1 | 0.999924874 | 1 | 0 |
| 117 | 13314 | 1 | 0.999924891 | 1 | 0 |
| 293 | 13315 | 1 | 0.999924897 | 1 | 0 |
| 292 | 13318 | 1 | 0.999924914 | 1 | 0 |
| 246 | 13323 | 1 | 0.999924942 | 1 | 0 |
| 118 | 13324 | 1 | 0.999924947 | 1 | 0 |
| 414 | 13326 | 1 | 0.999924959 | 1 | 0 |
| 220 | 13329 | 1 | 0.999924976 | 1 | 0 |
| 104 | 13331 | 1 | 0.999924987 | 1 | 0 |
| 149 | 13331 | 1 | 0.999924987 | 1 | 0 |
| 396 | 13331 | 1 | 0.999924987 | 1 | 0 |
| 119 | 13337 | 1 | 0.999925021 | 1 | 0 |
| 139 | 13338 | 1 | 0.999925026 | 1 | 0 |
| 282 | 13361 | 1 | 0.999925155 | 1 | 0 |
| 502 | 13362 | 1 | 0.999925161 | 1 | 0 |
| 281 | 13363 | 1 | 0.999925167 | 1 | 0 |
| 299 | 13370 | 1 | 0.999925206 | 1 | 0 |
| 262 | 13371 | 1 | 0.999925211 | 0 | 1 |
| 302 | 13371 | 1 | 0.999925211 | 1 | 0 |
| 155 | 13373 | 1 | 0.999925222 | 1 | 0 |
| 264 | 13373 | 1 | 0.999925222 | 1 | 0 |
| 162 | 13375 | 1 | 0.999925234 | 1 | 0 |
| 523 | 13377 | 1 | 0.999925245 | 1 | 0 |
| 151 | 13379 | 1 | 0.999925256 | 1 | 0 |
| 280 | 13383 | 1 | 0.999925278 | 1 | 0 |
| 274 | 13385 | 1 | 0.99992529 | 1 | 0 |
| 528 | 13394 | 1 | 0.99992534 | 1 | 0 |
| 228 | 13413 | 1 | 0.999925445 | 1 | 0 |
| 233 | 13429 | 1 | 0.999925534 | 1 | 0 |
| 66 | 12956 | 0 | 1 | 0 | 0 |
| 120 | 13322 | 0 | 1 | 0 | 0 |
| 122 | 13384 | 0 | 1 | 0 | 0 |
| 133 | 13246 | 0 | 1 | 0 | 0 |
| 136 | 13182 | 0 | 1 | 0 | 0 |
| 157 | 13375 | 0 | 1 | 0 | 0 |
| 158 | 13336 | 0 | 1 | 0 | 0 |
| 159 | 13349 | 0 | 1 | 0 | 0 |
| 184 | 13331 | 0 | 1 | 0 | 0 |
| 208 | 11432 | 0 | 1 | 0 | 0 |
| 217 | 13402 | 0 | 1 | 0 | 0 |
| 224 | 13415 | 0 | 1 | 0 | 0 |
| 229 | 13430 | 0 | 1 | 0 | 0 |
| 234 | 13365 | 0 | 1 | 0 | 0 |
| 238 | 13411 | 0 | 1 | 0 | 0 |
| 254 | 13218 | 0 | 1 | 0 | 0 |
| 277 | 13328 | 0 | 1 | 0 | 0 |
| 285 | 13387 | 0 | 1 | 0 | 0 |
| 305 | 13100 | 0 | 1 | 0 | 0 |
| 310 | 13032 | 0 | 1 | 0 | 0 |
| 313 | 13146 | 0 | 1 | 0 | 0 |
| 314 | 13203 | 0 | 1 | 0 | 0 |
| 315 | 13218 | 0 | 1 | 0 | 0 |
| 367 | 13349 | 0 | 1 | 0 | 0 |
| 378 | 13207 | 0 | 1 | 0 | 0 |
| 398 | 13337 | 0 | 1 | 0 | 0 |
| 510 | 13357 | 0 | 1 | 0 | 0 |
| 519 | 13361 | 0 | 1 | 0 | 0 |
| 2164 | 13123 | 0 | 1 | 0 | 0 |
| 3100 | 13064 | 0 | 1 | 0 | 0 |

**Supplementary Table S3. List of homozygous Loss of Function (LoF) variants observed in Kuwaiti exomes.**

| **Chr** | **Position** | **Identifier** | **Gene Names** | **Ref** | **Alt** | **GME_AF** | **1kG_MAF** | **KW_MAF** |
| --- | --- | --- | --- | --- | --- | --- | --- | --- |
| 1 | 19597370 | rs115063329 | ? | G | A | . | 0.009185 | 0.006873 |
| 1 | 20501582 | rs12139100 | PLA2G2C | G | A | 0.236153 | 0.240216 | 0.2543 |
| 1 | 26508997 | rs2802347 | CNKSR1 | C | T | 0.017172 | 0.051717 | 0.02234 |
| 1 | 40773150 | rs12077871 | COL9A2 | G | A | 0.023162 | 0.063898 | 0.02234 |
| 1 | 47080679 | rs6671527 | MKNK1,MOB3C | G | A | 0.550856 | 0.642173 | 0.5687 |
| 1 | 48708228 | rs850763 | SLC5A9 | G | T | 0.195871 | 0.113818 | 0.2045 |
| 1 | 55182300 | rs1147990 | MROH7-TTC4,TTC4 | T | A | 0.572149 | 0.668331 | 0.5704 |
| 1 | 100316589 | rs2307130 | AGL | A | G | . | 0.427117 | 0.323 |
| 1 | 110775470 | rs370593032 | KCNC4 | G | A | . | 0.0002 | 0.003436 |
| 1 | 115137133 | . | DENND2C | G | A | . | . | 0.008591 |
| 1 | 151740657 | rs367796164 | OAZ3 | C | T | 0.005035 | . | 0.008591 |
| 1 | 152323132 | rs12568784 | FLG2 | G | T | 0.271903 | 0.316893 | 0.2543 |
| 1 | 156347131 | rs2245623 | RHBG | G | A | 0.376133 | 0.270567 | 0.3608 |
| 1 | 157772404 | rs12078586 | FCRL1 | C | T | 0.012085 | 0.055312 | 0.03608 |
| 1 | 158549492 | rs863362 | OR10X1 | C | T | 0.481873 | 0.502396 | 0.4674 |
| 1 | 159410340 | rs12409540 | OR10J1 | T | A | 0.096173 | 0.084465 | 0.0945 |
| 1 | 159785370 | rs4301626 | FCRL6 | T | C | 0.186808 | 0.191693 | 0.2131 |
| 1 | 159785413 | rs61823162 | FCRL6 | C | T | 0.094663 | 0.057708 | 0.06357 |
| 1 | 161476204 | rs9427397 | FCGR2A | C | T | 0.141994 | 0.05611 | 0.1598 |
| 1 | 223285200 | rs5744168 | TLR5 | G | A | 0.053877 | 0.05032 | 0.04467 |
| 1 | 235324545 | rs66929873 | ARID4B,RBM34 | T | A | . | 0.112021 | 0.1306 |
| 1 | 236706300 | rs2273865 | LGALS8 | T | A | 0.127895 | 0.13119 | 0.09966 |
| 2 | 26700289 | rs75624587 | OTOF | C | A | 0.019757 | 0.034545 | 0.02062 |
| 2 | 108863758 | rs112050262 | SULT1C3 | G | A | 0.026687 | 0.01877 | 0.02234 |
| 2 | 176965359 | rs200878101 | HOXD12 | C | T | 0.035247 | 0.003195 | 0.07216 |
| 2 | 228228620 | rs774704649 | TM4SF20 | C | T | . | . | 0.003436 |
| 3 | 32030998 | rs4639011 | OSBPL10,ZNF860 | C | T | 0.056898 | 0.08147 | 0.05498 |
| 3 | 52476604 | . | SEMA3G | C | A | . | . | 0.003436 |
| 3 | 53899276 | rs1043261 | IL17RB | C | T | 0.09718 | 0.135184 | 0.09107 |
| 3 | 69154058 | rs186177589 | ARL6IP5 | T | C | . | 0.001398 | 0.0378 |
| 3 | 113955187 | rs3732781 | ZNF80 | A | C | . | 0.185503 | 0.3385 |
| 3 | 119222456 | rs1131265 | TIMMDC1 | G | C | 0.184662 | 0.207268 | 0.1718 |
| 3 | 129152089 | rs140696 | MBD4 | G | A | 0.176737 | 0.167332 | 0.146 |
| 3 | 149245675 | rs28763914 | WWTR1 | T | A | 0.019134 | 0.019569 | 0.02405 |
| 3 | 183754294 | rs6443930 | HTR3D | G | C | 0.534743 | 0.533746 | 0.5 |
| 3 | 194061907 | rs4974539 | CPN2 | G | A | 0.282981 | 0.280152 | 0.2818 |
| 4 | 1090625 | rs4045481 | RNF212 | G | A | 0.641994 | 0.546725 | 0.5945 |
| 4 | 15835844 | rs1800051 | CD38 | A | C | 0.084089 | 0.08746 | 0.07732 |
| 4 | 70512787 | rs111696697 | UGT2A1 | A | T | 0.047331 | 0.014577 | 0.0378 |
| 4 | 84206004 | rs112033303 | COQ2 | T | A | . | 0.009984 | 0.02577 |
| 4 | 130030763 | rs10009430 | C4orf33 | C | T | 0.004532 | 0.020567 | 0.01375 |
| 4 | 144918712 | rs1132783 | GYPB | C | G | 0.270393 | 0.176118 | 0.2904 |
| 5 | 2755485 | rs62333235 | C5orf38 | C | T | . | 0.296725 | 0.378 |
| 5 | 41061715 | rs1023840 | MROH2B | C | T | 0.170695 | 0.208267 | 0.1684 |
| 5 | 56212649 | rs538386345 | SETD9 | C | T | . | 0.0002 | 0.005155 |
| 5 | 94749787 | rs35391433 | FAM81B | C | T | 0.059919 | 0.013978 | 0.06014 |
| 6 | 26446011 | rs138065289 | BTN3A3 | G | A | 0.017442 | 0.003794 | 0.02921 |
| 6 | 36274148 | rs45621032 | PNPLA1 | T | A | 0.012588 | 0.006989 | 0.01031 |
| 6 | 150387059 | rs34672740 | ULBP3 | C | A | . | 0.01877 | 0.005155 |
| 6 | 158571611 | rs112780453 | SERAC1 | A | T | 0.033838 | 0.010982 | 0.03608 |
| 6 | 167709702 | rs2235197 | UNC93A | G | A | 0.105237 | 0.110423 | 0.09107 |
| 6 | 168709385 | rs6925614 | DACT2 | T | C | 0.469311 | 0.3752 | 0.4021 |
| 7 | 966260 | rs142995759 | ADAP1,COX19 | G | A | 0.010846 | 0.001797 | 0.02749 |
| 7 | 2752059 | rs55919423 | AMZ1 | C | T | . | 0.010184 | 0.02749 |
| 7 | 12414725 | rs17165936 | VWDE | G | A | 0.101737 | 0.166733 | 0.1151 |
| 7 | 21582963 | rs2285943 | DNAH11 | G | T | 0.497625 | 0.411941 | 0.4914 |
| 7 | 23757162 | rs6945306 | STK31 | G | C | 0.36858 | . | 0.3522 |
| 7 | 34889222 | rs10275028 | NPSR1 | T | C | 0.339879 | 0.267173 | 0.3505 |
| 7 | 57194328 | rs762992236 | ZNF479 | A | T | . | . | 0.003436 |
| 7 | 127250907 | rs712700 | PAX4 | T | C | . | 0.669329 | 0.7732 |
| 7 | 149983566 | rs181975624 | ACTR3C | G | A | 0.069486 | 0.032748 | 0.08419 |
| 8 | 23147564 | rs3808536 | R3HCC1 | G | A | . | 0.532748 | 0.5601 |
| 8 | 39872935 | rs4503083 | IDO2 | T | A | 0.233132 | 0.229832 | 0.2371 |
| 8 | 52284560 | rs117752382 | PXDNL | A | T | 0.050856 | 0.014177 | 0.07388 |
| 8 | 95906128 | rs35528614 | CCNE2 | A | G | 0.002518 | 0.004593 | 0.006873 |
| 8 | 134239770 | rs3739261 | WISP1 | T | C | 0.296073 | 0.278754 | 0.2852 |
| 9 | 125391241 | rs1476860 | OR1B1 | G | A | 0.269889 | 0.339257 | 0.2801 |
| 9 | 136083580 | rs2073870 | OBP2B | T | G | 0.828542 | 0.73722 | 0.7973 |
| 9 | 139235526 | rs59873903 | GPSM1 | C | A | 0.165598 | 0.223243 | 0.1082 |
| 9 | 139634495 | rs9886752 | LCN6,LCN10 | G | A | 0.22004 | 0.199281 | 0.2165 |
| 10 | 1065710 | rs1044261 | GTPBP4,IDI2 | C | T | 0.074522 | 0.030551 | 0.06014 |
| 10 | 4889403 | rs12240276 | AKR1E2 | C | T | 0.090131 | 0.078474 | 0.1598 |
| 10 | 50339945 | rs561798621 | FAM170B | C | A | . | 0.0002 | 0.005155 |
| 10 | 88722398 | rs9864 | MMRN2,SNCG | A | T | 0.225076 | 0.203674 | 0.2165 |
| 10 | 95082882 | rs34048939 | MYOF | G | A | 0.083082 | 0.027955 | 0.1254 |
| 10 | 97763935 | . | CC2D2B,RP11-690P14.4 | C | G | . | . | 0.003436 |
| 10 | 124214355 | rs2736911 | ARMS2 | C | T | 0.079557 | 0.114417 | 0.07216 |
| 11 | 5444136 | rs2647574 | HBE1,HBG2,OR51Q1 | C | T | 0.388218 | 0.453874 | 0.4055 |
| 11 | 5462702 | rs16930998 | HBE1,HBG2,OR51I1 | G | A | 0.052367 | 0.128395 | 0.07216 |
| 11 | 5776484 | rs4910844 | OR52N4,TRIM5 | A | T | 0.285498 | 0.214457 | 0.3213 |
| 11 | 7712471 | rs4509745 | ? | C | T | 0.428931 | 0.479832 | 0.4296 |
| 11 | 33106616 | rs3758741 | CSTF3,TCP11L1 | C | T | . | 0.229034 | 0.3711 |
| 11 | 47306630 | rs35233100 | MADD | C | T | 0.031722 | 0.016973 | 0.03952 |
| 11 | 48286231 | rs10838851 | OR4X1 | T | A | 0.642497 | 0.604233 | 0.6065 |
| 11 | 55861650 | rs61887097 | OR8I2 | C | G | 0.046875 | 0.038938 | 0.03952 |
| 11 | 56086560 | rs117366703 | OR8K3 | C | T | 0.02719 | 0.00619 | 0.01546 |
| 11 | 56310356 | rs17547284 | OR5M11 | A | T | 0.05287 | 0.042732 | 0.05155 |
| 11 | 56431216 | rs11228710 | OR5AR1 | C | T | 0.628902 | 0.623003 | 0.5412 |
| 11 | 59245593 | rs75898556 | OR4D10 | G | T | 0.023162 | 0.077077 | 0.02921 |
| 11 | 59480952 | rs499037 | OR10V1,STX3 | G | A | 0.015106 | 0.063698 | 0.04296 |
| 11 | 60265002 | rs2298553 | MS4A12 | C | T | 0.472306 | 0.478235 | 0.5086 |
| 11 | 61165741 | rs10897158 | TMEM216 | G | C | 0.826284 | 0.719649 | 0.7646 |
| 11 | 62848487 | rs11231341 | SLC22A24 | A | C | 0.797424 | 0.753994 | 0.7801 |
| 11 | 62951221 | rs11231397 | SLC22A10,SLC22A25 | C | G | 0.463243 | 0.420327 | 0.4811 |
| 11 | 63057925 | rs1790218 | SLC22A10 | G | A | 0.468781 | 0.433506 | 0.39 |
| 11 | 65545913 | rs56798161 | AP5B1 | G | A | 0.014706 | 0.030951 | 0.01718 |
| 11 | 70229171 | rs552282 | PPFIA1 | G | A | . | 0.413339 | 0.3557 |
| 11 | 70279766 | rs643301 | CTTN | C | T | 0.21148 | 0.183107 | 0.244 |
| 11 | 73978243 | rs3741132 | P4HA3 | G | A | . | 0.226637 | 0.1203 |
| 11 | 74563077 | rs2304683 | XRRA1 | G | A | 0.12286 | 0.267572 | 0.08935 |
| 11 | 124121199 | rs4268525 | ? | G | C | 0.465257 | 0.444688 | 0.4433 |
| 12 | 1022569 | rs4987208 | RAD52 | A | C | 0.011078 | 0.022165 | 0.01375 |
| 12 | 1023218 | rs4987207 | RAD52 | G | T | 0.028701 | 0.021765 | 0.03952 |
| 12 | 7475081 | rs7485773 | ACSM4 | C | T | 0.066465 | 0.048522 | 0.0756 |
| 12 | 7805414 | rs34275479 | APOBEC1 | C | T | . | 0.002196 | 0.003436 |
| 12 | 10271087 | rs16910526 | CLEC7A | A | C | 0.067976 | 0.040935 | 0.06701 |
| 12 | 11214145 | rs2708381 | PRR4,TAS2R14,TAS2R46 | C | T | 0.196878 | 0.211861 | 0.189 |
| 12 | 55641255 | rs4522268 | OR6C74 | C | T | 0.365055 | 0.235623 | 0.3454 |
| 12 | 56079053 | rs139136931 | ITGA7 | G | A | 0.001007 | 0.000799 | 0.005155 |
| 12 | 113448288 | rs15895 | OAS2 | A | G | 0.672205 | 0.864417 | 0.6443 |
| 13 | 100518634 | rs41281112 | CLYBL | C | T | 0.024169 | 0.021965 | 0.04811 |
| 14 | 21026773 | rs891297 | RNASE9 | C | T | . | 0.313698 | 0.2423 |
| 14 | 25103414 | rs2273844 | GZMB | G | A | . | 0.295927 | 0.2938 |
| 14 | 54417522 | rs17563 | BMP4 | A | G | 0.377644 | 0.325679 | 0.3814 |
| 14 | 64560092 | rs2781377 | ESR2,SYNE2 | G | A | 0.119335 | 0.122404 | 0.09622 |
| 14 | 73989211 | rs149734041 | HEATR4 | G | A | 0.00856 | . | 0.04296 |
| 14 | 75159007 | rs2270424 | AC007956.1,AREL1 | G | A | . | 0.244609 | 0.1821 |
| 14 | 88862529 | rs3179969 | SPATA7 | G | A | 0.282038 | 0.407548 | 0.2698 |
| 14 | 100384160 | rs34820758 | EML1 | G | A | 0.004028 | 0.002995 | 0.006873 |
| 14 | 102729886 | rs34931752 | MOK | G | A | 0.061996 | 0.050719 | 0.0567 |
| 15 | 31294714 | rs3784589 | TRPM1 | C | A | 0.090131 | 0.068291 | 0.1065 |
| 15 | 43658935 | rs3917221 | ZSCAN29 | C | T | 0.256798 | 0.134984 | 0.2526 |
| 15 | 55722882 | rs57809907 | DYX1C1 | C | A | 0.122356 | 0.175519 | 0.1392 |
| 15 | 68497597 | rs11071990 | CALML4,RP11-315D16.2 | G | A | 0.036757 | 0.084465 | 0.02405 |
| 15 | 72639003 | rs1800430 | HEXA | T | C | 0.007553 | 0.036741 | 0.02921 |
| 15 | 78841220 | rs8053 | PSMA4 | T | C | 0.71148 | 0.752396 | 0.677 |
| 15 | 90764219 | rs4932305 | SEMA4B | G | A | 0.142785 | 0.276957 | 0.1581 |
| 15 | 97327393 | rs3812907 | SPATA8 | C | T | 0.092649 | 0.115216 | 0.08935 |
| 16 | 334920 | rs45619835 | PDIA2 | C | T | 0.02568 | 0.004593 | 0.01375 |
| 16 | 4847841 | rs75818610 | ROGDI | G | A | 0.000504 | 0.008586 | 0.003436 |
| 16 | 13297348 | rs12444395 | SHISA9 | C | T | 0.023419 | 0.015974 | 0.02749 |
| 16 | 20638576 | rs1692729 | ACSM1,ACSM3 | A | T | 0.496475 | 0.605831 | 0.5567 |
| 16 | 70694000 | rs4985556 | IL34 | C | A | 0.055891 | 0.059904 | 0.03608 |
| 16 | 71264561 | rs3743953 | HYDIN | A | G | 0.109459 | 0.163738 | 0.07045 |
| 16 | 81056441 | rs3743503 | CENPN | T | G | 0.090222 | 0.132588 | 0.08935 |
| 16 | 81199544 | rs12925771 | ? | G | A | 0.105372 | 0.387181 | 0.2423 |
| 16 | 81242198 | rs7499011 | ? | G | A | 0.391239 | 0.161741 | 0.3918 |
| 16 | 90097672 | rs141063112 | GAS8 | C | A | . | 0.009585 | 0.01718 |
| 17 | 4461748 | rs7215121 | GGT6 | G | A | 0.520263 | 0.496805 | 0.5034 |
| 17 | 4803711 | rs35400274 | C17orf107,CHRNE | G | A | 0.243982 | 0.221645 | 0.2388 |
| 17 | 14005439 | rs2159132 | COX10 | G | A | 0.601208 | 0.491813 | 0.567 |
| 17 | 19578873 | rs7216 | ALDH3A2 | A | T | 0.528701 | 0.678514 | 0.4725 |
| 17 | 38519831 | rs141158879 | CTD-2267D19.3,GJD3 | G | T | 0.014199 | 0.005791 | 0.01375 |
| 17 | 39643659 | rs145372311 | KRT36 | C | A | 0.022155 | 0.002396 | 0.05155 |
| 17 | 45468858 | rs118004742 | EFCAB13 | T | G | 0.109265 | 0.023163 | 0.1323 |
| 17 | 59667953 | rs17610181 | NACA2 | G | A | 0.213998 | 0.147963 | 0.1512 |
| 17 | 72443138 | rs139362972 | GPRC5C | G | A | 0.021148 | 0.000799 | 0.0378 |
| 17 | 72588806 | rs545652 | C17orf77 | C | A | 0.173212 | 0.223642 | 0.2216 |
| 17 | 78166385 | rs11658460 | CARD14 | C | T | 0.122356 | 0.121406 | 0.09794 |
| 18 | 51880889 | rs17292725 | STARD6 | G | A | 0.057402 | 0.014577 | 0.0756 |
| 19 | 10132000 | rs77833898 | RDH8 | G | A | 0.145015 | 0.073083 | 0.1581 |
| 19 | 12224172 | rs8109273 | ZNF20,ZNF788 | T | C | . | 0.19349 | 0.1564 |
| 19 | 12540971 | rs28559848 | CTD-3105H18.16,ZNF443 | T | A | 0.305614 | 0.276757 | 0.3574 |
| 19 | 15789140 | rs609290 | CYP4F12 | A | G | 0.935549 | 0.81869 | 0.8986 |
| 19 | 35719020 | rs541169 | FAM187B | C | T | 0.414904 | 0.318291 | 0.4313 |
| 19 | 36616366 | rs775648395 | TBCB | C | G | . | . | 0.005155 |
| 19 | 38385370 | rs779995073 | WDR87 | C | A | 0.002457 | . | 0.003436 |
| 19 | 39224413 | rs4801861 | CAPN12 | A | G | 0.801411 | 0.801118 | 0.7904 |
| 19 | 43519362 | rs11083680 | PSG11 | C | T | 0.478702 | 0.647564 | 0.4278 |
| 19 | 48737706 | rs2043211 | CARD8,ZNF114 | A | T | 0.331653 | 0.316494 | 0.3093 |
| 19 | 49206674 | rs601338 | FUT2 | G | A | 0.501008 | 0.321685 | 0.4828 |
| 19 | 49442735 | rs2270940 | DHDH | C | T | 0.065962 | 0.083467 | 0.05155 |
| 19 | 49445774 | rs10423255 | DHDH | C | T | 0.04458 | 0.055112 | 0.04467 |
| 19 | 52004903 | rs16982743 | SIGLEC12 | G | A | 0.258308 | 0.185903 | 0.2079 |
| 19 | 55019261 | rs61737751 | LAIR2 | C | T | 0.046324 | 0.030152 | 0.04811 |
| 19 | 56499279 | rs306457 | NLRP8 | G | C | 0.700403 | 0.724641 | 0.6821 |
| 20 | 1294073 | rs141976631 | SDCBP2 | G | A | 0.008105 | 0.001797 | 0.006873 |
| 20 | 3672664 | . | SIGLEC1 | G | A | . | . | 0.003436 |
| 20 | 20033242 | rs2273057 | C20orf26,CRNKL1 | A | T | 0.488911 | 0.51897 | 0.4983 |
| 20 | 29976830 | rs12329612 | DEFB119 | A | C | 0.022681 | 0.041733 | 0.0189 |
| 21 | 31744127 | rs877346 | KRTAP13-2 | A | T | 0.398288 | 0.318091 | 0.3127 |
| 21 | 40552307 | rs3171465 | PSMG1 | A | G | 0.472306 | 0.473842 | 0.4691 |
| 21 | 44323720 | rs4148974 | NDUFV3 | C | T | 0.048842 | 0.052316 | 0.04639 |
| 21 | 45725746 | rs118169148 | PFKL | C | T | . | 0.017372 | 0.02405 |
| 22 | 17469049 | rs28502153 | GAB4 | C | A | 0.376518 | 0.353634 | 0.3608 |
| 22 | 18912677 | rs11913840 | PRODH | C | T | 0.109768 | 0.05631 | 0.05842 |
| 22 | 32643460 | rs62239058 | SLC5A4 | C | A | 0.012085 | 0.011382 | 0.02062 |
| X | 2799129 | . | GYG2 | G | T | . | . | 0.003436 |
| X | 38146346 | . | RPGR,TM4SF2 | C | T | 0.000688 | . | 0.003436 |
| X | 46360423 | rs182004761 | ZNF674 | G | A | 0.004152 | 0.001589 | 0.003436 |
| X | 66941746 | . | AR | G | A | . | . | 0.003436 |
| X | 75004529 | rs1343879 | MAGEE2 | C | A | 0.042837 | 0.275497 | 0.03608 |
| X | 84349207 | rs201698668 | SATL1 | G | C | 0.010366 | 0.001854 | 0.02749 |
| X | 130678702 | rs202199732 | OR13H1 | C | T | 0.001377 | 0.004503 | 0.003436 |
| X | 134156150 | rs756077296 | FAM127C | A | C | 0.001448 | . | 0.003436 |
| X | 150349478 | . | GPR50 | A | T | . | . | 0.003436 |

**Supplementary Table S4. Functional characterization of the observed 6186 SAFD variants.**

|  | **All variants** | **All NON-POPULATION-SPECIFIC variants** | | **SAFD variants** | |
| --- | --- | --- | --- | --- | --- |
|  | **Number of variants** | **Number of variants** | **Average variants per individual** | **Number of variants** | **Average variants per individual** |
| **SNVs** | 170508 | 114896 | NA | 6186 | 1215 |
| **Ti:Tv** | 3.22 | NA | NA | 3.56 | 3.55 |
| **Personal** | 82725 | 34896 | NA | 0 | 0 |
| **Missense SNVs** | 91204 | 57700 | NA | 2960 | 528 |
| **Synonymous SNVs** | 70955 | 51796 | NA | 2913 | 622 |
| **Stop gain SNVs** | 1425 | 704 | NA | 20 | 4 |
| **Stop loss SNVs** | 95 | 73 | NA | 2 | 1 |
| **LoF SNVs** | 2883 | 1071 | NA | 26 | 3 |

**Supplementary Table S5. The pairwise *F*_ST_ values calculated (from exonic variants) among Kuwaiti subpopulation groups and population groups from 1000 Genomes project phase3, Qatar and Ashkenazi Jews.**

| **Population** | **Admixed American** | **African** | **Ashkenazi Jews** | **East Asian** | **European** | **KWB** | **KWP** | **KWS** | **Qatari** | **South Asian** |
| --- | --- | --- | --- | --- | --- | --- | --- | --- | --- | --- |
| **AdmixedAmerican** | 0.0000 | 0.0432 | 0.0192 | 0.0375 | 0.0158 | 0.0208 | 0.0187 | 0.0242 | 0.0204 | 0.0233 |
| **African** | 0.0432 | 0.0000 | 0.0460 | 0.0539 | 0.0465 | 0.0388 | 0.0497 | 0.0526 | 0.0409 | 0.0422 |
| **AshkenaziJewish** | 0.0192 | 0.0460 | 0.0000 | 0.0506 | 0.0080 | 0.0103 | 0.0071 | 0.0104 | 0.0102 | 0.0234 |
| **EastAsian** | 0.0375 | 0.0539 | 0.0506 | 0.0000 | 0.0488 | 0.0745 | 0.0594 | 0.0647 | 0.0592 | 0.0370 |
| **European** | 0.0158 | 0.0465 | 0.0080 | 0.0488 | 0.0000 | 0.0143 | 0.0093 | 0.0155 | 0.0135 | 0.0216 |
| **KuwaitiB** | 0.0208 | 0.0388 | 0.0103 | 0.0745 | 0.0143 | 0.0000 | 0.0038 | 0.0040 | 0.0005 | 0.0202 |
| **KuwaitiP** | 0.0187 | 0.0497 | 0.0071 | 0.0594 | 0.0093 | 0.0038 | 0.0000 | 0.0039 | 0.0027 | 0.0155 |
| **KuwaitiS** | 0.0242 | 0.0526 | 0.0104 | 0.0647 | 0.0155 | 0.0040 | 0.0039 | 0.0000 | 0.0023 | 0.0245 |
| **Qatari** | 0.0204 | 0.0409 | 0.0102 | 0.0592 | 0.0135 | 0.0005 | 0.0027 | 0.0023 | 0.0000 | 0.0183 |
| **SouthAsian** | 0.0233 | 0.0422 | 0.0234 | 0.0370 | 0.0216 | 0.0202 | 0.0155 | 0.0245 | 0.0183 | 0.0000 |

**Supplementary Table S6. The 85 missense variants that were rare in global populations but common in Kuwaiti Exomes.**

| **Chr:**  **Position** | **dbSNP (ClinVar annotation for pathogenicity, where available)** | **Ref_Alt** | **Gene** | **KW_**  **MAF** | **1kGP_**  **MAF** | **Ratio**  **KW_1KGP** | **REVEL score** | **OMIM_annotation for the gene harboring the variant^@^** |
| --- | --- | --- | --- | --- | --- | --- | --- | --- |
| 17:39643658 | rs139865124 | T_A | *KRT36* | 0.0515 | 0.0024 | 21.512 | 0.826 |  |
| 9:6556242 | rs147275962  Benign | C_T | *GLDC* | 0.05 | 0.003 | 16.6933 | 0.814 | Glycine encephalopathy (AR); |
| 22:46664412 | rs117135869 | C_T | *TTC38* | 0.0502 | 0.0058 | 8.6644 | 0.621 |  |
| 1:97981421 | rs1801158  Conflicting interpretation of pathogenicity | C_T | *DPYD* | 0.0642 | 0.0096 | 6.702 | 0.432 | 5-fluorouracil toxicity (AR),  Dihydropyrimidine dehydrogenase deficiency (AR); |
| 7:80141152 | rs143118835 | C_T | *GNAT3* | 0.0528 | 0.006 | 8.8169 | 0.428 |  |
| 21:43985958 | rs117821416 | A_T | *SLC37A1* | 0.0606 | 0.0074 | 8.196 | 0.412 |  |
| 1:39758439 | rs139995582 | G_T | *MACF1* | 0.0825 | 0.0062 | 13.3236 | 0.407 |  |
| 12:101679658 | rs78440807 | G_A | *UTP20* | 0.0554 | 0.0098 | 5.6584 | 0.353 |  |
| 5:176314206 | rs61749651 | T_C | *HK3* | 0.0671 | 0.007 | 9.6065 | 0.32 |  |
| 10:100503793 | rs147866530 | A_G | *HPSE2* | 0.066 | 0.0058 | 11.3927 | 0.297 | Urofacial syndrome 1 (AR); |
| 15:101551007 | rs55739947 | C_A | *LRRK1* | 0.0619 | 0.0084 | 7.3756 | 0.295 |  |
| 2:189940142 | rs76148000  Benign/Likely benign | T_G | *COL5A2* | 0.0503 | 0.01 | 5.0428 | 0.272 | Ehlers-Danlos syndrome classic type (AD); |
| 17:8138569 | rs62624978  Benign/Conflicting interpretation | C_G | *CTC1* | 0.0749 | 0.0076 | 9.8727 | 0.264 | Cerebroretinal microangiopathy with calcifications and cysts (AR); |
| 2:21229905 | rs12720854  Conflicting interpretation | T_C | *APOB* | 0.0516 | 0.0072 | 7.1783 | 0.264 | Hypobetalipoproteinemia (AR); Hypercholesterolemia, due to ligand-defective apo B (AD) |
| 19:52919433 | rs61978639 | G_C | *ZNF528* | 0.067 | 0.0088 | 7.627 | 0.257 |  |
| 5:78338202 | rs77116243  Benign | T_C | *DMGDH* | 0.0536 | 0.0076 | 7.0683 | 0.237 | Dimethylglycine dehydrogenase deficiency (AR); |
| 7:16640454 | rs35504966 | G_A | *ANKMY2* | 0.0672 | 0.0044 | 15.3066 | 0.219 |  |
| 9:139333269 | rs36064831  Benign/Likely Benign | G_C | *INPP5E* | 0.0584 | 0.0078 | 7.4984 | 0.214 | Joubert syndrome 1 (AR),Mental retardation truncal obesity retinal dystrophy and micropenis (AR); |
| 8:52323908 | rs117864200 | A_G | *PXDNL* | 0.0589 | 0.0096 | 6.1482 | 0.207 |  |
| 16:2820684 | rs138495768 | C_T | *SRRM2* | 0.0517 | 0.0096 | 5.3899 | 0.19 |  |
| 19:38673353 | rs61729145 | G_A | *SIPA1L3* | 0.072 | 0.0078 | 9.2416 | 0.186 | Cataract 45 (AR) |
| 4:38828828 | rs5743820 | G_A | *TLR6* | 0.0584 | 0.0096 | 6.0951 | 0.179 |  |
| 9:79938069 | rs41289969 | G_A | *VPS13A* | 0.0567 | 0.0062 | 9.16 | 0.158 | Choreoacanthocytosis (AR); |
| 12:51685707 | rs117691477 | T_C | *BIN2* | 0.0619 | 0.0062 | 9.9927 | 0.151 |  |
| 2:55561944 | rs112409178 | T_A | *CCDC88A* | 0.0517 | 0.0092 | 5.6312 | 0.14 | PEHO syndrome-like (AR); |
| 12:40740686 | rs33995883  Conflicting interpretations | A_G | *LRRK2* | 0.1181 | 0.0098 | 12.0684 | 0.138 | Parkinson disease 8 (AD); |
| 4:95223349 | rs34124298 | A_G | *HPGDS* | 0.0623 | 0.0066 | 9.452 | 0.137 |  |
| 7:141490219 | rs2234013 | G_A | *TAS2R5* | 0.0503 | 0.003 | 16.8092 | 0.135 |  |
| 9:140509075 | rs72765183 | A_C | *ARRDC1* | 0.0519 | 0.0072 | 7.2203 | 0.134 |  |
| 20:24523893 | rs6083553 | G_A | *SYNDIG1* | 0.0741 | 0.0076 | 9.7706 | 0.127 |  |
| 1:70460304 | rs72676879 | A_G | *LRRC7* | 0.0912 | 0.007 | 13.0534 | 0.125 |  |
| 6:111583530 | rs147451538 | G_C | *KIAA1919* | 0.0646 | 0.008 | 8.0849 | 0.117 |  |
| 9:34256375 | rs34101674 | G_T | *KIF24* | 0.0828 | 0.0056 | 14.802 | 0.116 |  |
| 4:100532602 | rs113337987  Conflicting interpretations | G_A | *MTTP* | 0.0651 | 0.0098 | 6.6577 | 0.105 | Abetalipoproteinemia (AR) |
| 19:9226265 | rs75711758 | T_C | *OR7G1* | 0.0609 | 0.0062 | 9.8434 | 0.1 |  |
| 20:30789888 | rs61757724 | G_A | *PLAGL2* | 0.0567 | 0.0024 | 23.6632 | 0.1 |  |
| 7:97861243 | rs200666526 | T_C | *TECPR1* | 0.0659 | 0.0008 | 82.5495 | 0.1 |  |
| 10:7242441 | rs41306403 | G_A | *SFMBT2* | 0.0601 | 0.008 | 7.5292 | 0.097 |  |
| 19:43990795 | rs117243018 | G_A | *PHLDB3* | 0.0681 | 0.0098 | 6.9601 | 0.095 |  |
| 1:109482304 | rs36032236 | G_A | *CLCC1* | 0.0789 | 0.0088 | 8.9749 | 0.089 |  |
| 10:75523634 | rs35528438 | A_G | *SEC24C* | 0.0604 | 0.008 | 7.567 | 0.087 |  |
| 17:38320381 | rs140375987 | C_T | *CASC3* | 0.057 | 0.0008 | 71.3456 | 0.087 |  |
| 15:60758905 | rs141469235  Benign | T_C | *ICE2* | 0.05 | 0.0032 | 15.65 | 0.086 |  |
| 20:56188219 | rs41304401 | C_T | *ZBP1* | 0.0544 | 0.0084 | 6.4849 | 0.086 |  |
| 7:132193335 | rs145024048 | A_G | *PLXNA4* | 0.0714 | 0.006 | 11.9238 | 0.086 |  |
| 3:98001858 | rs138288586 | A_G | *OR5H2* | 0.0594 | 0.0054 | 11.0251 | 0.085 |  |
| 11:18102014 | rs35394666 | G_A | *SAAL1* | 0.0621 | 0.01 | 6.2168 | 0.083 |  |
| 16:684579 | rs34560623 | C_T | *C16orf13* | 0.0979 | 0.0098 | 10.0097 | 0.077 |  |
| 4:438084 | rs192147896 | C_T | *ZNF721* | 0.0729 | 0.0024 | 30.4305 | 0.077 |  |
| 1:54518725 | rs41294776 | G_A | *TMEM59* | 0.0674 | 0.0084 | 8.0338 | 0.076 |  |
| 22:46657308 | rs41302599 | T_C | *PKDREJ* | 0.0519 | 0.0058 | 8.9631 | 0.076 |  |
| 9:138439086 | rs492193 | T_A | *OBP2A* | 0.0644 | 0.0096 | 6.7184 | 0.076 |  |
| 6:46846043 | rs41273668 | T_A | *ADGRF5* | 0.0521 | 0.0072 | 7.2454 | 0.073 |  |
| 8:30938692 | rs4987238  Conflicting interpretation | G_T | *WRN* | 0.0511 | 0.0004 | 127.8451 | 0.072 | Werner syndrome (AR); |
| 6:46661488 | rs41273662 | A_G | *TDRD6* | 0.055 | 0.0072 | 7.6487 | 0.069 |  |
| 1:158943539 | rs112218133 | C_T | *PYHIN1* | 0.086 | 0.0046 | 18.7179 | 0.064 |  |
| 2:96950323 | rs143898031  Benign/Likely Benign | C_T | *SNRNP200* | 0.0657 | 0.0042 | 15.6663 | 0.062 | Retinitis pigmentosa 33 (AD); |
| 3:58395842 | rs140203295 | A_T | *PXK* | 0.0584 | 0.0094 | 6.2248 | 0.062 |  |
| 5:1338078 | rs113203740 | C_T | *CLPTM1L* | 0.0524 | 0.0086 | 6.1083 | 0.062 |  |
| 19:38383767 | rs140149327 | T_G | *WDR87* | 0.0584 | 0.0058 | 10.0884 | 0.061 |  |
| 12:92818773 | rs139979211 | C_T | *CLLU1* | 0.0543 | 0.0042 | 12.9607 | 0.057 |  |
| 18:70417409 | rs12965824 | C_T | *NETO1* | 0.0717 | 0.008 | 8.9741 | 0.056 |  |
| 8:32505615 | rs34822181 | G_C | *NRG1* | 0.0511 | 0.0038 | 13.4574 | 0.053 | Schizophrenia 6 (AD); |
| 2:160876771 | rs141323673 | T_C | *PLA2R1* | 0.05 | 0.0014 | 35.7715 | 0.049 |  |
| 1:87026002 | rs55712824 | C_T | *CLCA4* | 0.0592 | 0.0002 | 296.6404 | 0.048 |  |
| 6:151673070 | rs41289373 | G_A | *AKAP12* | 0.0619 | 0.0064 | 9.6804 | 0.045 |  |
| 19:38056087 | rs45542635 | C_T | *ZNF571* | 0.059 | 0.0066 | 8.9579 | 0.041 |  |
| 17:78166326 | rs61751629  Benign | G_A | *CARD14* | 0.054 | 0.0082 | 6.5968 | 0.033 | Pityriasis rubra pilaris (AD), Psoriasis 2 (AD); |
| 5:56177843 | rs45556841  Benign | C_G | *MAP3K1* | 0.0589 | 0.0074 | 7.9761 | 0.033 | \|  \| 46XY sex reversal 6 \| \| --- \| --- \|   (AD) |
| 19:52934741 | rs118147154 | A_C | *ZNF534* | 0.0507 | 0.0098 | 5.1817 | 0.029 |  |
| 7:6080686 | rs34909691 | A_T | *EIF2AK1* | 0.0552 | 0.009 | 6.1401 | 0.028 |  |
| 22:29940556 | rs61740613 | G_C | *THOC5* | 0.0566 | 0.0096 | 5.9021 | 0.026 |  |
| 10:38121631 | rs11011379 | T_C | *ZNF248* | 0.0502 | 0.0076 | 6.6123 | 0.024 |  |
| 10:121662335 | rs34157476 | G_T | *SEC23IP* | 0.0512 | 0.0082 | 6.2584 | 0.02 |  |
| 14:105420134 | rs45448397 | C_T | *AHNAK2* | 0.069 | 0.008 | 8.6345 | 0.019 |  |
| 16:2160904 | rs140980374  Benign/Likely Benign | C_T | *PKD1* | 0.0739 | 0.0044 | 16.8185 | 0.018 | Polycystic kidney disease 1 (AD) |
| 9:139369079 | rs73670288 | T_C | *SEC16A* | 0.0584 | 0.009 | 6.5014 | 0.017 |  |
| 3:98072962 | rs111341876 | G_A | *OR5K4* | 0.0515 | 0.0046 | 11.2237 | 0.014 |  |
| 9:123675895 | rs113495277 | A_G | *TRAF1* | 0.0852 | 0.0072 | 11.8561 | 0.013 |  |
| 19:21606390 | rs112511886 | G_A | *ZNF493* | 0.066 | 0.0062 | 10.6577 | 0.012 |  |
| 2:215593522 | rs61754118  Benign/Likely Benign | T_C | *BARD1* | 0.0605 | 0.0056 | 10.8205 | 0.011 | Breast cancer familial susceptibility to (AD) multifactorial disorder; |
| 5:156456715 | rs137884244 | C_T | *HAVCR1* | 0.0808 | 0.002 | 40.4425 | 0.01 |  |
| 7:17378917 | rs61755968 | A_G | *AHR* | 0.0619 | 0.0014 | 44.2534 | 0.009 |  |
| 11:55541019 | rs147031394 | G_A | *OR5D13* | 0.0625 | 0.0072 | 8.6944 | 0.004 |  |
| 2:74720140 | rs183395059 | C_G | *TTC31* | 0.0534 | 0.0014 | 38.2385 | 0.003 |  |

^@^, none of the listed variants in seen annotated in OMIM.

**Supplementary Table S7 – The 230 SAFD variants for which annotation was available in ClinVar.**

| **Chr:Position** | **dbSNP** | **Ref_ Alt** | **Gene** | **Function** | **KUWAIT** | | **1kGP** | | **gnomAD** | | **Inherit- -ance mode** | **Disease Name** |
| --- | --- | --- | --- | --- | --- | --- | --- | --- | --- | --- | --- | --- |
|  |  |  |  |  | **KWT_MAF** | **Max_Pop (MAF)** | **1kGP_MAF** | **Max_Pop (MAF)** | **gnomAD_MAF** | **Max_Pop (MAF)** |  |  |
| **Benign** | | | | | | | | | | | | |
| 1:5965455 | rs571655 | C_T | ***NPHP4*** | mis | 0.0292 | KWB (0.0588) | 0.0052 | EUR (0.0189) | 0.0103 | ASJ (0.0304) | AR | Nephronophthisis |
| 1:57340727 | rs652785 | C_A | ***C8A*** | mis | 0.4523 | KWP (0.4797) | 0.3437 | EAS (0.4960) | 0.3654 | SAS (0.4983) | AR | COMPLEMENT COMPONENT 8, ALPHA SUBUNIT, A/B POLYMORPHISM |
| 1:66075952 | rs1805094 | G_C | *LEPR* | mis | 0.2370 | KWP (0.2500) | 0.1424 | AFR (0.1997) | 0.1588 | ASJ (0.2099) | na | LEPTIN RECEPTOR POLYMORPHISM |
| 1:197061086 | rs36004306 | A_C | ***ASPM*** | mis | 0.0632 | KWP (0.0625) | 0.0150 | EUR (0.0417) | 0.0345 | NFE (0.0477) | AR | Primary autosomal recessive microcephaly 5 |
| 1:197070442 | rs3762271 | G_T | ***ASPM*** | mis | 0.3057 | KWP (0.3402) | 0.2143 | EUR (0.4105) | 0.3356 | NFE (0.4321) | AR | Primary autosomal recessive microcephaly 5 |
| 1:197070697 | rs41310927 | T_C | ***ASPM*** | mis | 0.3090 | KWP (0.3427) | 0.2143 | EUR (0.4105) | 0.3364 | NFE (0.4326) | AR | Primary autosomal recessive microcephaly 5 |
| 1:197070707 | rs41308365 | G_A | ***ASPM*** | syn | 0.3125 | KWP (0.3480) | 0.2143 | EUR (0.4105) | 0.3361 | NFE (0.4320) | AR | Primary autosomal recessive microcephaly 5 |
| 1:197072420 | rs41310925 | T_C | ***ASPM*** | syn | 0.3136 | KWP (0.3452) | 0.2131 | EUR (0.4115) | 0.3366 | NFE (0.4338) | AR | Primary autosomal recessive microcephaly 5 |
| 1:197094030 | rs6676084 | C_T | ***ASPM*** | syn | 0.3837 | KWS (0.4340) | 0.2167 | EUR (0.3280) | 0.2802 | NFE (0.3144) | AR | Primary autosomal recessive microcephaly 5 |
| 1:241667523 | rs61737760 | C_T | *FH* | syn | 0.0898 | KWS (0.1415) | 0.0375 | SAS (0.0900) | 0.0347 | SAS (0.0863) | AR | Hereditary cancer-predisposing syndrome |
| 2:26502875 | rs1056389 | T_C | *HADHB* | syn | 0.2284 | KWS (0.3028) | 0.1456 | SAS (0.2270) | 0.2109 | ASJ (0.3207) | AR | Mitochondrial trifunctional protein deficiency |
| 2:26683777 | rs45442103 | G_A | ***OTOF*** | syn | 0.0636 | KWP (0.0714) | 0.0238 | EUR (0.0596) | 0.0460 | ASJ (0.0819) | AR | Deafness, autosomal recessive 9 |
| 2:26741961 | rs13031859 | G_A | ***OTOF*** | mis | 0.4911 | KWP (0.5208) | 0.2700 | AMR (0.4986) | 0.4058 | AMR (0.4981) | AR | Deafness, autosomal recessive 9 |
| 2:44104925 | rs6544718 ^# &^ | C_T | *ABCG8* | mis | 0.1411 | KWS (0.1574) | 0.0771 | EUR (0.1958) | 0.1586 | NFE (0.2204) | AR | Sitosterolemia |
| 2:71012604 | rs7566541 ^# &^ | A_G | ***FIGLA*** | syn | 0.2965 | KWP (0.3240) | 0.2023 | EUR (0.4095) | 0.3516 | NFE (0.4454) | AD | Premature ovarian failure |
| 2:136562472 | rs3739022 | G_A | ***LCT*** | syn | 0.3929 | KWS (0.5340) | 0.2432 | SAS (0.3650) | 0.1694 | SAS (0.2997) | AR | Congenital lactase deficiency |
| 2:166897864 | rs6432860 ^# &^ | G_A | ***SCN1A*** | syn | 0.4948 | KWP (0.5198) | 0.2111 | EUR (0.3091) | 0.2748 | ASJ (0.4020) | AD | Generalized epilepsy with febrile seizures plus |
| 2:215593522 | rs61754118 | T_C | *BARD1* | mis | 0.0605 | KWB (0.0938) | 0.0056 | AMR (0.0130) | 0.0077 | ASJ (0.0279) | AD | Hereditary cancer-predisposing syndrome |
| 2:215645989 | rs28997574 | T_G | *BARD1* | syn | 0.0614 | KWB (0.0938) | 0.0086 | AMR (0.0187) | 0.0089 | ASJ (0.0295) | AD | Hereditary cancer-predisposing syndrome |
| 2:231072709 | rs1365776 ^# &^ | T_C | ***SP110*** | mis | 0.2847 | KWS (0.3411) | 0.1975 | EUR (0.3738) | 0.3144 | FIN (0.4145) | AR | Hepatic venoocclusive disease with immunodeficiency |
| 3:37053568 | rs1799977 | A_G | *MLH1* | mis | 0.2115 | KWS (0.2222) | 0.1296 | EUR (0.3250) | 0.2338 | NFE (0.3199) | AD | Lynch syndrome |
| 3:49454277 | rs10640 | G_A | *AMT* | 3pU | 0.3022 | KWB (0.3382) | 0.2065 | EUR (0.3360) | 0.2638 | FIN (0.4789) | AR | Non-ketotic hyperglycinemia |
| 3:58413518 | rs1126722 | T_G | *PDHB* | 3pU | 0.3832 | KWB (0.4853) | 0.2005 | EUR (0.3748) | 0.2896 | ASJ (0.4949) | na | Pyruvate dehydrogenase complex deficiency |
| 3:58413669 | rs4228 | G_T | *PDHB* | 3pU | 0.3717 | KWB (0.4844) | 0.2047 | EUR (0.3757) | 0.2908 | ASJ (0.4936) | na | Pyruvate dehydrogenase complex deficiency |
| 3:100949842 | rs348867 ^# &^ | A_G | *IMPG2* | syn | 0.2749 | KWP (0.3452) | 0.1871 | SAS (0.2229) | 0.2091 | ASJ (0.3128) | AR | Retinitis Pigmentosa, Recessive |
| 3:133494354 | rs1049296 | C_T | *TF* | mis | 0.2500 | KWS (0.2804) | 0.1564 | EAS (0.2560) | 0.1601 | EAS (0.2593) | AR | Transferrin variant c1/c2 |
| 3:150645351 | rs4680058 ^# &^ | A_G | *CLRN1* | 3pU | 0.3500 | KWP (0.3790) | 0.2222 | SAS (0.3528) | 0.3133 | ASJ (0.4447) | AR | Retinitis Pigmentosa, Dominant |
| 3:179143941 | rs1078749 | A_G | ***GNB4*** | syn | 0.1367 | KWS (0.2083) | 0.0232 | AMR (0.0519) | 0.0333 | ASJ (0.0766) | AD | Charcot-Marie-Tooth disease, dominant intermediate F |
| 3:190106074 | rs3214506 | G_C | ***CLDN16*** | mis | 0.2386 | KWS (0.2594) | 0.1170 | EUR (0.2336) | 0.1940 | ASJ (0.2777) | AR | Primary hypomagnesemia |
| 4:9909923 | rs2280205 | G_A | ***SLC2A9*** | mis | 0.4149 | KWS (0.4429) | 0.2712 | EUR (0.4841) | 0.4438 | ASJ (0.5617) | AD, AR | Familial renal hypouricemia |
| 4:88584148 | rs2615498 | C_T | ***DMP1*** | syn | 0.2655 | KWP (0.2680) | 0.1757 | SAS (0.2832) | 0.1898 | SAS (0.2834) | AR | Hypophosphatemic Rickets, Recessive |
| 4:110914427 | rs4698803 ^# &^ | T_A | *EGF* | mis | 0.2000 | KWS (0.2667) | 0.0795 | EUR (0.2008) | 0.1547 | NFE (0.2163) | na | Renal Hypomagnesemia, Recessive |
| 4:169433444 | rs72695199 | C_T | *PALLD* | syn | 0.1328 | KWS (0.1468) | 0.0573 | EUR (0.1173) | 0.1024 | ASJ (0.1699) | na | Carcinoma of pancreas |
| 5:223646 | rs34635677 | A_T | *SDHA* | mis | 0.0533 | KWS (0.0596) | 0.0186 | SAS (0.0511) | 0.0353 | SAS (0.0585) | AR | Mitochondrial complex II deficiency |
| 5:251178 | rs35502109 | G_A | *SDHA* | syn | 0.0383 | KWS (0.0519) | 0.0020 | EUR (0.0089) | 0.0023 | OTH (0.0066) | AR | Mitochondrial complex II deficiency |
| 5:82815170 | rs61749613 | A_G | ***VCAN*** | mis | 0.0792 | KWS (0.1053) | 0.0200 | EUR (0.0487) | 0.0308 | ASJ (0.0528) | AD | Vitreoretinopathy |
| 5:131915022 | rs28903086 | G_A | *RAD50* | mis | 0.0104 | KWP (0.0160) | 0.0004 | AMR (0.0014) | 0.0017 | ASJ (0.0101) | na | Hereditary cancer-predisposing syndrome |
| 5:138665261 | rs13698 | A_G | *MATR3* | 3pU | 0.0931 | KWS (0.1284) | 0.0224 | EUR (0.0527) | 0.0399 | ASJ (0.0859) | AD | Distal myopathy |
| 5:149460553 | rs216123 | A_G | ***CSF1R*** | syn | 0.4721 | KWS (0.5347) | 0.3353 | EUR (0.5527) | 0.4830 | FIN (0.6999) | AD | Hereditary diffuse leukoencephalopathy with spheroids |
| 5:172660004 | rs72554028 | C_T | ***NKX2-5*** | syn | 0.0292 | KWS (0.0321) | 0.0048 | AMR (0.0101) | 0.0075 | ASJ (0.0336) | AD | Atrial septal defect |
| 5:178634619 | rs423552 ^# &^ | T_C | ***ADAMTS2*** | syn | 0.1856 | KWS (0.2431) | 0.0755 | AFR (0.1241) | 0.0668 | AFR (0.1086) | AR | Ehlers-Danlos syndrome, type vii, autosomal recessive |
| 6:38757615 | rs45529837 | G_A | *DNAH8* | mis | 0.0851 | KWP (0.0894) | 0.0210 | AMR (0.0576) | 0.0248 | OTH (0.0369) | na | Primary ciliary dyskinesia |
| 6:52101844 | rs2397084 | T_C | *IL17F* | mis | 0.0877 | KWP (0.1089) | 0.0331 | EUR (0.0785) | 0.0667 | NFE (0.0959) | na | Familial Candidiasis, Dominant |
| 6:52357260 | rs7757370 ^# &^ | A_C | ***EFHC1*** | 3pU | 0.2382 | KWS (0.3050) | 0.1000 | AFR (0.1596) | 0.1060 | AFR (0.1506) | AD | Juvenile myoclonic epilepsy |
| 6:167344583 | rs13213697 | A_G | ***RNASET2*** | syn | 0.2780 | KWB (0.3387) | 0.1845 | AFR (0.3608) | 0.1639 | AFR (0.3477) | AR | Leukoencephalopathy, cystic, without megalencephaly |
| 7:21658745 | rs72657315 | A_G | ***DNAH11*** | mis | 0.0207 | KWS (0.0324) | 0.0014 | SAS (0.0041) | 0.0036 | ASJ (0.0373) | AR | Primary ciliary dyskinesia |
| 7:34125420 | rs77064045 | G_T | ***BMPER*** | syn | 0.2901 | KWP (0.2875) | 0.1893 | EUR (0.3141) | 0.2414 | NFE (0.2939) | AR | Diaphanospondylodysostosis |
| 7:142655008 | rs8176058 | G_A | *KEL* | mis | 0.0603 | KWS (0.0872) | 0.0124 | EUR (0.0378) | 0.0269 | ASJ (0.0588) | na | KELL K/k BLOOD GROUP POLYMORPHISM |
| 8:10464885 | rs56382513 ^# &^ | C_T | ***RP1L1*** | syn | 0.4718 | KWS (0.5048) | 0.3490 | AFR (0.5250) | 0.3810 | ASJ (0.5150) | AD | Occult macular dystrophy |
| 8:18080001 | rs4987076 | G_A | *NAT1* | mis | 0.1065 | KWS (0.1422) | 0.0170 | SAS (0.0409) | 0.0203 | ASJ (0.0416) | na | NAT1*17 ALLELE |
| 8:48885436 | rs762679 ^# &^ | A_T | ***MCM4*** | mis | 0.1720 | KWS (0.2170) | 0.0859 | EUR (0.1551) | 0.1293 | ASJ (0.1721) | AR | Natural killer cell and glucocorticoid deficiency with DNA repair defect |
| 8:55539395 | rs2293869 | A_T | *RP1* | mis | 0.4569 | KWP (0.4828) | 0.2500 | EUR (0.4264) | 0.3450 | ASJ (0.5299) | AD | RECLASSIFIED - RP1 GENE POLYMORPHISM |
| 8:143996539 | rs4539 | T_C | ***CYP11B2*** | mis | 0.4580 | KWP (0.5360) | 0.3464 | EUR (0.4841) | 0.4244 | ASJ (0.5471) | AR | Corticosterone methyloxidase type 1 deficiency |
| 8:143996553 | rs4546 | G_A | ***CYP11B2*** | syn | 0.4632 | KWP (0.5403) | 0.3454 | EUR (0.4841) | 0.4247 | ASJ (0.5472) | AR | Corticosterone methyloxidase type 2 deficiency |
| 9:27197486 | rs35030851 | G_T | ***TEK*** | mis | 0.1021 | KWS (0.1065) | 0.0337 | SAS (0.0491) | 0.0457 | ASJ (0.0938) | AD | Multiple Cutaneous and Mucosal Venous Malformations |
| 9:27203002 | rs45563539 | G_A | ***TEK*** | syn | 0.1031 | KWP (0.1071) | 0.0339 | SAS (0.0501) | 0.0460 | ASJ (0.0961) | AD | Multiple Cutaneous and Mucosal Venous Malformations |
| 9:107602678 | rs2274873 | G_A | ***ABCA1*** | syn | 0.1460 | KWB (0.1471) | 0.0821 | AFR (0.1006) | 0.0849 | NFE (0.0993) | AD | Familial High Density Lipoprotein Deficiency |
| 10:43615094 | rs1800862 | C_T | ***RET*** | syn | 0.0882 | KWP (0.1389) | 0.0359 | SAS (0.0849) | 0.0454 | SAS (0.0947) | AD | No MEN2 disease |
| 10:69299372 | rs61749224 | T_G | ***CTNNA3*** | syn | 0.0479 | KWB (0.0625) | 0.0148 | SAS (0.0470) | 0.0171 | SAS (0.0357) | AD | Arrhythmogenic right ventricular dysplasia, familial, 13 |
| 10:104836853 | rs35647154 | C_T | ***CNNM2*** | syn | 0.1289 | KWS (0.1468) | 0.0278 | EUR (0.0795) | 0.0540 | ASJ (0.0916) | AD | Renal Hypomagnesemia, Dominant |
| 11:10014085 | rs79470805 | T_C | ***SBF2*** | syn | 0.0365 | KWS (0.0472) | 0.0042 | AMR (0.0101) | 0.0075 | SAS (0.0136) | AR | Charcot-Marie-Tooth disease, type IV |
| 11:20648380 | rs1805091 | G_A | ***SLC6A5*** | mis | 0.2589 | KWS (0.2933) | 0.1627 | EUR (0.2505) | 0.2256 | FIN (0.2973) | AD, AR | Hyperekplexia |
| 11:26681856 | rs117748217 | C_T | ***ANO3*** | syn | 0.0376 | KWS (0.0529) | 0.0056 | AMR (0.0144) | 0.0086 | ASJ (0.0314) | AD | Dystonia |
| 11:103158278 | rs10895391 | C_T | *DYNC2H1* | mis | 0.3979 | KWS (0.4220) | 0.2863 | SAS (0.3998) | 0.3190 | SAS (0.3940) | AR | Short Rib Polydactyly Syndrome |
| 11:108122700 | rs2235006 | T_C | *ATM* | mis | 0.0226 | KWS (0.0509) | 0.0026 | EUR (0.0060) | 0.0010 | OTH (0.0018) | AR | Hereditary cancer-predisposing syndrome |
| 12:51380835 | rs11169654 | C_T | ***SLC11A2*** | 3pU | 0.2735 | KWS (0.2925) | 0.1380 | SAS (0.2628) | 0.1730 | SAS (0.2763) | AR | Hypochromic microcytic anemia with iron overload |
| 12:51381750 | rs2285230 | T_C | ***SLC11A2*** | 3pU | 0.2707 | KWS (0.2784) | 0.1380 | SAS (0.2628) | 0.1746 | SAS (0.2801) | AR | Hypochromic microcytic anemia with iron overload |
| 12:51386066 | rs1048230 | A_G | ***SLC11A2*** | syn | 0.2732 | KWS (0.2890) | 0.1380 | SAS (0.2628) | 0.1706 | SAS (0.2802) | AR | Hypochromic microcytic anemia with iron overload |
| 13:33634983 | rs564481 | C_T | *KL* | syn | 0.3986 | KWS (0.4541) | 0.2492 | AMR (0.5086) | 0.3768 | AMR (0.5420) | AR | Tumoral calcinosis, familial, hyperphosphatemic |
| 13:101020733 | rs61749895 | G_T | *PCCA* | mis | 0.0311 | KWB (0.0455) | 0.0028 | EUR (0.0070) | 0.0080 | NFE (0.0114) | AR | Propionic acidemia |
| 13:111121620 | rs9583500 | C_T | ***COL4A2*** | mis | 0.3368 | KWS (0.4174) | 0.1909 | AFR (0.3578) | 0.1757 | AFR (0.3237) | AD | Porencephaly |
| 14:35872926 | rs1050851 | G_A | ***NFKBIA*** | syn | 0.2085 | KWS (0.2448) | 0.1016 | EUR (0.2217) | 0.1666 | NFE (0.2222) | AD | Ectodermal dysplasia, anhidrotic, with T-cell immunodeficiency |
| 14:50094784 | rs34352773 | T_C | *DNAAF2* | syn | 0.0278 | KWS (0.0463) | 0.0026 | AMR (0.0101) | 0.0025 | ASJ (0.0089) | na | Primary ciliary dyskinesia |
| 14:68264867 | rs12891164 ^#^ | G_A | ***ZFYVE26*** | syn | 0.3351 | KWS (0.3426) | 0.2288 | EUR (0.3777) | 0.2844 | ASJ (0.4273) | AR | Spastic paraplegia |
| 14:68272021 | rs35512910 | C_A | ***ZFYVE26*** | mis | 0.0395 | KWP (0.0516) | 0.0032 | SAS (0.0092) | 0.0035 | SAS (0.0086) | AR | Spastic paraplegia |
| 15:31369123 | rs4779816 ^# &^ | G_A | *TRPM1* | inc | 0.2179 | KWB (0.2879) | 0.1056 | EUR (0.1988) | 0.1526 | NFE (0.1979) | na | Congenital Stationary Night Blindness, Recessive |
| 15:58838038 | rs6084 | C_G | ***LIPC*** | syn | 0.4810 | KWS (0.4861) | 0.3035 | EUR (0.5000) | 0.4273 | ASJ (0.5508) | AR | Hepatic lipase deficiency |
| 15:100794363 | rs4369638 ^# &^ | T_C | ***ADAMTS17*** | syn | 0.3086 | KWS (0.3611) | 0.1785 | EUR (0.2724) | 0.2486 | ASJ (0.3266) | AR | Weill-Marchesani-like syndrome |
| 16:20352618 | rs55772253 | C_A | ***UMOD*** | mis | 0.0696 | KWS (0.1000) | 0.0138 | SAS (0.0368) | 0.0209 | ASJ (0.0640) | AD | Uromodulin-associated kidney disease |
| 16:28944700 | rs35979293 | G_T | *CD19* | syn | 0.3110 | KWS (0.3991) | 0.1871 | EUR (0.3976) | 0.2984 | FIN (0.4414) | AR | Common Variable Immune Deficiency, Recessive |
| 16:50744624 | rs2066842 | C_T | ***NOD2*** | mis | 0.1962 | KWP (0.2061) | 0.1020 | EUR (0.2465) | 0.1889 | NFE (0.2691) | AD | Blau syndrome |
| 16:50745199 | rs2066843 | C_T | ***NOD2*** | syn | 0.2055 | KWP (0.2198) | 0.1076 | EUR (0.2475) | 0.1906 | NFE (0.2707) | AD | Blau syndrome |
| 16:50745583 | rs1861759 | T_G | ***NOD2*** | syn | 0.3523 | KWS (0.4019) | 0.2161 | EUR (0.4165) | 0.3303 | ASJ (0.5324) | AD | Blau syndrome |
| 16:50827518 | rs2066852 | C_T | ***CYLD*** | syn | 0.1615 | KWB (0.1765) | 0.0791 | SAS (0.2045) | 0.0853 | SAS (0.2333) | AD | Spiegler-Brooke syndrome |
| 16:57996960 | rs13336595^# &^ | T_C | *CNGB1* | mis | 0.3776 | KWS (0.3807) | 0.2480 | AFR (0.3200) | 0.2181 | ASJ (0.3169) | AR | Retinitis Pigmentosa, Recessive |
| 16:68856088 | rs33969373 | C_T | *CDH1* | 5pU | 0.1017 | KWS (0.1284) | 0.0228 | AFR (0.0681) | 0.0104 | AFR (0.0506) | AD | Hereditary cancer-predisposing syndrome |
| 16:68867387 | rs2229044 | C_T | *CDH1* | syn | 0.1103 | KWS (0.1606) | 0.0178 | AFR (0.0499) | 0.0090 | AFR (0.0365) | AD | Hereditary cancer-predisposing syndrome |
| 17:1680002 | rs6828^# &^ | C_T | *SERPINF1* | syn | 0.3241 | KWS (0.3367) | 0.2232 | SAS (0.3620) | 0.2692 | SAS (0.3661) | na | Osteogenesis Imperfecta, Recessive |
| 17:7127146 | rs35501596 | G_A | ***ACADVL*** | syn | 0.0155 | KWS (0.0321) | 0.0006 | EUR (0.0020) | 0.0008 | NFE (0.0012) | AR | Very long chain acyl-CoA dehydrogenase deficiency |
| 17:8138569 | rs62624978 | C_G | *CTC1* | mis | 0.0749 | KWS (0.1157) | 0.0076 | AMR (0.0202) | 0.0131 | FIN (0.0213) | AR | Dyskeratosis congenita |
| 17:16855878 | rs8072293 ^# &^ | T_C | ***TNFRSF13B*** | syn | 0.2085 | KWP (0.2240) | 0.1004 | EUR (0.2266) | 0.1937 | ASJ (0.2892) | AD, AR | Common variable immunodeficiency 2 |
| 17:19578873 | rs7216 ^# &^ | T_A | ***ALDH3A2*** | syn | 0.4772 | KWS (0.5263) | 0.3215 | AFR (0.4955) | 0.3811 | ASJ (0.5177) | AR | Sjögren-Larsson syndrome |
| 17:41245900 | rs56012641 | T_G | ***BRCA1*** | mis | 0.0206 | KWS (0.0321) | 0.0002 | SAS (0.0010) | 0.0003 | OTH (0.0007) | AD, Mu | Breast-ovarian cancer, familial 1 |
| 17:41246092 | rs55906931 | A_G | ***BRCA1*** | mis | 0.0223 | KWS (0.0321) | 0.0002 | SAS (0.0010) | 0.0003 | OTH (0.0007) | AD, Mu | Breast-ovarian cancer, familial 1 |
| 17:41251803 | rs56187033 | T_C | ***BRCA1*** | mis | 0.0207 | KWS (0.0324) | 0.0002 | SAS (0.0010) | 0.0003 | OTH (0.0007) | AD, Mu | Breast-ovarian cancer, familial 1 |
| 17:75398498 | rs34587622 | C_T | ***SEPT9*** | mis | 0.1117 | KWS (0.1376) | 0.0497 | EUR (0.1163) | 0.0835 | OTH (0.1174) | AD | Hereditary Neuralgic Amyotrophy (HNA) |
| 18:72999359 | rs55679337 | T_C | ***TSHZ1*** | mis | 0.3402 | KWS (0.3578) | 0.2462 | EUR (0.3499) | 0.3007 | ASJ (0.3520) | AD | Aural atresia, congenital |
| 19:7125519 | rs1799815 | G_A | ***INSR*** | syn | 0.0722 | KWP (0.0873) | 0.0166 | EUR (0.0527) | 0.0507 | ASJ (0.0798) | AR | Leprechaunism syndrome |
| 19:7623941 | rs112133109 | G_A | ***PNPLA6*** | syn | 0.0241 | KWS (0.0367) | 0.0042 | EUR (0.0139) | 0.0107 | NFE (0.0154) | AR | Spastic paraplegia 39 |
| 19:8669931 | rs7255721 ^# &^ | C_G | ***ADAMTS10*** | mis | 0.2085 | KWS (0.2547) | 0.1060 | EUR (0.2952) | 0.2093 | FIN (0.3085) | AR | Weill-Marchesani syndrome |
| 19:18197635 | rs436857 | G_A | ***IL12RB1*** | mis | 0.2056 | KWP (0.2280) | 0.1276 | EUR (0.1809) | 0.1640 | FIN (0.2097) | AR | Familial Atypical Mycobacteriosis, Autosomal Recessive |
| 19:30193182 | rs1048104 | G_C | ***C19orf12*** | 3pU | 0.4725 | KWS (0.5229) | 0.2630 | EUR (0.5765) | 0.4314 | NFE (0.5958) | AR | Neurodegeneration with brain iron accumulation 4 |
| 19:38983180 | rs35364374 | G_T | ***RYR1*** | mis | 0.1241 | KWP (0.1468) | 0.0549 | SAS (0.1697) | 0.0688 | SAS (0.1561) | AD, AR | Central core disease |
| 19:49206674 | rs601338 | G_A | *FUT2* | sg | 0.4845 | KWS (0.4954) | 0.3217 | AFR (0.4909) | 0.3830 | AFR (0.4981) | na | SECRETOR/NONSECRETOR POLYMORPHISM |
| 19:49485548 | rs5464 | G_A | ***GYS1*** | syn | 0.3483 | KWB (0.3971) | 0.2546 | AFR (0.2943) | 0.2686 | ASJ (0.2986) | AR | Glycogen storage disease 0, muscle |
| 19:49519905 | rs1800447 | A_G | *LHB* | mis | 0.1185 | KWS (0.1542) | 0.0579 | EUR (0.0954) | 0.0651 | FIN (0.1053) | AR | LUTEINIZING HORMONE POLYMORPHISM |
| 20:31386347 | rs6058891 ^# &^ | C_T | ***DNMT3B*** | syn | 0.4879 | KWS (0.5229) | 0.2442 | EUR (0.5795) | 0.4199 | NFE (0.5577) | AR | Centromeric instability of chromosomes 1,9 and 16 and immunodeficiency |
| 20:56139403 | rs2070756 ^# &^ | C_T | ***PCK1*** | syn | 0.3358 | KWS (0.4149) | 0.2131 | SAS (0.3292) | 0.2527 | SAS (0.3173) | AR | Phosphoenolpyruvate carboxykinase (GTP) deficiency |
| 22:17589246 | rs879576 | G_A | *IL17RA* | syn | 0.2293 | KWB (0.2941) | 0.1214 | AFR (0.2466) | 0.1137 | AFR (0.2347) | AR | Familial Candidiasis, Recessive |
| 2:48027755 | rs2020912 | T_C | *MSH6* | mis | 0.0362 | KWS (0.0642) | 0.0040 | EUR (0.0129) | 0.0051 | ASJ (0.0100) | AD | Hereditary nonpolyposis colorectal cancer type 5 |
| **Likely Benign** | | | | | | | | | | | | |
| 1:158639351 | rs73020251 | C_G | ***SPTA1*** | syn | 0.0265 | KWB (0.0469) | 0.0046 | AFR (0.0174) | 0.0014 | AFR (0.0190) | AR | Hereditary pyropoikilocytosis |
| 1:171605539 | rs61730974 | A_G | *MYOC* | syn | 0.0571 | KWS (0.0688) | 0.0122 | AMR (0.0360) | 0.0225 | ASJ (0.0852) | AD | Glaucoma |
| 1:183184690 | rs11586699 | C_T | *LAMC2* | mis | 0.1388 | KWS (0.1748) | 0.0409 | EUR (0.0964) | 0.0664 | ASJ (0.1388) | AR | Epidermolysis bullosa, junctional |
| 1:186092103 | rs41317489 | C_T | *HMCN1* | mis | 0.0327 | KWS (0.0505) | 0.0066 | EUR (0.0149) | 0.0094 | NFE (0.0141) | AD | Macular degeneration |
| 1:236990141 | rs2229274 | G_A | *MTR* | mis | 0.0397 | KWB (0.0441) | 0.0084 | EUR (0.0229) | 0.0187 | ASJ (0.0494) | AR | Disorders of Intracellular Cobalamin Metabolism |
| 2:1546327 | rs1042589 | C_G | *TPO* | 3pU | 0.4914 | KWS (0.5229) | 0.3526 | EAS (0.4732) | 0.4150 | ASJ (0.5815) | AR | Congenital hypothyroidism |
| 2:62053279 | rs62148138 | T_G | *FAM161A* | 3pU | 0.1934 | KWS (0.2379) | 0.1114 | EUR (0.2217) | 0.1803 | ASJ (0.2630) | na | Retinitis Pigmentosa, Recessive |
| 2:62067433 | rs17513722 | T_C | *FAM161A* | mis | 0.1959 | KWS (0.2385) | 0.1164 | EUR (0.2227) | 0.1837 | ASJ (0.2626) | na | Retinitis Pigmentosa, Recessive |
| 2:96952833 | rs3171927 | A_G | *SNRNP200* | syn | 0.4397 | KWP (0.4722) | 0.2841 | SAS (0.6994) | 0.3164 | SAS (0.6593) | AD | Retinitis Pigmentosa, Dominant |
| 2:191851646 | rs41270237 | C_T | *STAT1* | syn | 0.0275 | KWS (0.0413) | 0.0018 | SAS (0.0051) | 0.0042 | ASJ (0.0225) | AD | Familial Atypical Mycobacteriosis, Autosomal Dominant |
| 2:202074217 | rs147814983 | T_C | ***CASP10*** | syn | 0.0087 | KWS (0.0184) | 0.0002 | EUR (0.0010) | 0.0003 | OTH (0.0009) | AD | Autoimmune lymphoproliferative syndrome |
| 3:120369683 | rs140977117 | G_A | ***HGD*** | syn | 0.0379 | KWS (0.0459) | 0.0096 | EUR (0.0278) | 0.0233 | FIN (0.0512) | AR | Alkaptonuria |
| 3:130672718 | rs2760272 | T_C | ***ATP2C1*** | syn | 0.0086 | KWB (0.0147) | 0.0002 | EUR (0.0010) | 0.0014 | ASJ (0.0178) | AD | Familial benign pemphigus |
| 4:9828099 | rs144428359 | G_A | ***SLC2A9*** | syn | 0.0292 | KWP (0.0397) | 0.0016 | EUR (0.0070) | 0.0040 | ASJ (0.0149) | AD, AR | Familial renal hypouricemia |
| 4:110667485 | rs41278047 | T_C | ***CFI*** | mis | 0.0242 | KWS (0.0561) | 0.0012 | SAS (0.0031) | 0.0039 | ASJ (0.0479) | AD | Atypical hemolytic uremic syndrome |
| 4:158065029 | rs147320218 | C_T | *GLRB* | syn | 0.0087 | KWS (0.0139) | 0.0002 | EUR (0.0010) | 0.0005 | ASJ (0.0020) | na | Hyperekplexia |
| 5:82836537 | rs149032014 | C_T | ***VCAN*** | mis | 0.0088 | KWS (0.0191) | 0.0002 | AMR (0.0014) | 0.0005 | SAS (0.0022) | AD | Vitreoretinopathy |
| 5:149753865 | rs55918703 | G_A | ***TCOF1*** | syn | 0.0160 | KWP (0.0240) | 0.0010 | AMR (0.0029) | 0.0016 | NFE (0.0024) | AD | Treacher Collins Syndrome, Dominant |
| 5:150646888 | rs61740602 | T_C | ***GM2A*** | mis | 0.1565 | KWS (0.1700) | 0.0563 | SAS (0.1145) | 0.0824 | SAS (0.1259) | AR | Tay-Sachs disease, variant AB |
| 6:39872997 | rs60326448 | T_G | ***MOCS1*** | 3pU | 0.0515 | KWS (0.0505) | 0.0168 | EAS (0.0446) | 0.0147 | EAS (0.0468) | AR | Molybdenum cofactor deficiency |
| 6:39873399 | rs1063171 | G_C | ***MOCS1*** | 3pU | 0.0687 | KWS (0.0780) | 0.0158 | SAS (0.0327) | 0.0282 | ASJ (0.0462) | AR | Molybdenum cofactor deficiency |
| 6:39874728 | rs41273142 | C_T | ***MOCS1*** | 3pU | 0.0498 | KWP (0.0516) | 0.0164 | SAS (0.0327) | 0.0270 | ASJ (0.0435) | AR | Molybdenum cofactor deficiency |
| 6:39881102 | rs7762875 | A_T | ***MOCS1*** | mis | 0.0787 | KWS (0.0990) | 0.0254 | EUR (0.0885) | 0.0635 | NFE (0.0942) | AR | Molybdenum cofactor deficiency |
| 6:112390565 | rs17219737 | A_G | ***WISP3*** | syn | 0.1236 | KWS (0.1616) | 0.0246 | EUR (0.0736) | 0.0428 | NFE (0.0633) | AR | Progressive pseudorheumatoid dysplasia |
| 6:137519588 | rs11914 | A_C | ***IFNGR1*** | syn | 0.1955 | KWP (0.2480) | 0.0940 | EUR (0.1471) | 0.1316 | ASJ (0.2142) | AR | Familial Atypical Mycobacteriosis, Autosomal Recessive |
| 6:152510429 | rs139590550 | G_A | ***SYNE1*** | syn | 0.0344 | KWS (0.0642) | 0.0012 | AMR (0.0043) | 0.0017 | ASJ (0.0058) | AD | Emery-Dreifuss muscular dystrophy |
| 6:152694190 | rs35379711 | T_C | ***SYNE1*** | syn | 0.0260 | KWB (0.0441) | 0.0006 | EUR (0.0020) | 0.0017 | ASJ (0.0120) | AR | Cerebellar ataxia |
| 7:44104788 | rs61756062 | A_C | ***PGAM2*** | mis | 0.1082 | KWS (0.1422) | 0.0547 | SAS (0.1135) | 0.0271 | SAS (0.0975) | AR | Glycogen storage disease type X |
| 7:55233089 | rs17290169 | C_T | *EGFR* | syn | 0.1206 | KWS (0.1651) | 0.0421 | AFR (0.0696) | 0.0516 | ASJ (0.0716) | AR | Lung cancer |
| 7:94039046 | rs34511999 | C_T | *COL1A2* | syn | 0.0309 | KWP (0.0516) | 0.0032 | AMR (0.0072) | 0.0078 | ASJ (0.0157) | AD | Osteogenesis imperfecta |
| 7:130036801 | rs17133175 | G_A | ***CEP41*** | 3pU | 0.2414 | KWP (0.2619) | 0.1408 | SAS (0.2106) | 0.1569 | SAS (0.1954) | AR | Joubert syndrome |
| 7:130036873 | rs73152869 | C_A | ***CEP41*** | 3pU | 0.2440 | KWP (0.2659) | 0.1476 | SAS (0.2106) | 0.1586 | SAS (0.1954) | AR | Joubert syndrome |
| 8:10464604 | rs117007660 | T_C | ***RP1L1*** | mis | 0.0722 | KWS (0.0872) | 0.0156 | EUR (0.0457) | 0.0314 | ASJ (0.0528) | AD | Occult macular dystrophy |
| 8:65527669 | rs59035258 | C_T | ***CYP7B1*** | mis | 0.0712 | KWP (0.0992) | 0.0178 | EUR (0.0427) | 0.0345 | ASJ (0.0983) | AR | Spastic Paraplegia, Recessive |
| 9:27212856 | rs45505400 | C_T | ***TEK*** | syn | 0.0248 | KWS (0.0377) | 0.0020 | EUR (0.0070) | 0.0074 | NFE (0.0131) | AD | Multiple Cutaneous and Mucosal Venous Malformations |
| 9:79936415 | rs17423984 | A_G | ***VPS13A*** | syn | 0.1085 | KWB (0.1250) | 0.0487 | SAS (0.1319) | 0.0719 | SAS (0.1261) | AR | Choreoacanthocytosis |
| 9:79954545 | rs7025532 | T_C | ***VPS13A*** | syn | 0.2544 | KWS (0.2885) | 0.1627 | AFR (0.2307) | 0.1682 | AFR (0.2154) | AR | Choreoacanthocytosis |
| 10:8100599 | rs35508267 | C_T | ***GATA3*** | syn | 0.0448 | KWP (0.0516) | 0.0072 | SAS (0.0245) | 0.0088 | ASJ (0.0343) | AD | Barakat syndrome |
| 10:101595996 | rs17222723 | T_A | ***ABCC2*** | mis | 0.1211 | KWS (0.1840) | 0.0373 | EUR (0.0696) | 0.0450 | ASJ (0.1275) | AR | Dubin-Johnson syndrome |
| 10:101606861 | rs1137968 | G_T | ***ABCC2*** | syn | 0.1280 | KWS (0.1916) | 0.0381 | EUR (0.0696) | 0.0452 | ASJ (0.1279) | AR | Dubin-Johnson syndrome |
| 11:18422487 | rs6498 | C_A | ***LDHA*** | syn | 0.2491 | KWS (0.2844) | 0.0974 | AMR (0.1527) | 0.1458 | NFE (0.1719) | AR | Glycogen storage disease XI |
| 11:44296946 | rs11037928 | C_T | ***ALX4*** | syn | 0.1684 | KWS (0.2037) | 0.0587 | EUR (0.1193) | 0.0898 | NFE (0.1230) | AD | Enlarged parietal foramina |
| 11:62381808 | rs1801144 | G_C | *ROM1* | syn | 0.2892 | KWB (0.3676) | 0.1723 | EUR (0.3161) | 0.2650 | ASJ (0.4058) | AD, AR | Retinitis Pigmentosa, 7, DIGENIC |
| 11:65787666 | rs3829937 | G_A | ***CATSPER1*** | syn | 0.2804 | KWS (0.3224) | 0.1625 | AFR (0.2065) | 0.1525 | AFR (0.2104) | AR | Male infertility |
| 11:67809268 | rs36027301 | C_T | ***TCIRG1*** | mis | 0.0584 | KWP (0.0833) | 0.0170 | EUR (0.0497) | 0.0464 | ASJ (0.1158) | AR | Osteopetrosis |
| 11:95569326 | rs61735577 | G_T | *MTMR2* | syn | 0.0952 | KWB (0.1176) | 0.0421 | SAS (0.0941) | 0.0352 | SAS (0.0906) | AR | Charcot-Marie-Tooth, Type 4 |
| 11:102980324 | rs17301028 | C_T | *DYNC2H1* | syn | 0.1138 | KWP (0.1190) | 0.0411 | SAS (0.0900) | 0.0739 | ASJ (0.1298) | AR | Short Rib Polydactyly Syndrome |
| 11:118004863 | rs45584835 | A_G | ***SCN4B*** | 3pU | 0.2603 | KWS (0.2963) | 0.1595 | EUR (0.3191) | 0.2641 | FIN (0.3522) | AD | Romano-Ward syndrome |
| 11:118005119 | rs868344 | T_C | ***SCN4B*** | 3pU | 0.2554 | KWS (0.2905) | 0.1573 | EUR (0.3181) | 0.2633 | FIN (0.3521) | AD | Long QT syndrome |
| 11:118005622 | rs45460396 | T_G | ***SCN4B*** | 3pU | 0.2603 | KWS (0.2982) | 0.1593 | EUR (0.3181) | 0.2640 | FIN (0.3524) | AD | Long QT syndrome |
| 12:49427175 | rs200639395 | G_T | ***KMT2D*** | syn | 0.0138 | KWB (0.0294) | 0.0006 | AFR (0.0023) | 0.0003 | ASJ (0.0032) | AD | Kabuki syndrome |
| 12:52710276 | rs143467763 | G_A | *KRT83* | syn | 0.0172 | KWB (0.0294) | 0.0012 | SAS (0.0051) | 0.0013 | SAS (0.0062) | AD | Beaded hair |
| 12:109993828 | rs67432283 | T_C | ***MMAB*** | 3pU | 0.1048 | KWP (0.1111) | 0.0268 | EUR (0.0845) | 0.0540 | NFE (0.0837) | AR | Methylmalonic acidemia |
| 13:36909893 | rs148399669 | T_C | ***SPART*** | syn | 0.0493 | KWS (0.0874) | 0.0008 | AMR (0.0029) | 0.0015 | ASJ (0.0195) | AR | Troyer syndrome |
| 13:37393904 | rs193240312 | T_C | *RFXAP* | mis | 0.0340 | KWP (0.0522) | 0.0078 | EUR (0.0199) | 0.0137 | ASJ (0.0505) | AR | SCID due to absent class II HLA antigens |
| 13:52598799 | rs61958802 | G_A | ***ALG11*** | syn | 0.0911 | KWS (0.1055) | 0.0302 | SAS (0.0808) | 0.0427 | SAS (0.0880) | AR | Congenital disorder of glycosylation |
| 14:64685212 | rs45453691 | A_G | *SYNE2* | mis | 0.0292 | KWB (0.0588) | 0.0060 | AMR (0.0159) | 0.0135 | ASJ (0.0433) | AD | Emery-Dreifuss muscular dystrophy |
| 15:31329944 | rs12911350 | G_A | *TRPM1* | syn | 0.1667 | KWS (0.1835) | 0.0891 | EUR (0.1362) | 0.1300 | AMR (0.2020) | na | Congenital Stationary Night Blindness, Recessive |
| 15:100692845 | rs28567966 | A_G | ***ADAMTS17*** | mis | 0.2603 | KWP (0.2540) | 0.1458 | AFR (0.2005) | 0.1410 | ASJ (0.2216) | AR | Weill-Marchesani-like syndrome |
| 16:2114407 | rs34012042 | C_T | ***TSC2*** | syn | 0.0997 | KWS (0.1101) | 0.0351 | EUR (0.0726) | 0.0574 | ASJ (0.1003) | AD | Tuberous sclerosis syndrome |
| 16:30750590 | rs143133981 | C_A | ***SRCAP*** | syn | 0.0194 | KWP (0.0246) | 0.0008 | SAS (0.0020) | 0.0011 | ASJ (0.0042) | AD | Floating-Harbor syndrome |
| 16:88501386 | rs141218390 | C_A | ***ZNF469*** | mis | 0.1614 | KWB (0.1765) | 0.0895 | SAS (0.1554) | 0.0756 | SAS (0.1208) | AR | Corneal fragility keratoglobus, blue sclerae AND joint hypermobility |
| 17:40939472 | rs55751736 | C_T | ***WNK4*** | syn | 0.0207 | KWB (0.0294) | 0.0030 | EUR (0.0060) | 0.0069 | ASJ (0.0164) | AD | Pseudohypoaldosteronism, type 2 |
| 17:57128625 | rs74586224 | A_G | ***TRIM37*** | syn | 0.0464 | KWS (0.0596) | 0.0126 | SAS (0.0317) | 0.0170 | SAS (0.0345) | AR | Mulibrey nanism syndrome |
| 17:59760948 | rs4987050 | A_G | *BRIP1* | syn | 0.0276 | KWS (0.0556) | 0.0002 | EUR (0.0010) | 0.0007 | OTH (0.0029) | AD | Hereditary cancer-predisposing syndrome |
| 17:70119918 | rs202028563 | C_G | ***SOX9*** | mis | 0.0107 | KWS (0.0146) | 0.0004 | AMR (0.0029) | 0.0000 | AMR (0.0001) | AD | Camptomelic dysplasia |
| 18:28956904 | rs36101975 | C_T | ***DSG4*** | syn | 0.2338 | KWB (0.2500) | 0.1216 | SAS (0.1472) | 0.1376 | ASJ (0.1832) | AR | Localized AR Hypotrichosis |
| 18:57026436 | rs1043302 | C_T | ***LMAN1*** | mis | 0.0878 | KWS (0.1068) | 0.0216 | EUR (0.0616) | 0.0461 | ASJ (0.1159) | AR | Combined deficiency of factor V and factor VIII, 1 |
| 19:11492395 | rs377322757 | G_A | ***EPOR*** | syn | 0.0276 | KWS (0.0551) | 0.0014 | EUR (0.0040) | 0.0008 | SAS (0.0022) | AD | Familial erythrocytosis |
| 19:11556233 | rs200168017 | G_A | ***PRKCSH*** | mis | 0.0172 | KWS (0.0229) | 0.0004 | AMR (0.0014) | 0.0005 | ASJ (0.0037) | AD | Polycystic liver disease |
| 19:15302790 | rs114457076 | G_A | ***NOTCH3*** | syn | 0.0309 | KWS (0.0459) | 0.0022 | SAS (0.0061) | 0.0020 | ASJ (0.0081) | AD | Cerebra arteriopathy with subcortical infarcts & leukoencephalopathy |
| 19:17953950 | rs55778349 | G_C | *JAK3* | mis | 0.0248 | KWP (0.0248) | 0.0018 | EUR (0.0050) | 0.0059 | FIN (0.0110) | AR | Severe combined immunodeficiency disease |
| 19:18188408 | rs11575926 | C_T | ***IL12RB1*** | mis | 0.1626 | KWP (0.1825) | 0.0559 | EUR (0.1551) | 0.1274 | ASJ (0.1925) | AR | Familial Atypical Mycobacteriosis |
| 19:54403703 | rs35079513 | C_G | ***PRKCG*** | syn | 0.0854 | KWS (0.1509) | 0.0172 | AFR (0.0514) | 0.0125 | AFR (0.0519) | AD | Spinocerebellar Ataxia |
| 20:6069723 | rs35413391 | G_A | ***FERMT1*** | syn | 0.0952 | KWS (0.1111) | 0.0317 | EUR (0.0825) | 0.0546 | ASJ (0.1416) | AR | Kindler's syndrome |
| 20:25060143 | rs140122268 | G_C | *VSX1* | mis | 0.0189 | KWS (0.0321) | 0.0022 | EUR (0.0050) | 0.0034 | ASJ (0.0182) | AD | Posterior Polymorphous Corneal Dystrophy |
| 20:31388080 | rs17123657 | C_T | ***DNMT3B*** | syn | 0.1159 | KWB (0.2500) | 0.0553 | AFR (0.1944) | 0.0158 | AFR (0.1827) | AR | Centromeric instability of chromosomes 1,9 and 16 and immunodeficiency |
| 20:57020228 | rs1059794 | G_C | ***VAPB*** | 3pU | 0.1466 | KWB (0.2059) | 0.0803 | AFR (0.1430) | 0.0858 | AFR (0.1452) | AD | Spinal Muscular Atrophy, Dominant |
| 20:61459319 | rs142412708 | C_T | ***COL9A3*** | syn | 0.0206 | KWS (0.0367) | 0.0012 | SAS (0.0041) | 0.0020 | ASJ (0.0217) | AD | Multiple Epiphyseal Dysplasia, Dominant |
| 21:43176799 | rs13049286 | T_G | ***RIPK4*** | syn | 0.2302 | KWS (0.2752) | 0.1264 | SAS (0.1748) | 0.1293 | ASJ (0.2094) | AR | Popliteal pterygium syndrome |
| 21:43176832 | rs2277791 | A_G | ***RIPK4*** | syn | 0.2276 | KWS (0.2706) | 0.1260 | SAS (0.1748) | 0.1293 | ASJ (0.2094) | AR | Popliteal pterygium syndrome |
| 22:31013399 | rs76802001 | G_A | ***TCN2*** | syn | 0.0481 | KWS (0.0596) | 0.0144 | AMR (0.0389) | 0.0254 | NFE (0.0361) | AR | Transcobalamin II deficiency |
| 22:32506104 | rs33943816 | G_A | ***SLC5A1*** | syn | 0.0825 | KWS (0.0826) | 0.0333 | EUR (0.0577) | 0.0460 | ASJ (0.1004) | AR | Congenital glucose-galactose malabsorption |
| 22:37273805 | rs1858 | C_T | ***NCF4*** | syn | 0.0842 | KWB (0.2273) | 0.0375 | AFR (0.1210) | 0.0125 | AFR (0.1076) | AR | Chronic granulomatous disease |
| 22:44342116 | rs2294918 ^# &^ | G_A | *PNPLA3* | mis | 0.3694 | KWS (0.4541) | 0.2123 | EUR (0.3708) | 0.3211 | ASJ (0.4469) | na | Susceptibility to Nonalcoholic Fatty Liver Disease |
| 22:45685002 | rs1135360 ^# &^ | G_A | *UPK3A* | syn | 0.4819 | KWS (0.5143) | 0.3357 | EUR (0.5676) | 0.5023 | ASJ (0.5886) | na | Renal adysplasia |
| 22:45691594 | rs1057356 ^# &^ | G_A | *UPK3A* | syn | 0.4878 | KWS (0.5280) | 0.3718 | EUR (0.6402) | 0.5484 | FIN (0.6641) | na | Renal adysplasia |
| **Pathogenic** | | | | | | | | | | | | |
| 8:143994266 | rs61757294 | A_G | ***CYP11B2*** | mis | 0.1519 | KWS (0.1698) | 0.0531 | EUR (0.1093) | 0.0846 | ASJ (0.1163) | AR | Corticosterone methyloxidase type 2 deficiency |
| 9:116153891 | rs1800435 | C_G | ***ALAD*** | mis | 0.1241 | KWB (0.1912) | 0.0635 | SAS (0.1585) | 0.0830 | ASJ (0.2207) | AR | AMINOLEVULINATE DEHYDRATASE, ALAD*1/ALAD*2 POLYMORPHISM |
| 10:54531242 | rs5030737 | G_A | ***MBL2*** | mis | 0.0790 | KWP (0.0902) | 0.0272 | EUR (0.0596) | 0.0558 | ASJ (0.1032) | AD | Mannose-binding protein deficiency |
| 10:101829514 | rs61751507 | C_T | ***CPN1*** | mis | 0.0747 | KWS (0.0981) | 0.0266 | AMR (0.0634) | 0.0423 | AMR (0.0668) | AR | Anaphylotoxin inactivator deficiency |
| 15:100230557 | **rs121918530** | A_G | ***MEF2A*** | mis | 0.0103 | KWB (0.0294) | 0.0004 | EUR (0.0020) | 0.0008 | NFE (0.0015) | AD | Coronary artery disease/myocardial infarction |
| 16:31105945 | rs61742245 | C_A | *VKORC1* | mis | 0.0104 | KWB (0.0294) | 0.0004 | EUR (0.0010) | 0.0024 | ASJ (0.0384) | AD | Warfarin response |
| 17:12899902 | rs5030739 | C_T | ***ELAC2*** | mis | 0.0842 | KWS (0.1055) | 0.0232 | SAS (0.0501) | 0.0349 | ASJ (0.0510) | AR | Prostate cancer, hereditary, 2 |
| 17:12915009 | rs4792311 | G_A | ***ELAC2*** | mis | 0.3552 | KWP (0.3611) | 0.2145 | EUR (0.3151) | 0.2742 | ASJ (0.3699) | AR | Prostate cancer, hereditary, 2 |
| 17:79767715 | rs1801483 | G_A | *GCGR* | mis | 0.0378 | KWS (0.0505) | 0.0042 | EUR (0.0149) | 0.0075 | ASJ (0.0120) | AD | Diabetes mellitus type 2 |
| 18:55373793 | rs34719006 | C_T | ***ATP8B1*** | mis | 0.0258 | KWS (0.0413) | 0.0018 | AFR (0.0045) | 0.0031 | ASJ (0.0096) | AD | Cholestasis of pregnancy |
| **Likely Pathogenic or uncertain significance** | | | | | | | | | | | | |
| 2:210742714 | rs187089611 | G_C | *UNC80* | mis | 0.0226 | KWS (0.0417) | 0.0004 | AMR (0.0014) | 0.0002 | OTH (0.0011) | AR | Inborn genetic diseases |
| 10:50958895 | rs150231967 | T_A | *OGDHL* | mis | 0.0122 | KWS (0.0278) | 0.0006 | SAS (0.0020) | 0.0026 | FIN (0.0060) | na | Inborn genetic diseases |
| 12:66935707 | rs199768740 | C_T | *GRIP1* | mis | 0.0242 | KWS (0.0467) | 0.0004 | EUR (0.0020) | 0.0012 | ASJ (0.0060) | AR | Abnormality of brain morphology |
| 14:74953134 | rs151220873 | C_T | *NPC2* | mis | 0.0172 | KWP (0.0238) | 0.0010 | SAS (0.0031) | 0.0022 | ASJ (0.0196) | AR | Brain atrophy |
| 19:13010520 | rs8012^# &^ | G_A | ***GCDH*** | mis | 0.4143 | KWB (0.4412) | 0.2796 | EUR (0.4781) | 0.3895 | FIN (0.5388) | AR | Glutaric aciduria, type 1 |
| 11:68174189 | rs4988321 | G_A | ***LRP5*** | mis | 0.0830 | KWS (0.1019) | 0.0184 | AMR (0.0418) | 0.0379 | ASJ (0.0931) | AR | Osteoporosis with pseudoglioma |
| 10:96798749 | rs10509681 | T_C | ***CYP2C8*** | mis | 0.1198 | KWS (0.1355) | 0.0457 | EUR (0.1183) | 0.0838 | NFE (0.1150) | na | rosiglitazone response - Dosage |
| 12:21331549 | rs4149056 | T_C | ***SLCO1B1*** | mis | 0.1810 | KWS (0.2176) | 0.0877 | EUR (0.1610) | 0.1326 | FIN (0.2109) | Ic | rosuvastatin response - Other |
| **Drug Response** | | | | | | | | | | | | |
| 8:18257854 | rs1801280 | T_C | ***NAT2*** | mis | 0.3927 | KWP (0.3879) | 0.2927 | EUR (0.4493) | 0.3821 | FIN (0.4668) | AR | Slow acetylator due to N-acetyltransferase enzyme variant |
| 10:96702047 | rs1799853 ^&^ | C_T | *CYP2C9* | mis | 0.1181 | KWS (0.1262) | 0.0479 | EUR (0.1243) | 0.0926 | ASJ (0.1357) | AD | Warfarin response |
| 19:15990431 | rs2108622 | C_T | *CYP4F2* | mis | 0.4102 | KWS (0.4541) | 0.2368 | SAS (0.4131) | 0.2735 | SAS (0.3978) | na | acenocoumarol response - Dosage |
| **Risk Factor** | | | | | | | | | | | | |
| 2:138759649 | **rs11558538** | C_T | *HNMT* | mis | 0.1259 | KWS (0.1495) | 0.0595 | SAS (0.1053) | 0.1008 | FIN (0.1601) | AD | Asthma, susceptibility to |
| 4:100268190 | rs283413 ^#^ | C_A | *ADH1C* | sg | 0.0653 | KWP (0.1071) | 0.0072 | SAS (0.0174) | 0.0157 | ASJ (0.0633) | Ic, Mu | Parkinson disease, mitochondrial |
| 5:95751785 | rs6232 | T_C | ***PCSK1*** | mis | 0.0594 | KWB (0.0909) | 0.0210 | SAS (0.0501) | 0.0390 | SAS (0.0659) | Ic | Body mass index quantitative trait locus 12 |
| 10:64415184 | rs7076156 ^#^ | G_A | *ZNF365* | mis | 0.3351 | KWS (0.4450) | 0.1288 | EUR (0.2734) | 0.2044 | ASJ (0.2795) | na | Uric acid nephrolithiasis, susceptibility to |
| 14:104165753 | rs861539 | G_A | ***XRCC3*** | mis | 0.3864 | KWS (0.4450) | 0.2169 | EUR (0.3936) | 0.2904 | ASJ (0.4015) | AD | Cutaneous malignant melanoma 6 |
| 17:5485367 | rs12150220 | A_T | ***NLRP1*** | mis | 0.4377 | KWP (0.4643) | 0.1921 | EUR (0.4443) | 0.3674 | ASJ (0.4744) | AR | Vitiligo-associated multiple autoimmune disease susceptibility 1 |
| 17:48437456 | rs6504649 | C_G | *XYLT2* | mis | 0.4414 | KWB (0.4706) | 0.2510 | EUR (0.4006) | 0.3312 | ASJ (0.4536) | AR | Pseudoxanthoma elasticum, modifier of severity of |
| **Affects** | | | | | | | | | | | | |
| 12:14993439 | rs11276 | C_T | *ART4* | mis | 0.4392 | KWP (0.4715) | 0.2927 | SAS (0.3855) | 0.3476 | SAS (0.3953) | na | Blood group, Dombrock system |
| **Association** | | | | | | | | | | | | |
| 2:27730940 | rs1260326 ^# &^ | C_T | *GCKR* | mis | 0.3945 | KWS (0.4352) | 0.2933 | EAS (0.4812) | 0.3667 | ASJ (0.5344) | na | Fasting plasma glucose level quantitative trait locus 5 |
| 11:68846399 | rs35264875 | A_T | *TPCN2* | mis | 0.1832 | KWP (0.2260) | 0.0996 | SAS (0.2055) | 0.1593 | FIN (0.2980) | na | Skin/hair/eye pigmentation, variation in, 10 |
| **Protective** | | | | | | | | | | | | |
| 4:100260789 | rs698 | T_C | *ADH1C* | mis | 0.3137 | KWS (0.3515) | 0.2143 | EUR (0.4046) | 0.3470 | FIN (0.5169) | Ic, Mu | Alcohol dependence |
| 4:100263965 | rs1693482 | C_T | *ADH1C* | mis | 0.3296 | KWS (0.3830) | 0.2143 | EUR (0.4046) | 0.3462 | FIN (0.5167) | Ic, Mu | Alcohol dependence |

^#^ The alternate allele of this variant is the reference allele in 1000 Genomes phase 3 and gnomAD datasets;

^&^ The clinical or risk allele is the reference allele, whereas for all others it is alternate allele;

Disease names corresponding to gene names in bold denote single gene disorders;

Function: mis – missense, syn – synonymous, 3pU – 3primeUTR, 5Pu – 5primeUTR, sg – stop gained, inc – initiator codon;

Inheritance mode: na – not available, AD – autosomal dominant, AR – autosomal recessive, DR – digenic recessive, Mu – multifactorial, Ic – isolated cases;

Kuwaiti: KWT – All Kuwaitis, KWB – Bedouins, KWP – Persians, KWS – Saudi Arabian tribe;

1kGP global populations: AFR – African, AMR – Admixed American, EAS – East Asian, EUR – European, SAS – South Asian;

gnomAD global populations: AFR – African/African American, AMR – Admixed American, ASJ – Ashkenazi Jewish, EAS – East Asian, FIN – Finnish, NFE – Non-Finnish European, OTH – Other population not assigned, SAS – South Asian.

**Supplementary Figure S1. Kuwaiti population subgroup-wide distribution of total number of SNVs upon step-wise addition of exomes.** The solid line represents the number of all variants found as the number of sequenced exomes increase; the dashed line corresponds to population-specific variants. The green corresponds to the KWS subgroup of Saudi Arabian tribe ancestry; orange corresponds to KWP subgroup of Persian ancestry; and the red corresponds to the nomadic Bedouin subgroup of KWB.


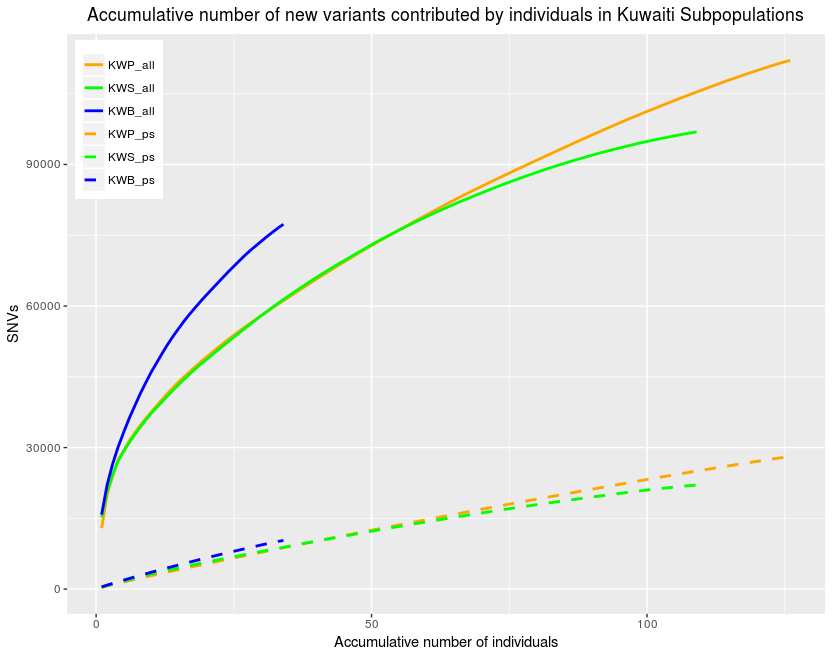


**Supplementary Figure S2. The Manhattan plot showing highly significant differentiating variants (labelled red circles) among Kuwaiti subpopulations.** x-axis: chromosome numbers; y-axis: negative log10pFst values. Blue line indicates the suggestive line of high significant threshold.


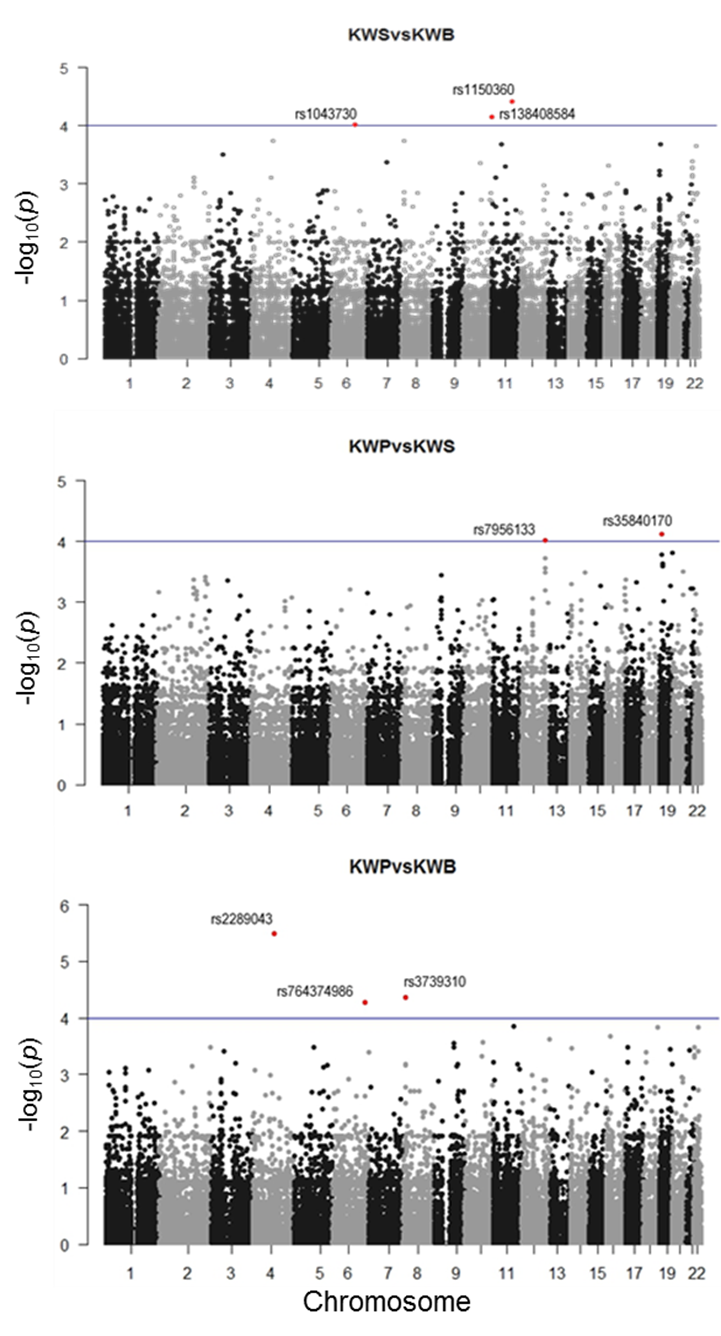


**Supplementary Figure S3. A & B. Scatter plots showing the pairing of Kuwaiti populations with the gnomAD (A) or 1kGP (B) global populations as maximum allele frequency populations.** y-axis: gnomAD (A) or 1kGP (B) gnomAD global population minor allele frequency. (a) x-axis: Overall Kuwaiti population minor allele frequency, (b) x-axis: KWB minor allele frequency; (c) x-axis: KWP minor allele frequency; (d) x-axis: KWS minor allele frequency. gnomAD global populations: AFR – Africans/African Americans, AMR – Admixed Americans, ASJ – Ashkenazi Jewish, EAS – East Asians, FIN – Finnish, NFE – Non-Finnish Europeans, OTH – Other population not assigned, SAS – South Asians. 1kGP global populations: AFR – African, AMR – Admixed American, EAS – East Asian, EUR – European, SAS – South Asian.


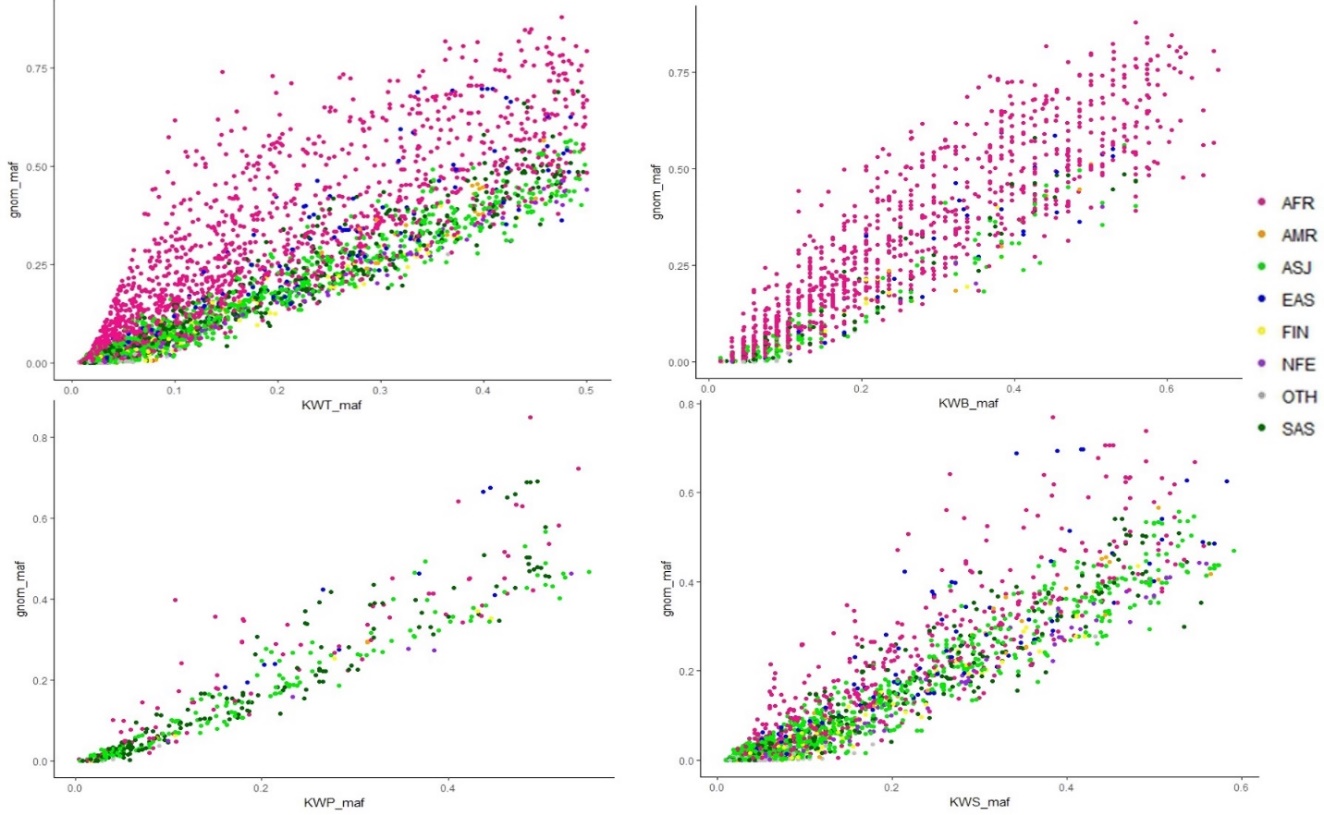


**A**


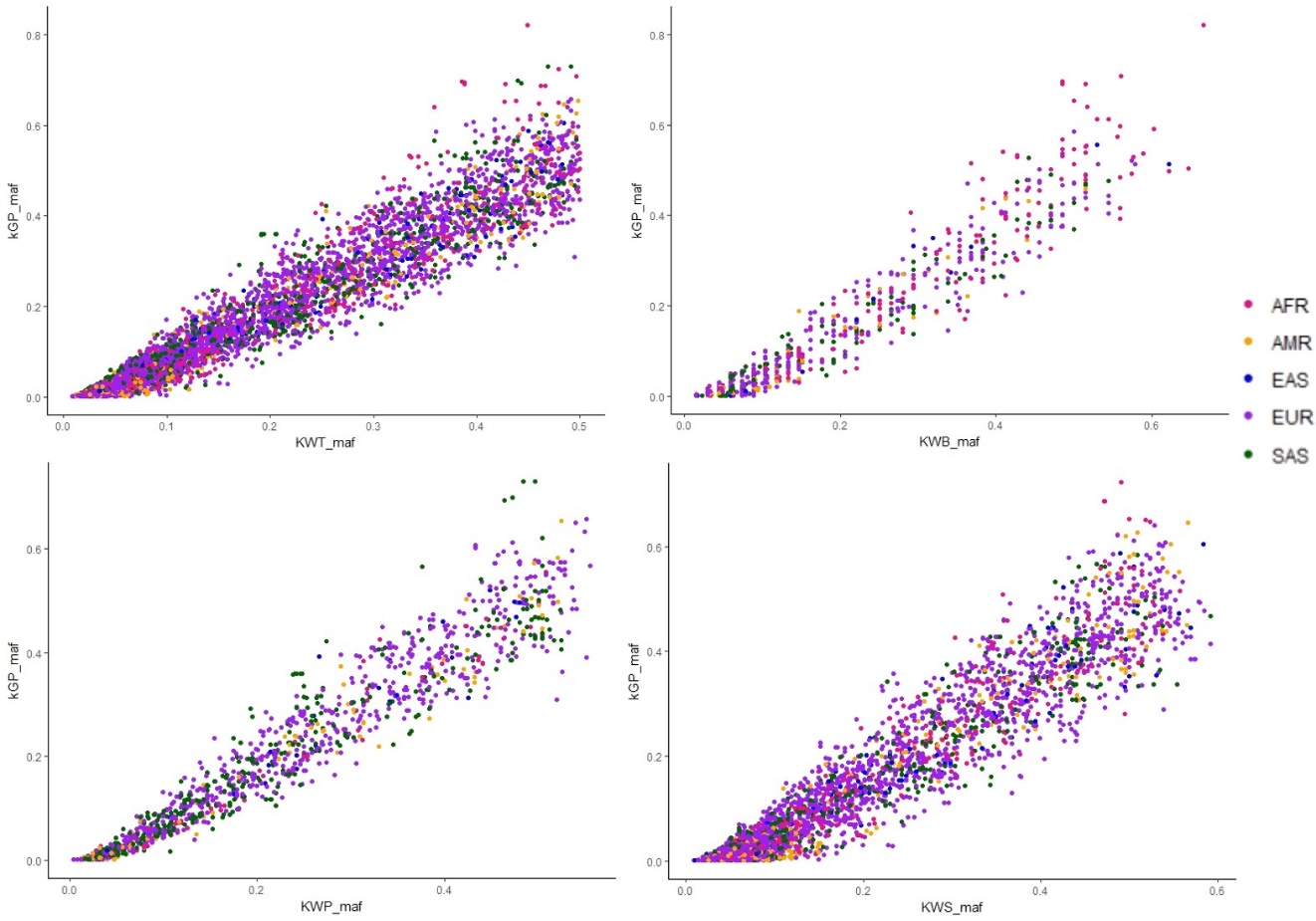


**B**

**Supplementary Figure S4. Heatmap of mean pairwise *F*_ST_ comparison of Kuwaiti subpopulation groups with population groups from 1000 Genomes project phase3, Qatar and Ashkenazi Jews.**

**
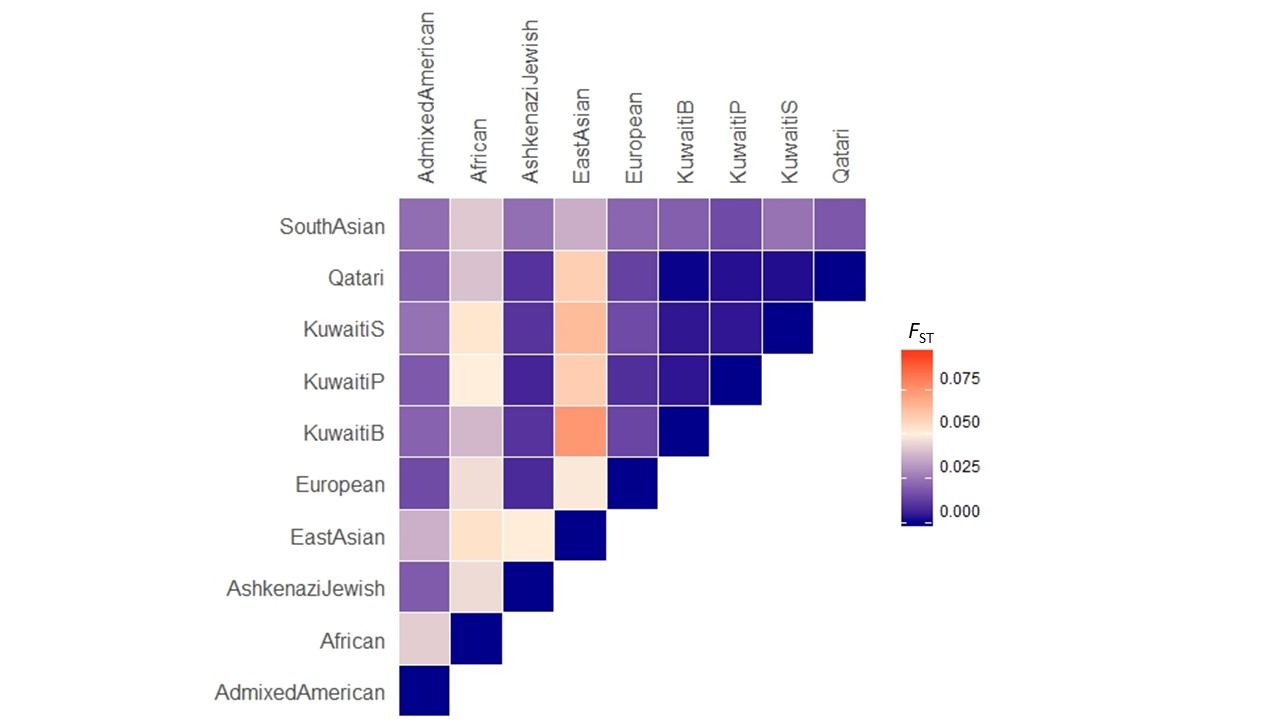
**

**Supplementary Figure S5. Flowchart illustrating the identification of clinically relevant SAFD variants – considering 1kGP global populations.**

**6186** [MAF <1% = 150; <5% = 2288]

**ClinVar Scan**

**1067** [MAF <1% = 17; <5% = 354]

***Disease Name* – “not provided”, “not specified” & “blank space”**

**335** [MAF <1% = 6; <5% = 139]

***Allele origin* – “Other” & “unknown”**

**331** [MAF <1% = 6; <5% = 135]

***Clinical significance* – “Conflicting interpretations of pathogenicity”, “Other”, “Not provided” & “uncertain significance”**

**230** [MAF <1% = 4; <5% = 61]

***Clinical significance* – “Benign” & “Likely Benign”**

**34** [MAF <1% = 0; <5% = 9]
